# The Phlorizin-Degrading *Bacillus licheniformis* XNRB-3 Mediates Soil Microorganisms to Alleviate Apple Replant Disease

**DOI:** 10.3389/fmicb.2022.839484

**Published:** 2022-03-03

**Authors:** Yanan Duan, Lei Zhao, Weitao Jiang, Ran Chen, Rong Zhang, Xuesen Chen, Chengmiao Yin, Zhiquan Mao

**Affiliations:** National Key Laboratory of Crop Biology, College of Horticulture Science and Engineering, Shandong Agricultural University, Shandong, China

**Keywords:** apple replant disease, *Fusarium* spp., biocontrol, biolog, endophytic *Bacillus*, antibiosis

## Abstract

In this study, an endophytic phlorizin-degrading *Bacillus licheniformis* XNRB-3 was isolated from the root tissue of healthy apple trees, and its control effect on apple replant disease (ARD) and how it alleviates the pathogen pressure via changes in soil microbiomes were studied. The addition of strain XNRB-3 in *Fusarium* infested soils significantly reduced the number of pathogens in the soil, thus resulting in a lower disease incidence, and the relative control effect on *Fusarium oxysporum* reached the highest of 66.11%. The fermentation broth can also protect the roots of the plants from *Fusarium oxysporum*, *Fusarium moniliforme*, *Fusarium proliferatum*, and *Fusarium solani* infection. These antagonistic effects were further validated using an *in vitro* assay in which the pathogen control was related to growth and spore germination inhibition via directly secreted antimicrobial substances and indirectly affecting the growth of pathogens. The secreted antimicrobial substances were identified using gas chromatography-mass spectrometry (GC-MS) technology. Among them, alpha-bisabolol and 2,4-di-tert-butylphenol had significant inhibitory effects on many planted pathogenic fungi. Butanedioic acid, monomethyl ester, and dibutyl phthalate promoted root development of Arabidopsis plants. Strain XNRB-3 has multifarious plant growth promoting traits and antagonistic potential. In pot and field experiments, the addition of strain XNRB-3 significantly promoted the growth of plants, and the activity of enzymes related to disease resistance [superoxide dismutase (SOD), peroxidase (POD), and catalase (CAT)] was also significantly enhanced. It also reduced the abundance of four species of *Fusarium* and the content of phenolic acids in the rhizosphere soil, improved soil microbial community structure and nutritional conditions, and increased soil microbial diversity and activity, as well as the soil enzyme activity. The above results indicated that *B. licheniformis* XNRB-3 could be developed into a promising biocontrol and plant-growth-promoting agent.

## Introduction

Apple replant disease (ARD) is a soil sickness caused by biotic and abiotic factors. It occurs in major apple cultivation areas worldwide and poses a major threat to the development of the apple industry (Laurent et al., 2010; Tewoldemedhin et al., 2011). This disease is most harmful to young replanted trees, and it can reduce the growth of trees, increase the susceptibility of trees to diseases, discolor roots, result in root tip necrosis, and reduce root biomass, which can ultimately lead to plant death within the first growing season. Furthermore, the yield and quality of fruit trees more than 20 years old is decreased in plants with ARD, and death can occur in severe cases (Mazzola and Manici, 2012; Yim et al., 2020; Balbín-Suárez et al., 2021). An increasing number of studies have shown that biotic factors are the main factors that cause ARD, including nematodes (*Pratylenchus* spp.), fungi (*Rhizoctonia solani*, *Fusarium* spp., and *Cylindrocarpon* spp.), and oomycetes (*Pythium* and *Phytophthora*) (Weerakoon et al., 2012; Hewavitharana and Mazzola, 2016; Sun et al., 2017; Tilston et al., 2020). Abiotic factors may also play an important role in replanting problems, such as soil structure, the accumulation of phenolic compounds or phytotoxins in disease-affected roots, and nutrition (Hofmann et al., 2009; Wang et al., 2018).

Previous studies have suggested that *Fusarium* spp. is one of the main pathogens contributing to the occurrence of ARD in China, South Africa, and Italy (Kelderer et al., 2012; Yin et al., 2017; Wang et al., 2018; Sheng et al., 2020; Duan et al., 2021). *Fusarium* survives in soil in the absence of hosts for up to 10 years as chlamydospores, and, as a consequence, traditional methods of control such as crop rotation are typically ineffective (Liu L. et al., 2018). Yin et al. (2017) found that root exudates can stimulate the germination of *Fusarium* conidia, and the infectious hyphae from the conidia penetrate the roots of the host plant, resulting in plant death. Therefore, conidia are the most important structures mediating the survival of pathogens and initial infection, they are also some of the direct targets for the biological control of *Fusarium*. Currently, chemical fungicides and soil disinfestation with methyl bromide are the main commercially available approaches for controlling ARD (Dandurand et al., 2019). However, methyl bromide is now being phased out from agriculture because of environmental contamination and associated health threats (Raymaekers et al., 2020). The application of biological control agents (BCAs) is considered an eco-friendly and sustainable method for reducing the effects of plant diseases and has become increasingly used in many countries (Zalila-Kolsi et al., 2016; Chowdhury et al., 2020).

Plant growth-promoting rhizobacteria (PGPR) are a group of bacteria that colonize plant roots and promote plant growth and development (Kloepper and Beauchamp, 1992). Many diverse bacterial genera, such as *Bacillus*, *Klebsiella*, and *Pseudomonas*, can colonize various plant organs (Reiter and Sessitsch, 2006; Santoyo et al., 2016). These bacteria can stimulate plant growth, increase yield, reduce pathogen infection, and reduce biotic or abiotic plant stress under a wide range of environmental conditions without conferring pathogenicity, making them prime candidate BCAs for their ability to effectively control many different plant diseases in crops (Compant et al., 2010; Kumar et al., 2012; Ji et al., 2014; Liu et al., 2019). Bacteria of the genus *Bacillus* produce heat and desiccation-resistant spores, and are thus more suitable for use in biofungicides (Pérez-García et al., 2011).

To continue, BCAs control plant pathogens and harmful microorganisms through the production of antibiotics, antifungal metabolites, and the degradation of the cell wall by different enzymes (Santoyo et al., 2012; Chowdhury et al., 2015; Eljounaidi et al., 2016; Afzal et al., 2019). In addition to antifungal activity, they can also facilitate plant growth through N_2_ fixation; the solubilization of insoluble phosphorus; the production of siderophores, phytohormones (e.g., IAA), and volatile organic compounds (VOCs); and induced systemic resistance (Ahemad and Khan, 2011; Gao et al., 2017). The 2-nonanone and 2-heptanone produced by *B. amyloliquefaciens* L3 isolated from the rhizosphere of watermelon have been shown to have strong antifungal properties against *F. oxysporum* f. sp. *Niveum* (FON), the VOCs acetoin and 2,3-butanediol produced by it promote plant growth (Wu et al., 2019). Marques et al. (2010) found that all the bacterial isolates produce indole acetic acid, hydrogen cyanide, and ammonia when tested *in vitro* for their plant growth-promoting (PGP) abilities. Azabou et al. (2020) isolated a *B. velezensis* OEE1 from root tissue with a strong control effect on *Verticillium* wilt that can significantly reduce the final mean disease severity index (FMS), percentage of dead plants (PDP), and area under disease progress curve (AUDPC). However, there are relatively few biological control agents for *Bacillus* that can be used to control ARD.

In this study, a strain of *B. licheniformis* XNRB-3 that degrades phlorizin was isolated from the root tissue of healthy apple trees in a replanted orchard. The purpose of this study is to (a) characterize the antagonistic activity of the bacterial isolate XNRB-3 against *F. oxysporum*, *F. moniliforme*, *F. proliferatum*, and *F. solani*; (b) evaluate the ability of strain XNRB-3 to degrade phlorizin and promote plant growth; (c) optimize its biocontrol activity against ARD; (d) evaluate the root colonization ability under greenhouse conditions; (e) identify the main antimicrobial compounds involved in its antifungal activity; and (f) verify the effect of XNRB-3 against ARD under outdoor potting and field conditions.

## Materials and Methods

### Microorganisms and Growth Conditions

The culture of plant fungal pathogen (*Fusarium proliferatum*, *Fusarium verticillioides*, *Fusarium oxysporum*, *Fusarium solani*, *Rhizoctonia solani*, *Alternaria alternata*, *Albifimbria verrucaria*, *Aspergillus flavus*, *Penicillium brasilianum*, and *Phytophthora cactorum*) are used in this study. Details of the fungal plant pathogen cultures are shown in Supplementary Table 1. Stock cultures were maintained on potato dextrose agar (PDA) plates at 4°C. Pre cultures were established by transferring a stock agar plug containing mycelia onto fresh PDA plates and incubating for 6 days at 28°C.

### Isolation and Screening of Bacteria for Biocontrol Activity

The rhizosphere soil and root system were taken from healthy apple trees in the replanted orchard [replanted for 4–5 years on old apple orchards (more than 25 years old)] in the Northwest Loess region. The repeated cropping of the sampling orchard was serious, and there were dead trees. The basic information of the sampled sites are presented in Supplementary Table 2. The isolation of antagonistic bacteria refers to the method of Filippi et al. (2011), Fang et al. (2020), and Duan et al. (2021). Morphologically distinct colonies were replicated, and purified isolates were stored in cryogenic tubes. The bacterial strains screened for antagonistic activity toward plant fungal pathogen using dual culture technique refer to the method of Yu et al. (2011) and Duan et al. (2021).

### Identification of Antagonistic Strain

The morphological identification was conducted based on the methods of Zhao et al. (2018). Biochemical identification was based on the methods of Dong and Cai (2001) and Vos et al. (2011). The GEN III MicroPlate™ test panel identification was based on the methods of Bochner (1989).

Genomic DNA was extracted from the obtained isolates using the Bacteria Genomic DNA Extraction Kit (Tiangen Corporation Ltd, Beijing, China) according to the manufacturer’s instructions (Sambrook and Russel, 2001). Molecular identification was performed on strain XNRB-3 using 16S ribosomal RNA gene (16S rDNA), DNA gyrase subunit A (*gyrA*), DNA gyrase subunit B (*gyrB*), and RNA polymerase subunit B (*rpoB*) gene sequence analysis. The primers and annealing temperatures are shown in Supplementary Table 3. Polymerase chain reaction (PCR) amplification, product purification, and sequencing refer to the method of Duan et al. (2021).

Sequences of 16S rDNA, *gyrA*, *gyrB*, and *rpoB* were aligned using maximum likelihood (ML) methods were performed for the datasets using RAxML-HPC2 on XSEDE (8.2.12) on the CIPRES website^1^ (Stamatakis, 2014). Tree diagrams were created in FigureTree v1.4.3 and Adobe Illustrator CS6.

### Polymerase Chain Reaction Detection of Antibiotic Biosynthesis Genes

Genes related to the biosynthesis of lipopeptides, dipeptides and polyketides were detected by PCR using the primers listed in Supplementary Table 4. Amplification was performed with an Applied Biosystems 2720 Thermal Cycler (Applied Biosystems Inc., California, CA, United States). Following amplification, the PCR reaction mix (5 μL) was visualized by gel electrophoresis in a 1.5% agarose gel.

### Optimization of Fermentation Conditions

Seed culture of *B. licheniformis* XNRB-3 was prepared by inoculating a single colony into 100 mL of Luria-Bertani (LB) broth (tryptone 10 g, yeast extract 5 g, NaCl 10 g, pH 7.0) and incubated at 37°C overnight with agitation (180 rpm).

#### Single Factor Test

Batch fermentation was carried out in 250-mL Erlenmeyer flasks containing 100 mL of fermentation medium (glucose 22 g, tryptone 5.4 g, KH_2_PO_4_ 2.4 g, CaCl_2_ 0.25 g, MgSO_4_ 0.5 g, 1 L, pH 7.0) inoculated with 3–10% seed culture, and incubated at 37°C with agitation (180 rpm) for 72 h (Qiao et al., 2012). The OD_600_ absorbance value of the bacterial solution was measured at different incubation times and the serial dilution method was used to count the number of bacteria (Supplementary Figure 1a).

The variables used were carbon sources (sucrose, maltose, glucose, lactose, soluble starch), nitrogen sources [yeast extract, peptone, (NH_4_)_2_SO_4_, beef extract, NH_4_NO_3_, urea, and NH_4_Cl], inorganic salts (KH_2_PO_4_, CaCl_2_, MgSO_4_, NaCl, MnSO_4_, and KCl), and culture conditions (filling volume, speed, temperature, and pH) each at different levels, as shown in Supplementary Table 5. For the selection of inorganic salts, one inorganic salt is removed at a time, and its influence on the growth of strain XNRB-3 was investigated. According to the results of the inorganic salt removal test, three important inorganic salts were selected, and the orthogonal experiment was designed according to the orthogonal table to determine the best combination of inorganic salts. When the above fermentation conditions were optimized, except for the test single factor as a variable, the other conditions were unchanged. Each treatment contained three repetitions. The 24 h OD_600_ (diluted three times) and the antibacterial rate were determined and combined with the results of single factor experiment, orthogonal experiment (Orthogonality Experiment Assistant V3.1), Plackett–Burman, and Box–Behnken (Design-Expert 8.0.6) in response surface analysis (RSM) to optimize the composition and fermentation conditions of the *B. licheniformis* XNRB-3 sporulation shake flask fermentation medium (Anthony et al., 2009).

### Antagonistic Effects of Extracellular Metabolites on *Fusarium*

The cell-free culture filtrate (CFCF) of strain XNRB-3 was obtained according to the method of Abdelmoteleb et al. (2017). Bacteria were grown on the optimized liquid fermentation medium with constant shaking at 191 rpm for 24 h at 33 to obtain fermentation broth (FB), centrifugated at 10,000 rpm for 10 min at 4, and the supernatant (extracellular medium) was passed through a 0.22-μm Nylon66 microporous membrane to obtain the CFCF. The effect of strain XNRB-3 on *Fusarium* spore germination and hyphae refers to the method of Duan et al. (2021).

### Plant Growth Promoting Activities

The ability of strain XNRB-3 to promote plant growth was tested with reference to the method in Supplementary Table 6. The FB of *B. licheniformis* XNRB-3 was centrifuged at 13,400 *g* for 10 min. The supernatant (extracellular medium) was removed and frozen at 4 for the analysis of the amino acids. Phytohormone extraction and quantitation were conducted using gas chromatography-mass spectrometry/selected ion monitoring (GC-MS/SIM) (Shimadzu, Japan). The specific method is mentioned in Supplementary Table 6.

### Determination of Microorganisms Using Phlorizin as Carbon Source

#### Preparation of 0∼10 mmol L^–1^ Phlorizin Culture Medium

Chemical compounds of phlorizin, cinnamic acid (CA), ferulic acid (FA), benzoic acid (BA), and *p*-hydroxybenzoic acid (PHBA) were Sigma products (Sigma, St. Louis, MO, United States). The mineral salt medium (MSM) [K_2_HPO_4_ 5.8 g, (NH_4_)_2_SO_4_ 2.0 g, KH_2_PO_4_ 4.5 g, CaCl_2_ 0.02 g, MgCl_2_ 0.16 g, FeCl_3_ 0.0018 g, Na_2_MoO_4_⋅2H_2_O 0.0024 g, and MnCl_2_⋅2H_2_O 0.0015 g in 1 L, pH 7.0] containing phlorizin (0∼10 mmol L^–1^) as the sole carbon source was used to isolate degrading bacteria. Agar plates were prepared by adding agar (18 g L^–1^) to the MSM, and the MSM solution with phlorizin was used to determine the maximum absorption wavelength and standard curve, and the operation was protected from light as much as possible (Pant et al., 2013).

#### Tests of Microbial Growth and Phenolic Degradation

The absorbance value of MSM solution with phlorizin (10 mmol L^–1^) at each wavelength was measured, and it was determined that the maximum absorbance wavelength was 280 nm (Supplementary Table 7). The MSM solution with a total of 11 concentrations of 0-10 mmol L^–1^ were prepared. The MSM solution with 0 mmol L^–1^ phlorizin was used as the reference solution for blank calibration and to determine the OD_280_ absorbance value. The absorbance value is taken as the ordinate and the phlorizin concentration as the abscissa to form a standard curve (Supplementary Figure 4p).

The tests of microbial growth and phenolic acid degradation in MSM solution refer to the method of Wang et al. (2021), with some modifications. Strain XNRB-3 was inoculated into MSM with 0∼10 mmol L^–1^ phlorizin and cultured at 33°C in the dark to determine the maximum tolerance of phlorizin (Supplementary Figure 4n). Strain XNRB-3 was inoculated with 6 mL of liquid fermentation medium in a 15-mL plastic tube with a cover, followed by overnight incubation at 33°C. After centrifugation at 2,500 *g* for 5 min, the supernatant was discarded and the pellet was diluted to an OD_600_ value of 1.0 using ddH_2_O. Afterward, 0.1, 0.15, and 0.2 mL of the resuspended isolates were transferred to 10 mL of the MSM solution with phlorizin (3 mmol L^–1^) and incubated for 60 h in the dark at 33°C with shaking (191 rpm min^–1^). Then, 1 mL of the bacterial suspension was taken from each treatment, centrifuged at 13,000 *g* for 5 min, the supernatant was taken to measure the OD_280_ value, and it was converted to the concentration of phlorizin according to the standard curve. The degradation rate of phlorizin = (phlorizin concentration in uninoculated culture solution-phlorizin concentration in inoculated culture solution)/phlorizin concentration in uninoculated culture solution × 100%.

To test the isolate’s ability to utilize other phenolic acids, strain XNRB-3 was inoculated into the MSM solution with 0.5 g L^–1^ of CA, FA, BA, or PHBA, and their growth was tested according to the previously described method, where 0.2 mL of the suspension was transferred to 10 mL of MSM solution with 0.5 g L^–1^ of CA, FA, BA, or PHBA. After incubation for 12, 24, 36, 48, and 60 h at 33°C with shaking (190 rpm min^–1^), 1 mL of the bacterial suspension was taken from each treatment and was centrifuged at 13,000 g for 5 min. The pellet was resuspended with 1 mL ddH_2_O to detect OD_600_, and the supernatant was mixed with an equal volume of methanol. Then, 0.1 mL of the mixed solution was added to 1.9 mL of 50% methanol solution and was sterilized by filtering through 0.22-μm pore-size filter membranes prior to High Performance Liquid Chromatography (HPLC) analysis. The control used was MSM solution of phenolic acids but without bacteria, where all assays were performed in triplicate.

The concentrations of phenolic acids were detected based on peak areas using external standards using an UltiMate 3000 HPLC system (Dionex Corporation, Sunnyvale, CA, United States). All separations were performed using Symmetry^^®^^ C18 column (4.6 mm × 150 mm, 5.0 μm; Waters, Milford, MA, United States). Mobile phase solutions were 0.1% methanoic acid + 2% methanol (A) and acetonitrile (B). The gradient elution composition used was as follows: 0 min, 96% A plus 4% B 10 min; 10% A plus 90% B 16 min; 96% A plus 4% B 30 min; with a flow rate of 1.0 mL min^–1^, an injection volume of 10 μL, a column temperature of 20°C and Ultraviolet (UV) detection at 280 nm (Wang et al., 2021).

#### Soil Treatment With Microbes and Analysis of Phenolic Acid Degradation

The soil obtained from a 31-year-old apple orchard in Manzhuang Town, Taian, China (117.081039 longitude, 36.06682 latitude) was dried at 60°C for 6 h and was passed through a 0.84-mm sieve. Here, 30 μg g^–1^ of phlorizin, 10 μg g^–1^ of CA, 90 μg g^–1^ of BA, 100 μg g^–1^ of FA, and 20 μg g^–1^ of PHBA was added to the dried soil. The soil was dried again at 60°C for 2 h. Then, 200 mL with OD_600_ = 1.0 of bacterial suspension was added to 1 kg of soil; sterile water was used as control. Each treatment consisted of three replicates. After incubation for 3, 6, and 9 days at 25°C/20°C (16 h/8 h, light/dark), ddH_2_O was added to keep the soil moist. 60 g of soil was obtained from each pot for HPLC analysis (Zhang Z. Y. et al., 2010).

Extraction of phenolic acids from soil using the method of Yin et al. (2013). The HPLC analysis procedure followed that described by Xiang et al. (2021). An UltiMate 3000 HPLC system (Dionex, United States) was used for quantification with a Symmetry^^®^^ C18 column (4.6 mm × 250 mm, 5.0 μm; Waters, Milford, MA, United States), and a column temperature of 30°C. Mobile phase A was acetonitrile, and mobile phase B was water (adjusted to pH 2.8 with acetic acid). The flow rate was 1.0 mL min^–1^, the automatic injection volume was 10 μL, and the detection wavelength was 280 nm. The HPLC solvents were purchased from Burdick & Jackson Inc. (Muskegon, MI, United States). The retention time was used for qualitative analysis, and the peak area external standard method was used for quantification.

### Root Colonization Assay

A spectinomycin- and rifampin-resistant mutant of strain XNRB-3 (denoted XNRB-3^R^) was obtained by inoculation of strain XNRB-3 into the LB medium containing gradually increasing concentrations of spectinomycin and rifampin (20, 50, 75, 100, 150, 200, 250, and 300 mg mL^–1^; Supplementary Figure 4o) refer to the method of Yu et al. (2011). *Malus hupehensis* Rehd. seedlings were selected as the test material.

The ability of *B. licheniformis* XNRB-3 to colonize roots was determined according to the method of Sanei and Razavi (2011), with some modifications. The *M. hupehensis* Rehd. seedlings were carefully uprooted from the substrate, their roots thoroughly washed in tap water without intentional wounding, and dipped in a bacterial (XNRB-3^R^) suspension [1 × 10^8^ colony-forming units (CFU) mL^–1^] for 10 min. For the control treatment, plants were treated similarly except that the roots were dipped in 10 mM MgSO_4_.7H_2_O. Plants were then transplanted (one per pot) into pots (AC140: the outer diameter is 12.5 cm, the inner diameter is 11 cm, and the height is 9.5 cm) filled with an autoclaved (121°C, 1 h, twice on consecutive days) soil mixture (180 mL vermiculite first, then 540 mL of soil), and finally covered with 180 mL of sterile vermiculite. There were four replicated plants for each treatment in a randomized complete block design. The experiment was repeated three times. Plants were incubated under greenhouse conditions. The air temperature during the experiment fluctuated between 18 and 33°C. Plants were watered as needed. To determine colonization of root tissue by the bacteria, plants were uprooted delicately from the pots and the root systems were thoroughly washed under running tap water, dried with sterile filter paper, and cut into 1-cm-long pieces. For each plant, samples of 2 g of root pieces were surface-deinfested in 1% NaOCl for 3 min, washed three times in sterile distilled water, and ground in 10 mL of 10 mM MgSO_4_.7H_2_O using an autoclaved pestle and mortar. Serial dilutions of the macerates were plated onto LB agar (LB supplemented with spectinomycin and rifampin at 300 mg mL^–1^) and incubated at 37°C for 48 h. Then, bacterial colonies were counted, and the bacterial populations were expressed as CFU g ^–1^ of fresh root tissue.

### The Protective Effect of Strain XNRB-3 on Plant Roots

The protective function of strain XNRB-3 on the roots of *M. hupehensis* Rehd. seedlings was verified by Periodic Acid Schiff (PAS) staining according to the method of Shao et al. (2020) and Duan et al. (2021).

### Biological Control of the Strain XNRB-3

The ability of strain XNRB-3 to control ARD was investigated in a *Fusarium*−infested sterilized soil following the method described by Wu et al. (2019), with some modifications. Treatments included a sterilized soil inoculated with sterile distilled water as a negative control, a sterilized soil inoculated with *Fusarium* as a positive control, and sterilized soil inoculated with *Fusarium* and the strain XNRB-3. Each treatment included 15 *M. hupehensis* Rehd. seedlings. The spore suspension of *Fusarium* was first drenched into the sterilized soil, followed by a suspension of strain XNRB-3. The final concentration of *Fusarium* (10^5^ spores g^–1^) and strain XNRB-3 (10^8^ CFU g^–1^) was included in the growth substrates. Plant seedlings were then transplanted in the substrate trays (AC140), and then grown at 16 h light/8 h dark at 28°C. Plants were watered as required for plant growth and disease development. Each pot contained one *M. hupehensis* seedling, and all pots were arranged randomly with 15 replicates per treatment (Cachinero et al., 2002). The experiment was repeated three times. Disease severity was estimated over the course of 5 weeks starting 1 week after inoculation. The scoring criteria and calculation formula are presented in Supplementary Table 8.

The PDP was measured to estimate wilt severity and the ability of plants in different treatment groups to recover from the disease. To fulfill Koch’s postulates, isolates from discolored fibrous roots were also obtained from all dead plants at the end of the experiment. Isolation and identification of each organism were performed to the genus level. The growth substrate samples near to plant root rhizosphere were collected weekly after being transplanted and stored at –20°C. The population of *Fusarium* in the growth substrate samples were determined by real-time PCR.

### Separation and Identification of Extracellular Metabolites

#### Inhibition of Mycelial Growth by Cell-Free Culture Filtrate

The CFCF was used to assess influence of extracellular metabolites on *Fusarium* radial growth as described by Azabou et al. (2020), with some modifications. The bacterial culture was grown in a shaker incubator at 100 rpm for 72 h. The culture was then centrifuged at 10,000 rpm for 5 min at 4°C. The supernatant was collected and filtrated through 0.22-μm membrane filters. The CFCF was added to a warm PDA medium (55°C) to the final concentrations (from 5 to 75%). The PDA plates without culture filtrate were used as controls. Fungal mycelial plugs 5 mm in diameter were placed centrally in the amended media and incubated at 25°C until negative control growth covered the whole surface of the plate. The inhibition of the pathogen growth was estimated following the formula described by Erdogan and Benlioglu (2010): PI = (D – d)/D × 100%, where *PI*, inhibition of pathogen growth (%); *D*, diameter of pathogen growth in control plates (mm); and *d*, diameter of pathogen growth for the tests (mm).

#### Identification of Extracellular Metabolites by Gas Chromatography-Mass Spectrometry

The fermentation supernatant of strain XNRB-3 was extracted with ethyl acetate and then concentrated under reduced pressure with Rotary Evaporator N-1300D-WB (Tokyo, Japan) to obtain a crude extract. A small amount of methanol was added to dissolve the extracts and then passed through a Nylon66 0.22-μm filter membrane. The compounds in the extract were identified by GC-MS followed by an NIST17 database search. The GC-MS conditions were as described by Duan et al. (2021). The peak area normalization method was used to calculate the relative content of each component.

#### Verification of Synthetic Compounds Against Plant Fungal Pathogen

Among the identified extracellular metabolites, 16 standard compounds were purchased from the reagent company (Supplementary Table 9). The antifungal activity of the standard compounds was assessed using the I-plate system described by Yuan et al. (2012). The I-plates combined with the addition of methanol or distilled water were used as the control. The colony diameter of the plant fungal pathogen was recorded after incubating for 7 days. The experiment was repeated three times.

#### Plant Growth Promotion Activities of Synthetic Compounds

The growth promotion activities of the 16 compounds were measured by the modified method as described by Duan et al. (2021). Briefly, the synthetic compounds were diluted separately in ethanol, and 20 μL of the resulting suspension was applied to a sterile filter paper disk on the other side of the I-plate. A total of 10, 100, 500, and 1,000 μg doses of each synthetic compound were tested. Each treatment was repeated three times. The fresh weight of the *Arabidopsis thaliana* Col-0 seedlings was measured after 10 days.

### Carrier Characteristics

In the present study, 15 carriers purchased from different Chinese commercial enterprises through Taobao were used as formulation carriers for strain XNRB-3. The selected properties of these carrier candidates are listed in Supplementary Table 10. The carriers were dried to a moisture content of 5% in an oven (Shandong, China) at 80°C for 24 h, finely ground in a hammer mill to pass through a 1-mm screen, and stored at room temperature for further study.

The effect of the carrier as a substrate on the survival of strain XNRB-3 was evaluated using plastic bottles equipped with a 0.22-μm filter membrane in the cap to allow for air exchange, which was in reference to the method of Wei et al. (2015), with some modifications. Then, 20 mL of fermentation broth of strain XNRB-3 was mixed with 100 g of each carrier (sterilization at 115°C for 30 min). Carriers treated with 20 mL of aseptic fermentation broth were used as the controls. Each treatment included three replications (three bottles). In addition, these plastic bottles were covered with black plastic bags to avoid the influence of light on the survival of strain XNRB-3. The bottles were stored at 25°C and periodically sampled at 0, 10, 20, 30, 60, 90, 120, and 180 days post-inoculation. The population of strain XNRB-3 in each carrier at each sampling time point was determined by the plate count method using LB agar.

Four key factors including inoculation amount, pH, temperature, and rotating speed were selected to carry out the Box–Behnken test design with four factors and three levels. When the above fermentation conditions were optimized, except for the test single factor as a variable, the other conditions were unchanged, and the population number of strain XNRB-3 was determined for 180 d. Using Box–Behnken (Design-Expert 8.0.6) in the RSM to optimize the solid fermentation conditions of strain XNRB-3 (Anthony et al., 2009).

### Pot Experiment

The BIO product was produced by Chuangdi Microbial Resources Co., Ltd. (Dezhou, China). The mixture of cow manure compost and wheat straw (1:2, w/w) was aerobically fermented with strain XNRB-3 for 6 days at 40°C, and the bacterial density was 5.0 × 10^9^ CFU g^–1^. All operations are carried out under optimized fermentation conditions. The content of organic matter was 46.21%, total nitrogen was 2.36%, P_2_O_5_ was 1.49%, and K_2_O was 3.03%.

In May 2017, the pot experiment was conducted at the National Apple Engineering Experiment Center of the Horticultural Science and Engineering College of Shandong Agricultural University and the State Key Laboratory of Crop Biology (117.156540 longitude, 36.164443 latitude). The collection and treatment of the experimental soil was conducted as described by Duan et al. (2021). The physicochemical properties of the tested soil were presented in Supplementary Table 11. There were four treatments: (1) 31-year-old orchard soil (CK1), (2) 31-year-old orchard soil fumigated with methyl bromide (CK2), (3) bacterial fertilizer carrier treatment (T1), and (4) XNRB-3 bacterial fertilizer treatment (T2). The addition amount of strain XNRB-3 and fertilizer carrier accounts for about 1% of the weight of the soil. The plants received unified watering and manure management and sample collection and management according to that of Duan et al. (2021).

### Field Experimental Trials

To test the potential of strain XNRB-3 bacterial fertilizer to control ARD under field conditions, the field test was carried out in Wangtou Village, Laizhou City (Shandong China, Long: 119.814701, Lat: 37.095159). Physicochemical properties of the tested soil are presented in Supplementary Table 11. After the apple orchard was rebuilt, severe ARD occurred, the growth of fruit trees was weak, and the survival rate was less than 50%. In March 2020, 28-year-old trees were removed from the orchard, and the replanted orchard was simultaneously established. The apple seedlings used in the experiment were 2-year-old grafted seedlings. The rootstock and spike combination was from Yanfu 3/T337. The grafted seedlings had a stem thickness of about 10 mm and a fixed stem of 1.4 m. They were purchased from Laizhou Nature Horticultural Technology Co., Ltd. (Shandong, China). The row spacing of the plants is 1.5 m × 4 m, and the tree shape was pruned to a spindle shape. The production of strain XNRB-3 bacterial fertilizer was as described above.

The experiment consisted of four treatments: 28-year-old orchard soil (CK1), 28-year-old orchard soil fumigated with methyl bromide (CK2), bacterial fertilizer carrier treatment (T1), and XNRB-3 bacterial fertilizer treatment (T2). A planting hole of 80 cm^3^ was dug according to the row spacing, and the bacterial manure carrier and XNRB-3 bacterial manure were mixed with soil and backfill. The amount of application for each young tree was controlled at 1 kg, and 20 trees were treated for each. A new application was carried out at the beginning of the second spring. All indexes were measured on 15 July and 20 October in 2020 and 2021. The sample collection and management were conducted in accordance with that done by Sheng et al. (2020).

### Measurement Indices

#### Physical and Chemical Properties of Soil

The physical and chemical properties and nutritional characteristics of the soil were measured as described by Xiang et al. (2021) and Zhang et al. (2021). The nitrogen in the soil was determined by the Kjeldahl method (Bremner, 1960), and the pH and electrolytic conductivity were measured using a sample to water ratio of 1:2.5 (w/v) with a PHS-3E digital pH meter (LEICI, Shanghai, China) and DDSJ-318 conductivity meter (Lei Magnetic, Shanghai, China). The soil particle size distribution (the percentage of clay, silt, and sand) was determined by the hydrometer method (Avery, 1973).

#### Microbial Culture Methods

The populations of soil microbes (bacteria, fungi, and actinomycetes) were assessed using the dilution method of plate counting as described by Duan et al. (2021).

#### Biomass and Related Parameters

The biomass (height, new shoot growth, and ground diameter) was measured as described by Duan et al. (2021). The plant root analysis adopts the Microtek ScanMaker i800 Plus scanner (Shanghai Zhongjing Technology Co., Ltd., Shanghai, China) and OXY-LAB oxygen electrode system (Hansatech Ltd., Hansatech, United Kingdom) in reference to the method used by Gao et al. (2010) and Duan et al. (2021).

#### Enzyme Activity

Superoxide dismutase (SOD), peroxidase (POD), catalase (CAT) activity, and malondialdehyde (MDA) content were determined as described in Li et al. (2015). Soil urease, acid phosphatase (ACP), catalase, and sucrase activities were assayed as described in Liu J. et al. (2018).

#### DNA Extraction and Real-Time Quantitative Analysis

Sieved fresh soil (5.0 g) was used for DNA extraction with the DNeasy PowerMax Soil Kit (Qiagen, Hilden, Germany). The specific steps refer to the method of Duan et al. (2021). The concentration of plasmid DNA was measured and converted to copy the concentration using the following equation as described by Whelan et al. (2003): DNA (copy) = [6.02 × 10^23^ (copies moL^–1^) × DNA amount (g)]/[DNA length (bp) × 660 (g moL^–1^ bp^–1^)]. The primers and annealing temperatures are presented in Supplementary Table 3. Sterile water was used as a negative control to replace the template. All real-time PCR reactions were done in technical triplicates such that each treatment was analyzed nine times.

#### Terminal-Restriction Fragment Length Polymorphism Analysis

The DNA was amplified using the universal primers 27F-FAM/1492R and ITS1F-FAM/ITS4R (Supplementary Table 3) that target the bacterial of the 16S rRNA gene and the fungal ITS region between the 18S and 28S rRNA regions, respectively. The specific steps refer to the method of Quéric and Soltwedel (2012), Xu et al. (2019), and Duan et al. (2021). The purified PCR product (500 ng) was digested by the restriction endonuclease *Msp*I (Takara Shuzo Co., Kyoto, Japan) for 16S rRNA gene amplicons and *Hin*fI (Takara Shuzo Co., Kyoto, Japan) for ITS amplicons in two separate reactions according to their respective protocols and sequenced by Sangon Biotech Co., Ltd. (Shanghai, China) (Zhang et al., 2018). The Terminal-Restriction Fragment Length Polymorphism (T-RFLP) data analysis was conducted in reference to Duan et al. (2021).

#### Biolog Plate Analysis

Biolog-ECO plates (Biolog Inc., Hayward, CA, United States) and a plate reader (Multiskan MK3 ELISA) were used to determine the community-level physiological profiles. The assessment method was referenced from previous studies (Classen et al., 2003; Ge et al., 2018), with some modifications. Briefly, 3 g of incubated soil was mixed in 27 mL of a sterile NaCl solution (0.85%) and then oscillated at 220 rpm for 30 min. After the suspensions settled for 10 min, 3 mL aliquots of the suspensions were added to 27 mL of a sterile NaCl solution (0.85%) as tenfold serial dilutions until a final 1:1000 dilution was reached; 150 μL of the supernatant was then added to each well of the Biolog-ECO plates. Microplates were incubated at 25°C and 85% ± 5% humidity in a dark room. The rate of utilization was indicated by the reduction of tetrazolium violet, a redox indicator dye, which changes from colorless to purple. Data were recorded at 590 nm every 24 h up to 168 h. The OD values at the 96 h incubation were used for subsequent statistical analyses (Dang et al., 2015). All treatments had three triplicates.

During data treatment, OD readings from the control wells were subtracted from the OD of the treatment wells. Wells with negative OD well responses were coded as zeroes. Average well color development (AWCD) and average absorbance of each category were calculated in line with the recommendations of Garland (1996). Indices of Shannon-Wiener diversity index, Shannon evenness index, McIntosh index, and Simpson diversity index were used to evaluate the richness and dominance of species in the soil microbial community (Dang et al., 2015). According to the method described by Ge et al. (2018), PCA was used to analyze the data of Biolog-ECO microplates.

(1) AWCD = [Σ(Ci - R)]/n. *Ci* is the absorbance value of each reaction well at 590 nm, *R* is the absorbance value of the control well, and *n* is the number of wells.
(2) Shannon-Wiener diversity index (*H’*) reflects the species richness. H’ = -ΣPi. ln(Pi). The *Pi* represents the ratio of the absorbance value in the ith (1 to 31) well to the total absorbance values of all wells.
(3) Shannon evenness index (*E*): E = H’/Hmax = H’/lnS, where *S* represents the total number of utilized carbon sources (31 carbon sources), the number of wells that vary in color.
(4) Simpson diversity index (*D*) reflects the most common species of the community, and was often used to assess the dominance degree of microbial community. D = 1-Σ(Pi)^2^
(5) McIntosh index was used to measure the homogeneity degree of the community.

U = √Σni^2.^ ni was the relative absorbance of the ith hole (*Ci-R*).

### Statistical Analysis

All statistical analyses were performed with the IBM SPSS 20.0 (IBM SPSS Statistics, IBM Corporation, Armonk, NY, United States). Different lowercase letters represent significant differences between treatments (one-way ANOVA, *p* < 0.05) according to Duncan’s multiple range test. The figures were plotted with Microsoft Excel 2013 (Microsoft Corporation, Redmond, WA, United States) and GraphPad Prism 7.0 (GraphPad Software, Inc., San Diego, CA, United States). The R statistical platform (v.4.1.1) was used for principal coordinates (PCoA) and cluster analysis (Duan et al., 2021).

## Results

### Isolation and Identification of Bacteria for Biocontrol Activity

**FIGURE 1:**
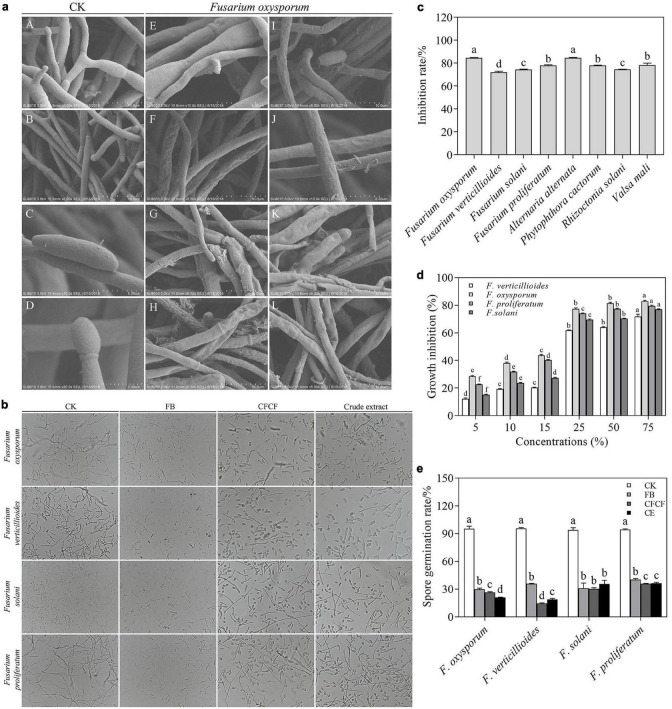
Antifungal activity of strain XNRB-3 against plant fungal pathogen. **(a)** The mycelia and spore morphology of *Fusarium oxysporum* under the scanning electron microscope. (A–D) Was the normal mycelium and spore, (E–L) was the mycelium treated with fermentation broth. **(b)** Effects of different treatments on spore germination of *Fusarium*. CK, *Fusarium* spore suspension was mixed with sterile water at 1:1; FB, *Fusarium* spore suspension was mixed with fermentation broth at 1:1; CFCF, *Fusarium* spore suspension was mixed with cell-free culture filtrate at 1:1. Crude extract (CE), *Fusarium* spore suspension was mixed with extracellular metabolites at 1:1. **(c)** Inhibition rate of strain XNRB-3 against plant fungal pathogen. **(d)** Effects of different concentrations of CFCF on the *Fusarium* mycelial growth. **(e)** Germination rate of *Fusarium* spores after different treatments. Values in columns followed by the same letter are not significantly different according to Duncan test at *p* < 0.05. Values are mean ± SD (*n* = 3).

The bacterium XNRB-3 was isolated from the root system of a healthy apple tree in a replanted orchard in Jincheng City, Shanxi Province. This bacterium had the strongest inhibitory effect on plant pathogenic fungi, with an inhibition rate of more than 70%. *F. oxysporum* and *Alternaria alternata* had the strongest inhibition rates, reaching 84.52 and 84.59%, respectively (Figure 1c).

Strain XNRB-3 was cultured on an LB liquid medium at 37°C for 24 h and formed biofilms. According to *Bergey’s Manual of Systematic Bacteriology* (2nd edition) and the *Common Bacterial Identification Manual*, strain XNRB-3 matched the physiological and biochemical properties and morphological characteristics of *B. licheniformis* (Supplementary Figure 2 and Supplementary Table 12). Tests of the carbon source utilization and chemical sensitivity revealed that strain XNRB-3 can utilize 32 carbon sources and was sensitive to pH 6, 1% NaCl, 4% NaCl, and 8% NaCl conditions. The values of probability (PROB), similarity (SIM), and distance (DIST) were 0.689, 0.689, and 4.698, respectively, and strain XNRB-3 belonged to *B. licheniformis*, which was identified based on carbon utilization (Supplementary Tables 13, 14).

Approximately 555–938 bases were sequenced for *rpoB* and *gyrA*, 1,455 bases for 16S rDNA, and 1,136 bases for *gyrB*. The ML analysis of identities based on the four gene sequence alignments revealed that strain XNRB-3 had the highest homology with *B. licheniformis* (Supplementary Figure 3). In sum, strain XNRB-3 was identified as *B. licheniformis*.

### Plant Growth-Promoting Activities

The endophytic bacterium XNRB-3 possessed multiple PGP properties such as phosphate and potassium solubilization; nitrogen fixation; IAA, GA, and ABA production; ACC deaminase, ammonia, and amylase production; siderophore, cell wall-degrading enzyme (cellulase, pectinase, β1,3-glucanase, chitosanase, and protease), and antifungal activity against phytopathogens (Supplementary Tables 6, 15 and Supplementary Figures 4a–m).

An HPCE method was used to identify 20 Phenyl isothiocyanate (PITC)-derivatized amino acids, 17 of which were separated (Supplementary Table 16), including aspartic acid (Asp), threonine (Thr), serine (Ser), glutamate (Glu), proline (Pro), glycine (Gly), alanine (Ala), cystine (Cys), valine (Val), methionine (Met), isoleucine (Ile), leucine (Leu), tyrosine (Tyr), phenylalanine (Phe), histidine (His), lysine (Lys), and arginine (Arg).

### Identification of Antibiotic Biosynthesis Genes

To determine the mechanisms underlying the effects of strain XNRB-3, PCR was used to screen strain XNRB-3 for genes involved in the biosynthesis of antibiotics. Amplicons of the expected sizes detected included *yndJ* (involved in biosynthesis of Yndj protein), *qk* (involved in subtilisin synthesis), *bamC* (involved in bacillomycin synthesis), *ituD* (involved in iturin A synthesis), *fen* and *fenD* (involved in fengycin synthesis), and *srf* and *srfAB* (involved in surfactin synthesis) (Supplementary Figure 5).

### Optimization of Liquid Fermentation Conditions

The results of single-factor tests showed that the main carbon source affecting the growth of strain XNRB-3 was sucrose, the main nitrogen source was beef extract, and the inorganic salts were MgSO_4_, KH_2_PO_4_, and KCl (Supplementary Figures 1d,g,h). According to the orthogonal test results (Supplementary Table 17), the organic salt that had the strongest effect on the growth of strain XNRB-3 was MgSO_4_, followed by KH_2_PO_4_ and KCl. According to the K value, the optimal combination was F4I3E3 (MgSO_4_ 1.0 g L^–1^, K_2_HPO_4_ 1.5 g L^–1^, and KCl 1.0 g L^–1^). According to the results of the single-factor experiment (Supplementary Figure 1), the Plackett–Burman experiment was performed using the experimental factors and levels in Supplementary Table 18, and Minitab 17 Software was used for multiple regression analysis. The optimal equation with OD_600_ as the response value was OD_600_ = 0.4472 + 0.004009A – 0.004149B – 0.01018C – 0.01204D + 0.00649E – 0.01527F + 0.00465G + 0.001711H + 0.000272I. According to the size of the *P*-value, the key factor affecting OD_600_ was A (temperature), followed by B (rotating speed), G (sucrose), H (beef extract), F (MgSO_4_), I (KH_2_PO_4_), D (pH), C (liquid volume), and E (KCl) (Supplementary Tables 19, 20). The effect of A–H was highly significant (*p* < 0.01), the effect of F was significant (*p* < 0.05), and the effect of I–E was not significant. A Box–Behnken test was conducted with four factors (sucrose, beef extract, temperature, and rotating speed) and three levels (Supplementary Table 21). Design Expert 8.0.6 software was used to perform regression analysis and obtain the multiple quadratic regression equations: *Y* = 22.054 – 0.1142A – 0.0875B + 0.4358C – 0.5942D – 0.1175AB + 1.6175AC + 0.1075AD – 0.4825BC – 0.0975BD – 0.6425CD – 0.8941A^2^ – 0.4016B^2^ – 1.7641C^2^ – 2.1366D^2^, where *Y* is the inhibition zone diameter (mm), *A* is sucrose, *B* is beef extract, *C* is temperature, and *D* is rotating speed. An analysis of variance and a significance test of the regression model were conducted (Supplementary Table 22 and Table 1), the *F*-value of the regression model was 94.09 (*p* < 0.0001), indicating that the regression model was robust. The coefficient of determination of the model was *R*^2^ = 0.9895 and *R*^2^_Adj_ = 0.9790, indicating a good fit. Therefore, the regression model could be used to analyze and predict the abundance of the strain XNRB-3. In the regression model, the primary term C, D and the secondary term AC, CD significantly affected the diameter of the inhibition zone (*p* < 0.0001); the significance of A, B, C, and D was the same based on the results of the Plackett–Burman test.

**TABLE 1:** ANOVA for response surface quadratic model.

Source	Degree of freedom	Sum of squares	Mean squares	*F* value	*P*-value (Prob > F)	Significance
Model	14	62.98152943	4.498680673	94.09309895	<0.0001	**
A-Sucrose	1	0.156408333	0.156408333	3.271391293	0.092019216	
B-Beef extract	1	0.091875	0.091875	1.921630828	0.187359213	
C-Temperature	1	2.279408333	2.279408333	47.67544296	<0.0001	**
D-Rotating speed	1	4.236408333	4.236408333	88.60748683	<0.0001	**
AB	1	0.055225	0.055225	1.155070068	0.300664777	
AC	1	10.465225	10.465225	218.8876079	<0.0001	**
AD	1	0.046225	0.046225	0.96682868	0.342160859	
BC	1	0.931225	0.931225	19.47723176	0.000589533	*
BD	1	0.038025	0.038025	0.795319861	0.387567486	
CD	1	1.651225	1.651225	34.53654273	<0.0001	**
A^2^	1	5.185200045	5.185200045	108.4521388	<0.0001	**
B^2^	1	1.046070315	1.046070315	21.87930303	0.000356012	*
C^2^	1	20.18588113	20.18588113	422.2020295	<0.0001	**
D^2^	1	29.61073518	29.61073518	619.3295407	<0.0001	**
Residual	14	0.669353333	0.047810952			
Lack of Fit	10	0.472033333	0.047203333	0.956888979	0.568501936	
Pure Error	4	0.19732	0.04933			
Cor Total	28	63.65088276				

*R^2^ = 98.95%, R^2^_Adj_ = 97.90%.*

* and ** Represented significant difference at p < 0.05 and p < 0.01, respectively.

The interactions between temperature and sucrose, temperature and beef extract, and rotating speed and beef extract were the most significant; the interactions between rotating speed and sucrose, temperature and rotating speed, and the sucrose and beef extract on the diameter of the inhibition zone were relatively weak (Supplementary Figure 6). The optimal fermentation conditions for strain XNRB-3 predicted by the regression model were sucrose 21.03 g, beef extract 8.54 g, temperature 32.73°C, and rotating speed 191.46 rpm. The maximum theoretical value of the predicted inhibition zone diameter was 22.17 mm. To further verify the predicted value, three parallel experiments were performed using the optimized fermentation conditions. The diameter of the inhibition zone was 22.03 ± 0.24 mm, and the error from the theoretically predicted value (22.17 mm) was only 0.63%, indicating that the predicted value was a good fit with the measured value. Thus, the optimized model was reliable.

In summation, the optimal fermentation conditions for strain XNRB-3 were sucrose 21.03 g, beef extract 8.54 g, MgSO_4_ 1.0 g, K_2_HPO_4_ 1.5 g, KCl 1.0, 1 L, pH 7.5, temperature 32.73°C, rotating speed 191.46 rpm, inoculation amount 5%, and filling volume 40%. Under optimal conditions, the OD_600_ values (after dilution) of the strain XNRB-3 solution at different incubation times were measured and the growth curves were drawn (Supplementary Figure 1b).

### Optimization of Solid Fermentation Conditions

**FIGURE 2:**
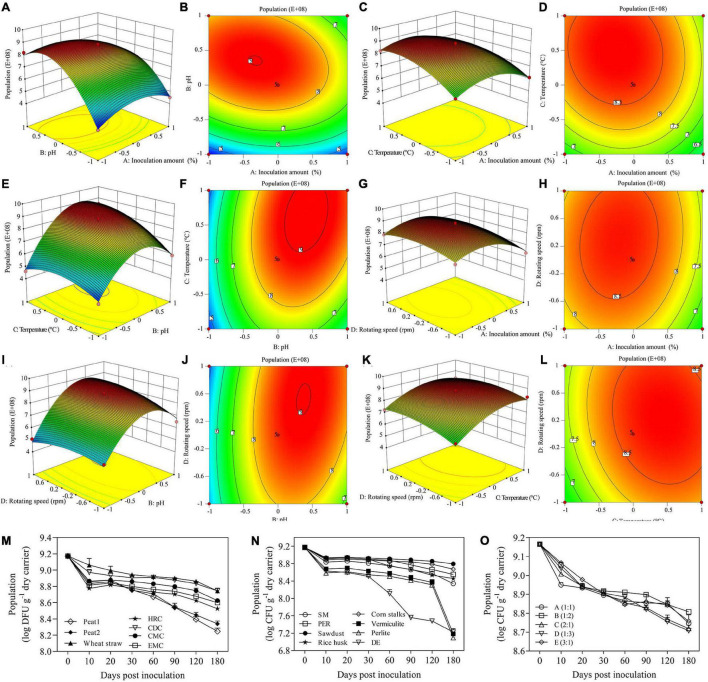
Response surface analysis three-dimensional and contour plot for population **(A–L)**. Shelf life of *B. licheniformis* XNRB-3 in different carrier formulations **(M,N)**. Plate counts on V8-salt medium from Earthworm manure compost (EMC), *Pleurotus eryngii* residue (PER), Chicken manure compost (CMC), Herb residue compost (HRC), cow dung compost (CDC), Diatomite earths (DE), Soybean meal(SM), peat1, and peat2 carriers inoculated with aseptic fermentation broth or *B. licheniformis* XNRB-3 were determined on different days post-inoculation. **(O)** Shelf life of *B. licheniformis* XNRB-3 under different carrier ratios. A: CDC: wheat straw = 1:1, B: CDC: wheat straw = 1:2, C: CDC: wheat straw = 2:1, D: CDC: wheat straw = 1:3, E: CDC: wheat straw = 3:1.

A long and stable shelf life is one of the most important commercial characteristics of biocontrol products. Therefore, the population dynamics (an index of shelf life) of strain XNRB-3 using different candidate carriers were characterized in the LB medium. At the end of the storage period, the viable numbers of strain XNRB-3 in cow dung compost (CDC) and wheat straw were greater than 8.78 and 8.74 log CFU g^–1^ dry carrier, respectively. CDC: wheat straw maintained the largest populations of strain XNRB-3 at a ratio of 1:2 (Figures 2M–O). Design Expert 8.0.6 software was used to perform regression analysis of the test data of Supplementary Tables 23, 24, and the multiple quadratic regression equation obtained was as follows: Y = 8.716-0.43A + 1.2608B + 0.6767C + 0.2692D – 0.52AB – 0.155AC + 0.145AD + 0.6125BC + 0.465BD – 0.1475CD – 0.9405A^2^ – 2.0218B^2^ – 0.7355C^2^ – 0.4243D^2^, where *Y* is population (E + 08), *A* is the inoculation amount; *B* is pH, *C* is temperature, and *D* is rotating speed.

The regression model was further analyzed by variance analysis and significance tests (Table 2). The *F* value of the regression model was 102.13 (*p* < 0.0001), indicating that the regression model was robust. The coefficient of determination of the model was *R*^2^ = 0.9903 and *R*^2^_Adj_ = 0.9806, indicating a good fit. Therefore, the regression model could be used to analyze and predict the abundance of *B. licheniformis* XNRB-3. In the regression model, the effects of the first term A, B, C and the second term BC on the diameter of the inhibition zone were significant (*p* < 0.0001). The interactions between pH and inoculation amount, temperature and pH, and rotating speed and pH had the most significant effect on the population of strain XNRB-3 (Figures 2A–L). The optimal solid fermentation conditions for *B. licheniformis* XNRB-3 were predicted by the regression model: inoculation amount 20.25%, pH 8.25, temperature 39.23°C, and rotating speed 199.75 rpm. The predicted maximum theoretical value of the population of strain XNRB-3 was 8.91E + 08. To verify the predicted value, three parallel experiments were performed using the optimized fermentation conditions. The diameter of the inhibition zone was (22.03 ± 0.24) mm and the error from the theoretically predicted value (8.91E + 08) was only 0.86%, indicating that there was a good fit between the predicted value and the measured value and that the optimized model was robust.

**TABLE 2:** ANOVA for response surface quadratic model.

Source	Degree of freedom	Sum of squares	Mean squares	*F* value	*P*-value (Prob > F)	Significance
Model	60.51220155	14	4.322300111	102.1311598	<0.0001	**
A-Inoculation amount	2.2188	1	2.2188	52.4277842	<0.0001	**
B-pH	19.07640833	1	19.07640833	450.7543805	<0.0001	**
C-Temperature	5.494533333	1	5.494533333	129.8297313	<0.0001	**
D-Rotating speed	0.869408333	1	0.869408333	20.54315508	0.0005	*
AB	1.0816	1	1.0816	25.55700892	0.0002	*
AC	0.0961	1	0.0961	2.270736462	0.1541	
AD	0.0841	1	0.0841	1.987189765	0.1805	
BC	1.500625	1	1.500625	35.45810513	<0.0001	**
BD	0.8649	1	0.8649	20.43662816	0.0005	*
CD	0.087025	1	0.087025	2.056304273	0.1735	
A^∧^2	5.737558378	1	5.737558378	135.5721437	<0.0001	**
B^∧^2	26.51333878	1	26.51333878	626.4808023	<0.0001	**
C^∧^2	3.508931351	1	3.508931351	82.91215777	<0.0001	**
D^∧^2	1.167490135	1	1.167490135	27.58649759	0.0001	*
Residual	0.592495	14	0.042321071			
Lack of Fit	0.490975	10	0.0490975	1.934495666	0.2745	
Pure Error	0.10152	4	0.02538			
Cor Total	61.10469655	28				

*R^2^ = 99.03%, R^2^_Adj_ = 98.06%.*

* and ** Represented significant difference at p < 0.05 and p < 0.01, respectively.

In sum, the optimal solid fermentation component of *B. licheniformis* XNRB-3 was cow dung: wheat straw = 1:2, and the optimal conditions were inoculation amount 20.25%, pH 8.25, temperature 39.23°C, and rotating speed 199.75 rpm.

### Determination of Antifungal Activity

Microscopic observations of hyphal and spore morphology revealed that the control *Fusarium* mycelium was uniform in thickness and slender with fewer branches, spore structure was complete, and growth was strong (Figure 1aA–D). The mycelia treated with the CFCF were irregularly reticulated, uneven in thickness, shrunken (Figure 1aG–L), thinned (Figure 1aI,K), the cells had ruptured, and cell contents had overflowed (Figure 1aG,H,I,K), the spore cell wall was broken and deformed (Figure 1aE,H,I,K). Furthermore, FB, CFCF, and crude extract significantly inhibited the spore germination of *Fusarium* spores, and the spore germination rate decreased by more than 60% (Figures 1b,e). The antifungal metabolites produced by strain XNRB-3 were extracted from 3-day-old CFCF. As the concentration of extracellular metabolites increased, the inhibitory effect on the growth of *Fusarium* became more pronounced. At higher concentrations (25, 50, and 75%), the inhibition rate reached more than 60% (Figure 1d).

### Identification of Antifungal Compounds

**FIGURE 3:**
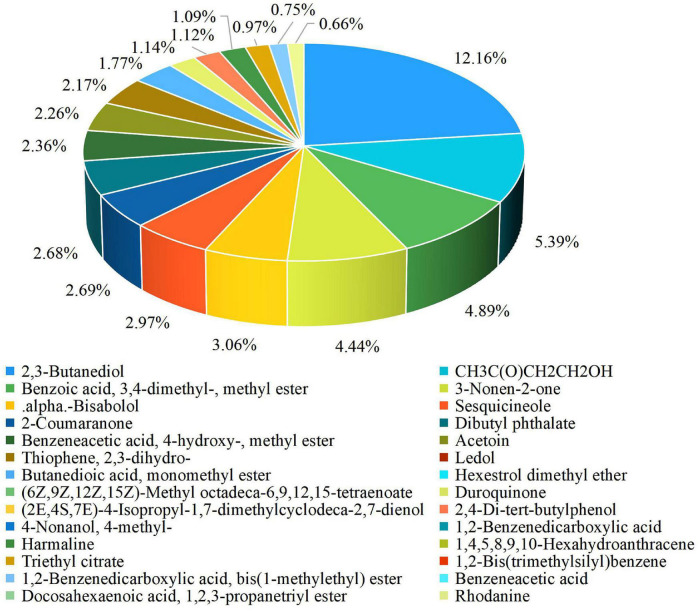
GC-MS identification result of extracellular metabolites.

**FIGURE 4:**
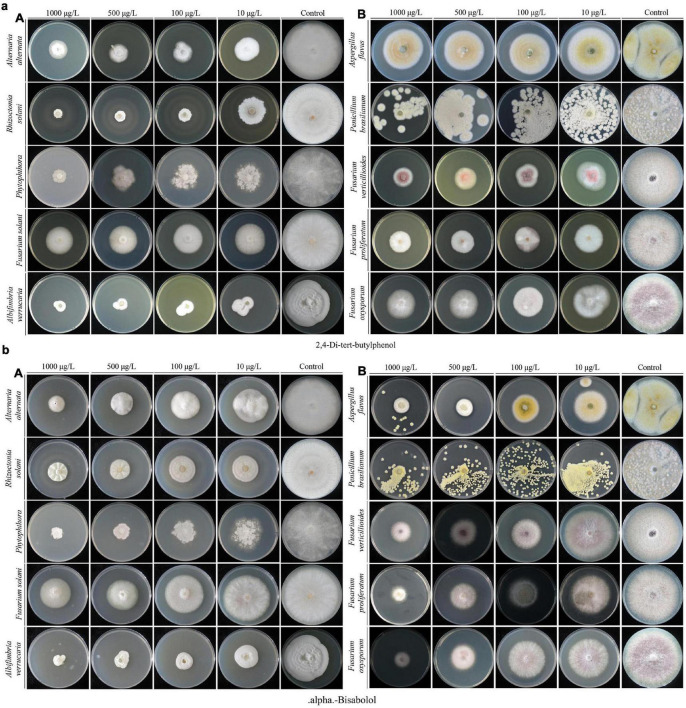
Growth inhibition of plant fungal pathogens on the PDA medium after treated with different gradient 2,4-Di-tert-butylphenol **(a)** and alpha-Bisabolol **(b)**.

**FIGURE 5:**
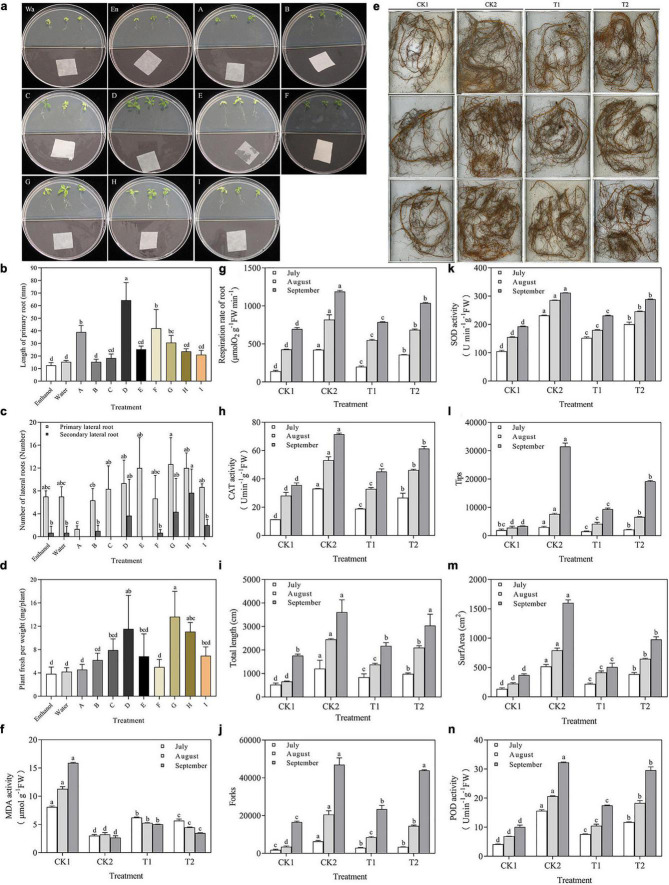
**(A)** Growth promotion of *Arabidopsis thaliana* Col-0 with exposure to pure compounds. **(a)** Wa, Water, En, Enthanol, (A) 2,3-Butanediol (100 μg), (B) 1,2-Benzenedicarboxylic acid, bis(1-methylethyl) ester (100 μg), (C) 2,4-Di-tert-butylphenol (500 μg), (D) Butanedioic acid, monomethyl ester (500 μg), (E) alpha-Bisabolol (500 μg), (F) Acetoin (500 μg), (G) Dibutyl phthalate (500 μg), (H) 3-Nonen-2-one (500 μg), (I) Benzoic acid, 3,4- dimethyl-, methyl ester (100 μg). **(b)** Plant fresh per weight, **(c)** length of primary root, **(d)** Number of lateral roots. **(e)** Effect of different treatments on the root architecture of *Malus hupehensis* Rehd. seedlings. A Root system scan obtained by Microtek ScanMaker i800 Plus. **(f–n)** Physiological indicators of root system, including MDA activity, root respiration rate, CAT activity, total root length, the number of root bifurcation (forks), SOD activity, root tips, root surface area, and POD activity. Values in columns followed by the same letter are not significantly different according to Duncan test at *p* < 0.05. Values are mean ± SD (*n* = 3).

The results of the experiment conducted for the present study showed that a 25% concentration of strain XNRB-3 CFCF showed high activity against fungal pathogens. After GC-MS chromatographic detection and analysis, the main antibacterial substances with Area% > 0.62 and retention index RI > 700 were selected (Supplementary Figure 7 and Supplementary Table 25). Most substances identified from the components were organic acids and esters, alcohols, ketones, alkanes, and phenols (Figure 3). Among the 42 identified compounds, 16 pure compounds were purchased for individual testing of antifungal properties (Supplementary Figure 8). The nine pure compounds showed antifungal activity against pathogenic fungi (Supplementary Table 26). Among them, 2,4-di-tert-butylphenol and alpha-bisabolol had the strongest inhibitory effect on plant pathogenic fungi, especially *Fusarium*. At a concentration of 1000 μg L^–1^, the inhibition rate was higher than 50% (Figures 4a,b). Of the nine pure compounds, the fresh weight and root growth of Arabidopsis plants compared with the control treatment (water and ethanol) were significantly enhanced by butanedioic acid, monomethyl ester (500 μg), and dibutyl phthalate (500 μg) (Figures 5a–d). Compared with ethanol treatment, the fresh weight, root length, number of primary roots, and number of secondary roots of Arabidopsis seedlings were increased by approximately 2.56, 1.42, 0.91, and 5.5 times, respectively, when treated with dibutyl phthalate; by 2.01, 4.08, 0.33, and 4.5 times, respectively, when treated with butanedioic acid and monomethyl ester; and by 1.89, 0.89, 0.71, and 10.5 times, respectively, when treated with 3-nonen-2-one. The above findings indicate that the main antifungal compounds produced by strain XNRB-3 play important roles in disease control and growth.

### Phlorizin Degradation Ability of Strain XNRB-3

The phlorizin utilization efficiency by strain XNRB-3 in the MSM solution was high (Supplementary Figure 4q). After culture for 60 h, the degradation rate was 60.75, 64.79, and 68.83% when the amount of inoculum added was 1, 1.5, 2%, respectively. The ability of strain XNRB-3 to degrade phlorizin increased as the amount of inoculum added increased. Strain XNRB-3 could effectively utilize CA, BA, FA, and PHBA (Supplementary Table 27), and the degradation rates ranged from 45.65% to 69.20%. Strain XNRB-3 could also efficiently degrade phlorizin, CA, BA, FA, and PHBA in soil (Table 3). The content of phlorizin in the soil decreased to 2.9292 μg g^–1^ at 9 days after inoculation. The content of BA and PHBA in the soil decreased to 18.4953 and 5.2882 μg g^–1^, respectively. The FA and CA concentrations in the soil decreased to 13.3669 and 0.3785 μg g^–1^ at 9 days after treatment, respectively, which was only 13.31 and 3.63% of the control concentration.

**TABLE 3:** Phenolic acids degradation of strain XNRB-3 in soil.

Treatment	Time	CA	Phlorizin	BA	FA	PHBA
CK	0 Day	10.4321 ± 0.1703^a^	30.0569 ± 0.0228^a^	90.4921 ± 0.0536^a^	100.4403 ± 0.1695^a^	20.6373 ± 0.1954^a^
	3 Days	6.6133 ± 0.0349^b^	23.8619 ± 0.5910^c^	83.8100 ± 0.2810^b^	83.8655 ± 0.7399^b^	18.3768 ± 0.2350^b^
	6 Days	5.7945 ± 0.0349^c^	24.9330 ± 0.0137^b^	77.0167 ± 0.4360^c^	71.1995 ± 0.3209^c^	12.2645 ± 0.1751^c^
	9 Days	5.6424 ± 0.3036^cd^	24.1495 ± 0.5873^bc^	67.5674 ± 0.6518^d^	62.6178 ± 0.2685^d^	10.3960 ± 0.2109^d^
Strain XNRB-3	0 Day	10.4321 ± 0.1703^a^	30.0569 ± 0.0228^a^	90.4921 ± 0.0536^a^	100.4403 ± 0.1695^a^	20.6373 ± 0.1954^a^
	3 Days	5.2219 ± 0.1101^e^	14.4323 ± 0.2139^d^	44.6154 ± 0.0853^e^	48.1822 ± 0.6503_e_	12.5532 ± 0.0382^c^
	6 Days	1.6232 ± 0.0377^f^	9.4797 ± 0.1108^e^	35.6996 ± 0.1247^f^	31.8586 ± 0.0451^f^	8.9623 ± 0.0035^e^
	9 Days	0.3785 ± 0.0045^g^	2.9292 ± 0.0120^f^	18.4953 ± 0.0370^g^	13.3669 ± 0.0380^g^	5.2882 ± 0.0404^f^

*The soil samples were added with phlorizin, cinnamic acid (CA), ferulic acid (FA), benzoic acid (BA), and P-hydroxybenzoic acid (PHBA) (μg g^–1^). Values in columns followed by the same letter are not significantly different according to Duncan test at p < 0.05. Values are mean ± SD (n = 3).*

### The Protective Effect of Strain XNRB-3 on Plant Roots

Strain XNRB-3 can form a thick biofilm in a static medium. After 14 days of planting, the population of strain XNRB-3 colonized on root tissue was approximately 7.30 × 10^6^ CFU g^–1^. The population sharply decreased to 6.47 × 10^4^ CFU g^–1^ after 35 days (Supplementary Figure 9b). The conidia and hyphae of *Fusarium* treated with strain XNRB-3 only attached to root epidermal cells (Supplementary Figures 10A–D). *Fusarium* hyphae invaded the cortical cells through the intercellular layer and then entered the vascular column, and a large amount of cell content (sticky substances and starch granules) was also observed in the cortical cells and vascular column (Supplementary Figures 9a, 10).

### Disease Severity Assessment

In the greenhouse test, the symptoms of Fusarium wilt appeared 7 days after the plant seedlings were transplanted into the infected soil. The incidence of *Fusarium* wilt increased rapidly over the next 28 days, and the disease severity index increased progressively over the experimental period and ultimately reached 4.00 ± 0.00; a reduction in disease progress was noted in plants treated with strain XNRB-3 (Table 4). In the fifth week, the disease index and relative control effect of plant seedlings inoculated with strain XNRB-3 were both stable at 33 and 50%, respectively. During the test period, plants inoculated with sterile distilled water remained healthy. The disease symptoms were the result of artificial infection with *Fusarium*, which was successfully re-isolated from inoculated plants at the end of the experiment. According to RT-PCR, the size of the *Fusarium* populations of the two treatments significantly increased after 21 days of planting in the infected soil. Compared with the CK treatment (5.50 × 10^5^ copies g^–1^ soil), the size of the *Fusarium* population treated with strain XNRB-3 (2.37 × 10^5^ copies g^–1^ soil) was significantly reduced (Supplementary Figure 9c). These results were consistent with the incidence of the aforementioned diseases.

The same results were obtained in the outdoor pot experiment. The biomass of apple plants treated with strain XNRB-3 (T2) in September was significantly higher than that of CK1. The plant height, ground diameter, fresh weight, and dry weight were increased by 88.07, 61.28, 181.35, and 140.44%, respectively, which were second only to the methyl bromide fumigation treatment (Supplementary Figure 11). Treatment with strain XNRB-3 also significantly enhanced the root growth of apple plants (Figures 5e,i,j,l,m). In September, the plants had grown considerably, and the root length, surface area, number of tips, and number of forks were significantly lower in CK1 than in CK2 and T2. The length, surface area, number of tips, and number of forks were 1.73, 2.62, 5.67, and 2.65 times higher in T2 than in CK1. The root respiration rate and the SOD, POD, and CAT activity increased from July to September in T2 and CK2 (Figures 5e–h,k,n). In September, the root respiration rate in T2 was 1037.98 μmol O_2_⋅g^–1^ FW⋅min^–1^, which was 1.49 and 1.32 times higher compared with that observed in CK1 and T1. The activity of SOD, POD, and CAT was 1.50, 2.93, and 1.72 times higher in T2 than in CK1. The MDA content showed the opposite trend: the MDA content was 29.96, 60.35, and 78.13% lower in July, August, and September, respectively, in T2 compared with CK1.

**TABLE 4:** Percentage of dead plants, FMS, and AUDPC of *Malus hupehensis* Rehd. seedlings after inoculation strain XNRB-3.

Treatment		Investigation time	DI (%)	Relative control effect (%)	Incidence (%)	PDP (%)	FMS	AUDPC
Negative control		Weeks 1–5	0.00 ± 0.00	–	0.00 ± 0.00	0.00 ± 0.00	0.00 ± 0.00	0.00 ± 0.00
Positive control	*Fusarium proliferatum*	Week 1	34.45 ± 3.64^a^	–	64.44 ± 8.01^a^	0.00 ± 0.00^c^	2.15 ± 0.05^a^	30.14 ± 3.19^a^
		Week 2	67.22 ± 0.55b	–	100 ± 0.00^a^	55.55 ± 2.22^a^	2.69 ± 0.02^a^	88.96 ± 3.67^a^
		Week 3	87.78 ± 1.47a	–	100 ± 0.00^a^	68.89 ± 2.22^b^	3.51 ± 0.06^a^	135.62 ± 1.46^a^
		Week 4	100 ± 0.00a	–	100 ± 0.00^a^	100 ± 0.00^a^	4.00 ± 0.00^a^	149.97 ± 1.35^a^
		Week 5	100 ± 0.00a	–	100 ± 0.00^a^	100 ± 0.00^a^	4.00 ± 0.00^a^	284.96 ± 1.62^a^
	*Fusarium verticillioides*	Week 1	33.89 ± 0.65a	–	68.89 ± 2.22^a^	0.00 ± 0.00	1.97 ± 0.03^a^	29.65 ± 0.49^a^
		Week 2	70.00 ± 1.67^ab^	–	100 ± 0.00^a^	48.89 ± 2.22^ab^	2.80 ± 0.07^a^	90.90 ± 1.94^a^
		Week 3	87.78 ± 0.55^a^	–	100 ± 0.00^a^	68.89 ± 2.22^b^	3.51 ± 0.02^a^	138.06 ± 1.75^a^
		Week 4	100 ± 0.00^a^	–	100 ± 0.00^a^	100 ± 0.00^a^	4.00 ± 0.00^a^	151.18 ± 1.06^a^
		Week 5	100 ± 0.00^a^	–	100 ± 0.00^a^	100 ± 0.00^a^	4.00 ± 0.00^a^	286.42 ± 1.27^a^
	*Fusarium oxysporum*	Week 1	30.56 ± 1.11^a^	–	64.44 ± 5.88^a^	0.00 ± 0.00	1.92 ± 0.12^a^	26.74 ± 0.97^a^
		Week 2	70.00 ± 1.92^ab^	–	100 ± 0.00^a^	48.89 ± 2.22^ab^	2.80 ± 0.08^a^	87.99 ± 2.57^a^
		Week 3	91.67 ± 0.96^a^	–	100 ± 0.00^a^	80.00 ± 0.00^a^	3.67 ± 0.04^a^	141.46 ± 1.46^a^
		Week 4	100 ± 0.00^a^	–	100 ± 0.00^a^	100 ± 0.00^a^	4.00 ± 0.00^a^	154.59 ± 0.84^a^
		Week 5	100 ± 0.00^a^	–	100 ± 0.00^a^	100 ± 0.00^a^	4.00 ± 0.00^a^	290.50 ± 1.01^a^
	*Fusarium solani*	Week 1	35.00 ± 0.00^a^	–	64.44 ± 4.44^a^	0.00 ± 0.00	2.19 ± 0.14^a^	30.63 ± 0.00^a^
		Week 2	72.22 ± 2.00^a^	–	100 ± 0.00^a^	42.22 ± 5.88^b^	2.89 ± 0.08^a^	93.82 ± 1.75^a^
		Week 3	88.33 ± 1.67^a^	–	100 ± 0.00^a^	71.11 ± 2.22^b^	3.53 ± 0.07^a^	140.49 ± 3.19^a^
		Week 4	100 ± 0.00^a^	–	100 ± 0.00^a^	100 ± 0.00^a^	4.00 ± 0.00^a^	152.64 ± 2.32^a^
		Week 5	100 ± 0.00^a^	–	100 ± 0.00^a^	100 ± 0.00^a^	4.00 ± 0.00^a^	288.17 ± 2.78^a^
Strain XNRB-3	*Fusarium proliferatum*	Week 1	1.11 ± 0.56^b^	96.39 ± 1.81^ab^	4.44 ± 2.22^c^	0.00 ± 0.00	0.67 ± 0.33^b^	0.97 ± 0.49^b^
		Week 2	8.33 ± 0.00^cd^	87.60 ± 0.10^b^	26.67 ± 3.85^c^	0.00 ± 0.00^c^	1.31 ± 0.19^b^	8.26 ± 0.49^b^
		Week 3	12.22 ± 1.11^b^	86.11 ± 1.07^a^	33.33 ± 3.85^b^	0.00 ± 0.00^c^	1.48 ± 0.08^bc^	17.99 ± 0.97^b^
		Week 4	23.89 ± 0.56^d^	76.11 ± 0.56^a^	42.22 ± 8.01^b^	6.67 ± 0.00^b^	2.46 ± 0.52^b^	24.79 ± 0.73^b^
		Week 5	35.56 ± 0.56^d^	64.44 ± 0.56^a^	68.89 ± 2.22^b^	6.67 ± 0.00^c^	2.07 ± 0.03^d^	60.96 ± 0.77^d^
	*Fusarium verticillioides*	Week 1	4.44 ± 0.56^b^	86.83 ± 1.83^c^	17.78 ± 2.22^b^	0.00 ± 0.00	0.67 ± 0.33^b^	3.89 ± 0.49^b^
		Week 2	7.22 ± 0.55^d^	89.71 ± 0.53^a^	24.45 ± 2.22^c^	0.00 ± 0.00^c^	1.19 ± 0.10^b^	10.21 ± 0.00^b^
		Week 3	14.44 ± 3.09^b^	83.59 ± 3.44^a^	40.00 ± 6.67^b^	0.00 ± 0.00^c^	1.42 ± 0.09^c^	18.96 ± 3.03^b^
		Week 4	30.56 ± 0.56^b^	69.44 ± 0.56^b^	53.33 ± 3.85^b^	0.00 ± 0.00^c^	2.31 ± 0.14^b^	29.17 ± 2.95^b^
		Week 5	48.33 ± 0.96^b^	51.67 ± 0.96^c^	66.67 ± 3.85^b^	20.00 ± 0.00^b^	2.91 ± 0.11^b^	76.42 ± 3.66^b^
	*Fusarium oxysporum*	Week 1	2.78 ± 0.55^b^	90.82 ± 1.99^bc^	11.11 ± 2.22^bc^	0.00 ± 0.00	0.67 ± 0.33^b^	2.43 ± 0.49^b^
		Week 2	6.67 ± 0.00^d^	90.46 ± 0.26^a^	22.22 ± 2.22^c^	0.00 ± 0.00^c^	1.22 ± 0.11^b^	8.26 ± 0.49^b^
		Week 3	12.78 ± 0.55^b^	86.07 ± 0.49^a^	31.11 ± 2.22^b^	0.00 ± 0.00^c^	1.65 ± 0.05^b^	17.01 ± 0.49^b^
		Week 4	26.11 ± 0.56^c^	73.89 ± 0.56^a^	44.44 ± 5.88^b^	6.67 ± 0.00^b^	2.45 ± 0.38^b^	25.52 ± 0.42^b^
		Week 5	33.89 ± 1.11^d^	66.11 ± 1.11^a^	62.22 ± 4.44^b^	6.67 ± 0.00^c^	2.19 ± 0.09^d^	62.13 ± 0.00^d^
	*Fusarium solani*	Week 1	0.56 ± 0.56^b^	98.41 ± 1.59^a^	2.22 ± 2.22^c^	0.00 ± 0.00	0.33 ± 0.33^b^	0.94 ± 1.94^b^
		Week 2	11.11 ± 0.56^c^	84.56 ± 1.11^c^	37.78 ± 2.22^b^	0.00 ± 0.00^c^	1.18 ± 0.01^b^	10.21 ± 0.84^b^
		Week 3	13.89 ± 1.11^b^	84.24 ± 1.43^a^	40.00 ± 0.00^b^	0.00 ± 0.00^c^	1.39 ± 0.11^c^	21.88 ± 0.84^b^
		Week 4	25.00 ± 0.96^cd^	75.00 ± 0.96^a^	46.67 ± 3.85^b^	0.00 ± 0.00^c^	2.18 ± 0.25^b^	27.95 ± 1.06^b^
		Week 5	41.11 ± 1.47^c^	58.89 ± 1.47^b^	64.44 ± 5.88^b^	6.67 ± 0.00^c^	2.58 ± 0.15^c^	68.25 ± 1.82^c^

*Values in columns followed by the same letter are not significantly different according to Duncan test at p < 0.05. Values are mean ± SD (n = 3).*

*DI, disease intensity; Incidence, percentage of diseased plants; PDP, percentage of dead plants; FMS, final mean severity of symptoms; AUDPC, area under the disease progress curve.*

### Effect of Strain XNRB-3 on the Biomass of Young Apple Trees

**FIGURE 6:**
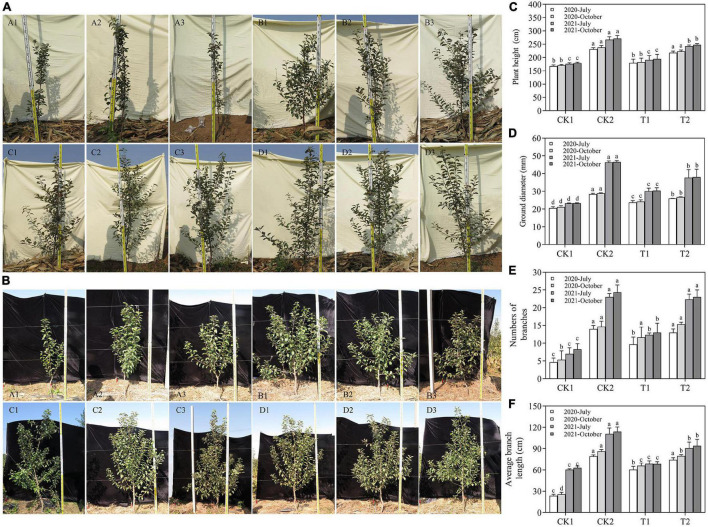
The biomass of young apple trees in different treatments, including plant height **(C)**, ground diameter **(D)**, numbers of branches **(E)**, and average branch length **(F)**. **(A)** The growth of young apple trees in different treatments in October 2020. **(B)** The growth of young apple trees in different treatments in October 2021. (A1–A3) CK1 28-year-old orchard soil, (C1–C3) CK2 Methyl bromide fumigation, (B1–B3) T1 Fertilizer carrier, (D1–D3) T2 XNRB-3 bacterial fertilizer. Values in columns followed by the same letter are not significantly different according to Duncan test at *p* < 0.05. Values are mean ± SD (*n* = 3).

The XNRB-3 bacterial fertilizer treatment (T2) significantly promoted the growth of young apple trees, and this difference became significant in October 2021 (Figures 6A,B). In July and October in 2020 and 2021, the biomass indicators were significantly lower in CK1 and T1 than in CK2 and T2. In October 2021, the plant height, ground diameter, number of branches, and average branch length increased by 38.10, 62.79, 49.17, and 176.00% in T2 relative to CK1, respectively, and the plant height, ground diameter, number of branches, and average branch length were 1.27, 1.25, 1.37, 1.77, 2.45, and 2.21-fold higher in T2 than in T1, respectively (Figures 6C–F).

### Effect of Strain XNRB-3 on the Soil Environment

**FIGURE 7:**
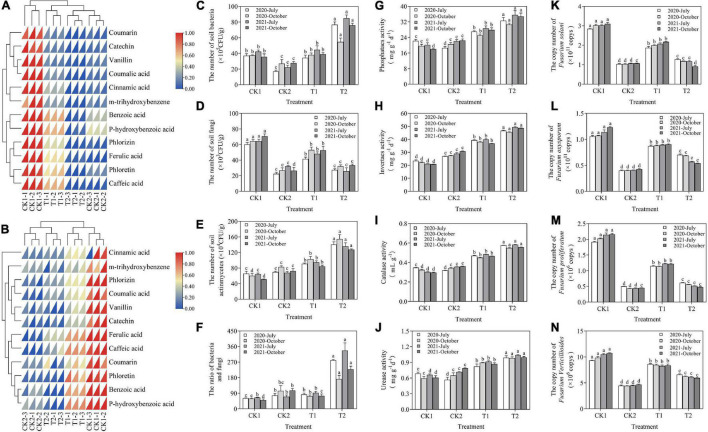
**(A)** Effects of different treatments on soil phenolic acid in October 2020. **(B)** Effects of different treatments on soil phenolic acid in October 2021. **(C–F)** The effect of strain XNRB-3 on the density of microorganisms in the rhizosphere of young apple trees. **(G–J)** Effect of different treatments on soil enzyme activities. **(K–N)** Effect of different treatments on the gene copy number of four fungal pathogens by using real-time qPCR. CK1: 28-year-old orchard soil, CK2: Methyl bromide fumigation, T1: Fertilizer carrier, T2: XNRB-3 bacterial fertilizer. Values in columns followed by the same letter are not significantly different according to Duncan test at *p* < 0.05. Values are mean ± SD (*n* = 3).

In October of 2020 and 2021, the soil phenolic acid content was highest in CK1 and T1 and lowest in CK2 and T2 (Figures 7A,B). The total soil phenolic acid content was significantly lower in T2 than in CK1. In October 2020, the soil content of cinnamic acid, phlorizin, benzoic acid, ferulic acid, and *p*-hydroxybenzoic acid was 54.38, 74.02, 77.29, 54.70, and 71.83% lower, respectively, in T2 than in CK1. In October 2021, the soil content of cinnamic acid, phlorizin, benzoic acid, ferulic acid, and *p*-hydroxybenzoic acid was 87.23, 91.95, 89.80, 84.11, and 91.98% lower, respectively, in T2 than in CK1.

The activity of urease, phosphatase, sucrase, and catalase increased steadily in T1 and T2 in July and October in 2020 and 2021 relative to CK1. The soil enzyme activity decreased in the first year of the fumigation treatment; it then continued to increase, increasing most significantly in T2 (Figures 7G–J). In October 2020 and 2021, the urease activity was 1.65-fold and 1.64-fold higher, the phosphatase activity was 1.56-fold and 1.93-fold higher, the sucrase activity was 2.03-fold and 2.34-fold higher, and the catalase activity was 1.69-fold and 1.87-fold higher in T2 than in CK1, respectively. After 2 years of applying strain XNRB-3, the physical and chemical properties of plant rhizosphere soil were significantly improved. Compared with CK1, organic matter, total nitrogen, total phosphorus, total potassium, available potassium, available phosphorus, NH_4_^+^-N, nitrate nitrogen, and soil pH increased by 228.54, 245.25, 242.29, 30.53, 327.89, 128.64, 56.05, 239.61, and 7.43%, respectively, in T1. Various nutrient indexes of the soil were also increased in T1, indicating that the addition of organic amendments could enhance the nutrient conditions of the soil (Supplementary Figure 12).

In July and October of 2020 and 2021, the number of soil bacteria in the XNRB-3 bacterial fertilizer treatment (T2) increased significantly, and compared with CK1, the number of soil bacteria was increased by 107.21, 45.13, 100.79, and 113.08% in T2, respectively (Figure 7C). There were significant differences in the number of soil fungi between different treatments. Compared with CK1, the number of soil fungi in CK2, T1, and T2 was significantly reduced, the number of soil fungi in T1 was significantly higher than that in T2, and the effect of T2 was similar to the fumigation treatment. In October 2020, 2021, the number of soil fungi in CK2 and T2 was reduced by 58.55, 50.26%, and 62.74, 52.83% compared with CK1, respectively (Figure 7D). The number of actinomycetes and the ratio of bacteria/fungi in the soil were significantly higher in T2 than in CK1 (Figures 7E,F).

The qPCR results showed that the abundance of *Fusarium* was significantly reduced in July and October of 2020 and 2021 in CK2 and T2 compared with CK1 and T1 (Figures 7K–N). The abundance of *F. proliferatum*, *F. solani, F. verticillioides*, and *F. oxysporum* was 50.43, 40.74, 25.96, and 22.20% lower in T2 relative to T1 in October 2020, and 60.43, 57.97, 28.69, and 40.31% lower in T2 relative to T1 in October 2021, respectively.

### Effect of Strain XNRB-3 on the Soil Microbial Community

**FIGURE 8:**
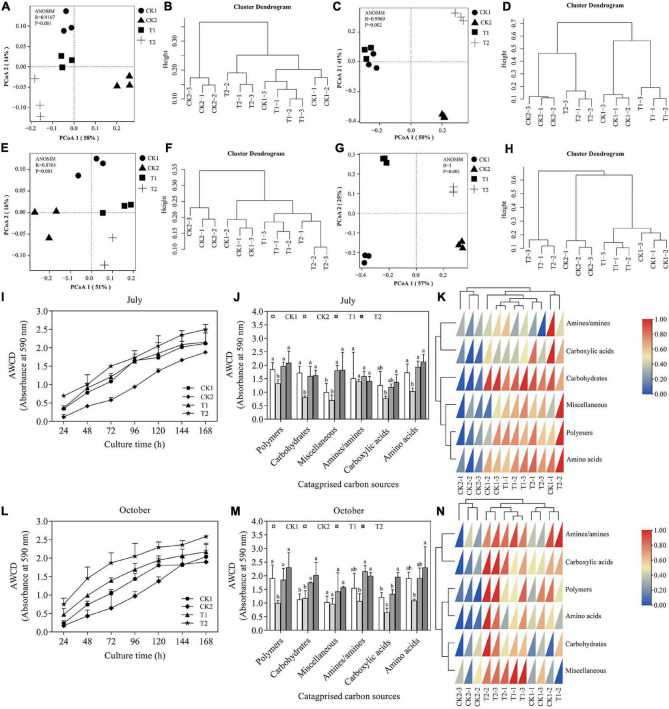
**(A,B,I–K)** (October, 2020), **(E,F,L–N)** (October, 2021): Utilization of different carbon substrates by rhizosphere soil samples under different treatments using Biolog Eco plates. **(A,B,E,F)** The PCA of potential community functional diversity of soil microbes. **(I,L)** Variation in average well color development (AWCD) after 168 h of incubation. **(J,K,M,N)** Categorized substrates utilization pattern by microbial communities from rhizosphere soils after 96 h of incubation. **(G,H,** bacteria) and **(C,D,** fungi): Microbial community structures in the different treatments based on the T-RFLP data in October 2021. Principal coordinate analysis (PCoA) **(C,G)** and cluster analysis **(D,H)** plot based on the OTUs of Bray_Curtis distance. *R* = 1 > 0, *p* < 0.05 means that the difference between groups is greater than the difference within groups, and there are significant differences between different treatments. CK1: 28-year-old orchard soil, CK2: Methyl bromide fumigation, T1: Fertilizer carrier, T2: XNRB-3 bacterial fertilizer. Values in columns followed by the same letter are not significantly different according to Duncan test at *p* < 0.05. Values are mean ± SD (*n* = 3).

In the present study, AWCD was used as an indicator of soil microbial activity. The variation in AWCD with incubation time is shown in Figures 8I,L. Here, AWCD increased as the incubation time extended for all treatments in October 2020 and 2021. The soil with strain XNRB-3 (T2) had a higher AWCD value than soil in other treatments, signifying that the addition of strain XNRB-3 increased the activity of microorganisms. After 120 h, the AWCD values of T1 and CK1 were similar, and the AWCD value was lower in CK2, indicating that fumigation can inhibit the activity of microorganisms for a longer period.

Biolog-ECO plates have six categories of carbon sources: carbohydrates, carboxylic acids, amino acids, polymers, phenolic compounds, and amines (Guo et al., 2015). In October 2020 (Figures 8I–K), the OD value of three types of carbon sources (polymers, miscellaneous, carboxylic acids, and amino acids) increased in T2 relative to CK1, but did not reach significant levels except for miscellaneous. In contrast, amines/amines and carbohydrates were reduced in T2, but did not reach significant levels. The effect of strain XNRB-3 treatment (T2) on the utilization rate of the four substrate groups (polymers, miscellaneous, carboxylic acids, and amino acids) was stronger compared with the other treatments. The utilization rate of these four substrate groups was 6.11, 0.97, 15.41, and 10.24% higher in T2 than in T1, respectively. In October 2021 (Figures 8M,N), the changes in the utilization rate of microbial substrates under different soil treatments varied starting in 2020. Compared with CK1, the OD values of the two types of carbon sources (carbohydrates and carboxylic acids) increased significantly in T2, and the other four carbon sources (polymers, miscellaneous, amines/amines, and amino acids) also increased but did not reach significance levels. With the exception of miscellaneous, the OD values of amines/amines, carboxylic acids, carbohydrates, polymers, and amino acids increased by 40.79, 42.17, 24.45, 10.56, and 7.91% in 2021, respectively, compared with 2020. These findings indicate that the soil microorganisms treated by strain XNRB-3 mainly use polymers, carboxylic acids, and amino acids. The utilization rate of the six types of carbon sources in CK2 was significantly lower compared with the other treatments, denoting that fumigation greatly affected the microbial community in the soil.

A principal component analysis and cluster analysis demonstrated that the soil microbial community structure in T2 and CK2 significantly differed from that in CK1 (Figures 8A–H). The soil bacterial community structure in T2 differed significantly from that in other treatments (Figures 8G,H), and the soil fungal and bacterial communities of T1 and CK1 were similar (Figures 8C,D,G,H). The Margalef, McIntosh, Brillouin, Simpson, and Shannon indexes reflect the richness and diversity of soil microbial communities (Supplementary Tables 28, 29). The abundance of soil fungi and bacterial communities was significantly increased after adding strain XNRB-3. The diversity of soil fungi communities was significantly increased, the dominance index and carbon source utilization were significantly reduced, and the bacterial community showed the opposite pattern. The functional diversity of the microbial community was analyzed based on the data of 96-h Biolog Eco plates in 2020 and 2021, the functional diversity of the microbial community in T1 and T2 showed similar changes. The diversity of soil microbial communities was significantly increased in T1 and T2 than in CK1, which was consistent with the results of the T-RFLP analysis.

These results indicated that XNRB-3 bacterial fertilizer can also improve the soil microbial environment along with its biocontrol activity.

## Discussion

In recent years, the introduction of beneficial microorganisms into soil has been shown to be an attractive alternative for controlling plant diseases, especially endophytic strains with antibacterial effects or strains that promote plant growth; however, these microorganisms have been rarely used to control ARD (Nan et al., 2011; Deketelaere et al., 2017). Cao et al. (2011) found that *B. subtilis* SQR 9 isolated from a healthy cucumber root in a field with a high incidence of *Fusarium* wilt disease can control cucumber wilt by colonizing plant roots. In the present study, an endogenous *B. licheniformis* XNRB-3 was isolated from the root tissues of healthy fruit trees in orchards where the incidence of ARD was high. Furthermore, this strain could stably colonize the roots of apple seedlings, showing high phlorizin-degrading activity and multiple PGP properties. It could also significantly inhibit the mycelial growth and spore germination of *Fusarium* by producing antifungal compounds. The inhibition of spore germination is essential for the development of fungal disease during the early stage (Miyara et al., 2010). This revealed that the strain XNRB-3 has the potential to be used as a biological control agent to control ARD.

Previous studies indicate that many beneficial endophytes *Bacillus* (*B. subtilis*, *B. amyloliquefaciens*, and *B. velezensis*), exhibit several disease suppression mechanisms and promote plant growth, including preventing vascular pathogens from colonizing ecological niches, biocontrol attributes [the production of siderophores, hydrolytic enzymes, hydrogen cyanide (HCN), and antibiotics], broad-spectrum antibiotics, PGP traits (IAA production, phosphate solubilization, potassium solubilization, nitrogen fixation, and ACC deaminase), and promoting induced systemic resistance (Penrose and Glick, 2003; Ahmad et al., 2008; Senthilkumar et al., 2009; Kumar et al., 2012; Vacheron et al., 2013; Eljounaidi et al., 2016; Dowarah et al., 2021). The endogenous *B. licheniformis* XNRB-3 isolated in this study also has several of the aforementioned PGP properties and antagonistic traits. For example, IAA, CTK, and GA can significantly increase the root length, root tip, and branch number of apple seedlings, the production of IAA can also promote the establishment of a symbiotic relationship between plants and arbuscular mycorrhizal (AM) fungi to improve their adaptability to the external environment (Wahyudi et al., 2011; Liao et al., 2015); nitrogen fixation, ACC deaminase, and ammonia production can induce plant resistance and promote plant growth (Penrose and Glick, 2003; Senthilkumar et al., 2009); and phosphate and potassium solubilization can effectively increase the absorption of phosphorus and potassium by plants and promote root development (Richardson, 2001). Strain XNRB-3 can also produce enzymes that dissolve fungal cell walls (cellulose, pectinase, β1,3-glucanase, chitosanase, and protease), siderophores, antifungal compounds (2,4-di-tert-butylphenol and alpha-bisabolol), and low molecular weight metabolites (HCN) restrict the growth of pathogens. Among them, the production of siderophores can also induce the activity of plant root protection enzymes to defend against pathogenic fungi. The production of cellulase and pectinase can also help strain XNRB-3 better colonize the root system (Verma et al., 2001; Wahyudi et al., 2011; Yu et al., 2011). Therefore, adding the strain XNRB-3 under potted and field conditions can promote the growth of apple plants.

The production and transportation of BCAs are essential for successful biological control under field conditions (Thangavelu et al., 2004). An appropriate carrier can support the survival of BCAs while inhibiting the growth of target pathogens, thereby improving the performance of BCAs for plant disease control (Ling et al., 2010; Malusá et al., 2012; Wei et al., 2015). Smith (1992) found that dry inoculants can be produced using different types of soil materials (peat, coal, clays, and inorganic soil), organic materials (composts, soybean meal, wheat bran, and sawdust), or inert materials (e.g., vermiculite, perlite, kaolin, bentonite, and silicates). In this experiment, dry inoculants, as listed in Supplementary Table 10, were used to optimize the fermentation conditions of strain XNRB-3 using RSM, which significantly increased the survival rate and shelf life of strain XNRB-3 and complies with the Chinese bio-organic fertilizer production standard stipulating that the functional microorganism content should be greater than 2.0 × 10^7^ CFU g^–1^ dry formulation after storage for 6 months at room temperature (Emmert and Handelsman, 1999). The raw materials (CDC and wheat straw) in the formula are cheap and easy to obtain, and the fermentation level is high, providing a good foundation for its large-scale industrial production.

The optimized strain XNRB-3 fermentation broth can significantly inhibit the mycelial growth and spore germination of *Fusarium*, and an abnormal structure of the mycelia (mycelia and conidia breakage, deformity, and dissolution) from the edge of the inhibition zone was observed using scanning electron microscopy (SEM) *in vitro* assays. This antagonism may be caused by the secreted antifungal compounds (Yndj protein, subtilisin, bacillomycin, iturin A, fengycin, and surfactin) (Cawoy et al., 2015; Harwood et al., 2018). Among these compounds, lipopeptides (surfactin, iturin, and fengycin families) and bacillomycin show potent antimicrobial activity against a wide variety of microorganisms *in vitro*, especially filamentous fungi (*F. oxysporum*, *Verticillium dahliae*, *P. capsici*, and *P. nicotianae*) (Cao et al., 2012; Frikha-Gargouri et al., 2017). The production of lipopeptide substances might also be one of the important reasons why strain XNRB-3 can form a biofilm on the surface of the roots (Hofemeister et al., 2004; Chen et al., 2013). Strain XNRB-3 can also produce free amino acids (aspartic acid, glutamic acid, proline, and tyrosine) during the fermentation process. The production of amino acids is closely related to the biosynthesis of peptide antibiotics (Besson et al., 1990; Ren et al., 2012).

The formation of bacterial biofilms and their ability to colonize the rhizosphere and/or roots are closely related to successful field applications (Compant et al., 2010; Zhang et al., 2011; Mendis et al., 2018). This study found that strain XNRB-3 could colonize plant roots, and its fresh weight ranged from 10^5^ to 10^7^ CFU g^–1^ within 21 days, and it can also significantly reduce the abundance of *Fusarium* in the rhizosphere soil. These findings were consistent with the results of Cao et al. (2011), the pathogen density in the rhizosphere of cucumber seedlings inoculated with *B. subtilis* SQR9 was significantly reduced. Similar results were obtained in the PAS staining test. The roots treated with the fermentation broth of the strain XNRB-3 prevents the entry of the *Fusarium* into the vascular stele, and the mycelial growth was restricted to the epidermis and outer root cortex, indicating that strain XNRB-3 can colonize the roots of the plant and grow root epidermis, forming a biofilm that prevents *Fusarium* infection and improves the resistance of plants to infection (Benhamou et al., 1998; Duan et al., 2021). Infected roots can also produce a large amount of a sticky substance, which results in the deposition of formed callose and starch granules to form a mechanical barrier that inhibits the invasion of pathogens (Lagopodi et al., 2002; Grunewaldt-Stöcker et al., 2020).

Soil enzyme activity and soil attributes (e.g., SOC, TN, TP, AN, and AP concentrations) are often used to monitor changes in soil microbial activity and soil fertility (Kandeler et al., 2006; Liu et al., 2011; Song et al., 2012; Pan et al., 2013). This study found that the application of strain XNRB-3 significantly increased the activity of soil-related enzymes, and it increased after the second year of applying strain XNRB-3. This was consistent with the results of several previous studies. For example, the addition of PGPR can significantly improve the physical and chemical properties of soil and soil enzyme activity (Ren et al., 2019). PGPR strains can induce the production of lytic enzymes by utilizing carbon from the cell wall of microorganisms, thereby increasing soil urease activity (Karthik et al., 2017). The increase in urease activity indicates that the application of XNRB-3 may increase the gross N mineralization rate because urease can catalyze the hydrolysis of urea into CO_2_ and NH_4_^+^, as well as promote the soil nitrogen cycle (Xu et al., 2015). The increase in invertase activity can promote the conversion of carbohydrates and increase the concentration of soil nutrients (e.g., N, P, and K) under the action of microorganisms and improve soil fertility (Han et al., 2017). Strain XNRB-3 can increase AP activity, which increases the availability of soluble P and promotes plant growth (Krämer, 2000). After adding the strain XNRB-3, the contents of N, P, K, and SOC in the soil and the activity of soil-related enzymes were consistent. The change in soil pH value was opposite, which was consistent with the results of previous studies (Xie et al., 2017; Fu et al., 2018; Ren et al., 2019). The increase in the nutrient concentration in the soil might also be related to the characteristics of strain XNRB-3, such as nitrogen fixation, phosphorus dissolution, and potassium dissolution.

The functional diversity and overall activity of microbial communities in soil can reflect soil quality (Islam et al., 2011). Biological methods are some of the main methods used to measure the functional diversity and overall activity of microbial communities for their simple operation, high sensitivity and resolution, and rich data (Dang et al., 2015; Guo et al., 2015). Therefore, biological methods were used in this study to investigate the functional diversity and carbon source utilization of the rhizosphere soil microbial community after treatment with strain XNRB-3, as well as evaluate the safety of its use in the soil environment. The AWCD value of rhizosphere soil was significantly higher after the addition of strain XNRB-3 compared with other treatments, and also significantly enhanced the use of carbon sources such as polymers, carboxylic acids, and amino acids, which might be related to the increase in the number of soil bacteria (e.g., *Pseudomonas* spp., *Enterobacter* spp.) and actinomycetes after the addition of strain XNRB-3 (Heuer et al., 1995; Grayston et al., 1998; Larkin, 2003). Biolog GEN III microplate identification revealed that strain XNRB-3 can use a wide range of carbon sources, which permits this strain to grow and reproduce in environments with different nutrient levels (Schutter and Dick, 2001).

Previous studies have shown that the occurrence of ARD is closely related to the structure and diversity of soil microbial communities. Increases in the number of rhizosphere soil pathogens and decreases in the number of beneficial microorganisms are also important factors leading to disease outbreaks (Kelderer et al., 2012; Mazzola and Manici, 2012). The T-RFLP data from this study showed that strain XNRB-3 can significantly alter the structure of the rhizosphere soil fungal and bacterial communities after treatment, which was similar to the community structure of fumigation treatment. Application of this strain also significantly increased the abundance and diversity of rhizosphere soil bacteria and fungi, reduced the relative abundance of *Fusarium*, provided a stable and beneficial rhizosphere ecosystem for plants, and promoted plant growth (Liu L. et al., 2018). The addition of strain XNRB-3 may promote the aggregation of some beneficial microorganisms or secrete some VOCs to inhibit the growth of soil fungi and increases soil bacterial biomass, thereby improving the soil microbial environment, promoting the replanted young apple trees growth, and reducing the damage of ARD (Ryu et al., 2003; Fernando et al., 2005; Yuan et al., 2017; Liu et al., 2021).

Imbalances in the physical and chemical properties of soil and the allelopathy of root exudates and residues are considered to be the main causes of soil sickness (Zhang Y. et al., 2010; Pant et al., 2013). Yin et al. (2017) found that the roots of apple plants under continuous cropping can secrete the same substances (such as phenolic acid autotoxic substances) for a long time, and these substances significantly affect the composition and distribution of the rhizosphere microflora, increasing the number of pathogenic fungi and inhibiting plant growth (Liu H. et al., 2019; Xu et al., 2020). Phenolic substances related to currently known ARD mainly include 2,4-di-tert-butylphenol, vanillic acid, benzoic acid, p-hydroxybenzoic acid, ferulic acid, cinnamic acid, and phlorizin (Ye et al., 2006; Qu and Wang, 2008; Yin et al., 2013, 2017; Chen et al., 2021). Phlorizin is a unique phenolic acid substance of apples that mainly exists in the roots, stems, bark, tender leaves, and fruits of apples. Hofmann et al. (2009) found that the diseased seedlings cultivated in ARD soil exuded significantly more phlorizin compared to healthy seedlings, indicating that the phlorizin in root exudates was closely related to the occurrence of ARD. In the study by Zhang et al. (2009), values for phlorizin contents secreted by the roots of apple seedlings are in a range of 0.442–1.583 μg per plant. Wang et al. (2015) found that the content of phlorizin in the soil was 6.0 mg kg^–1^, which would destroy the antioxidant system of apple roots, thereby inhibiting growth. Ehrenkranz et al. (2005) found that the high concentration of phlorizin can reduce the rate of apple seedling photosynthesis and transpiration. It was also found that the growth and division of *F. moniliforme* were faster in the 1.0 mM phlorizin than in the 0.5 mM phlorizin (Yin et al., 2017). The above studies further illustrated that the long-term continuous cropping of apples can lead to the accumulation of excessive phlorizin in the rhizosphere, which directly damages the root system or indirectly affects the growth of plants by stimulating the growth of pathogenic fungi. Currently, the use of microbial degradation methods to degrade phenolic acids in the environment is becoming increasingly popular because of its various advantages, including low cost, high degradation efficiency, lack of secondary pollution, and environmental safety (Ma et al., 2018; Wang et al., 2021). Use of the medium containing phlorizin as the sole carbon source revealed that strain XNRB-3 can efficiently degrade phlorizin, and the phlorizin degradation rate could reach 68.83% after 60 h of culture under the condition of 2% inoculum. This was similar to the research results obtained by Liu et al. (2017), the phlorizin degradation rate of *Aspergillus terreus* can reach 88.96% when cultured for 96 h under the condition of 2% inoculum. Strain XNRB-3 can also effectively degrade phlorizin, cinnamic acid, ferulic acid, benzoic acid, and *p*-hydroxybenzoic acid in soil and culture fluid, thereby promoting the growth of apple seedlings. This finding was similar to the experimental results of Zhang Y. et al. (2010), which used a screening medium containing *p*-coumaric acid as the sole carbon source. Four microbes were isolated from plant soils, and these microbes could effectively degrade ferulic acid, *p*-hydroxybenzoic acid, and *p*-hydroxybenzaldehyde, as well as promote seedling growth. Phlorizin is degraded in the soil in two main ways. The first is through the hydrolysis of phloretin into phloroglucinol and *p*-hydroxyphenylpropionic acid by secreting a phloretin hydrolase, followed by the decomposition to phloretin and glucose by β-glucosidase, which is then used by bacteria (Chatterjee and Gibbins, 1969). Alternatively, it can be degraded to pyruvic acid by the protocatechuic acid pathway (Mohan and Phale, 2017), and pyruvic acid can be converted to acetyl CoA and enter the tricarboxylic acid cycle, which produces organic acids, such as citric acid, succinic acid, malic acid, and oxaloacetic acid (Priefert et al., 2001; Zhang Y. et al., 2010). These substances play an important role in promoting the absorption and transportation of certain nutrients and improving the photosynthetic efficiency of plants and the accumulation of nitrogen, phosphorus, and potassium (Liu et al., 2005). Therefore, the method of phlorizin degradation in the soil environment by the strain XNRB-3 is thought to be an effective approach for overcoming the obstacles of continuous apple cropping.

## Conclusion

**FIGURE 9:**
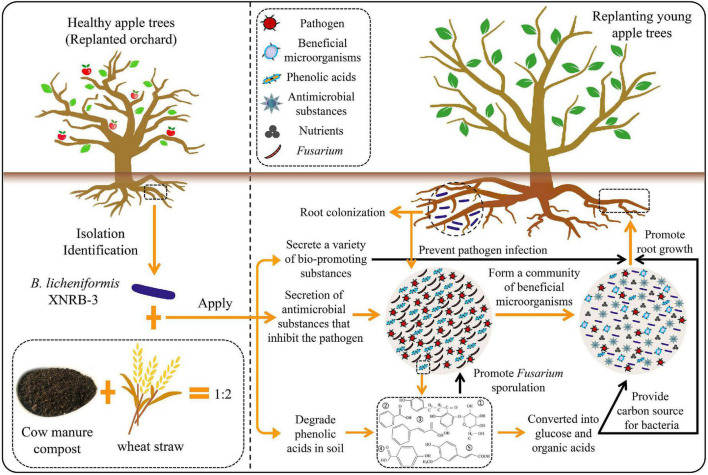
Conceptual model displaying the potential role of strain XNRB-3 influencing the pathogen density (qPCR) in the soil and the occurrence of ARD.

The phlorizin-degrading bacterium *B. licheniformis* XNRB-3 was isolated from the roots of apple plants grown in a replanted orchard. Strain XNRB-3 features a variety of PGP characteristics and antagonistic traits, which confers it with high potential for practical use, including its ability to produce some antifungal substances and significantly inhibit the spore germination of *Fusarium*. Strain XNRB-3 could effectively colonize the root surface of plant seedlings and even enter roots after it was inoculated on the roots of plant seedlings. The addition of strain XNRB-3 under potted and field conditions can significantly promote the growth of apple plants; reduce the abundance of *Fusarium* and the content of phenolic acids in the rhizosphere soil; improve the structure of the soil microbial community; increase the available nitrogen, phosphate, and potassium in the soil; and improve soil health (Figure 9). This study provides new insight and a strain resource that could be used to aid in the prevention and control of ARD.

## Data Availability Statement

The datasets presented in this study can be found in online repositories. The names of the repository/repositories and accession number(s) can be found below: https://www.ncbi.nlm.nih.gov/genbank/, MN726439.1; https://www.ncbi.nlm.nih.gov/genbank/, MT703801.1; https://www.ncbi.nlm.nih.gov/genbank/, MT713119.1; https://www.ncbi.nlm.nih.gov/genbank/, MT713122.1.

## Author Contributions

ZM and CY contributed to conception and design of the study. YD organized the database. YD, RC, RZ, and WJ performed the statistical analysis. YD wrote the first draft of the manuscript. LZ wrote sections of the manuscript. All the authors contributed to manuscript revision, read, and approved the submitted version.

## Conflict of Interest

The authors declare that the research was conducted in the absence of any commercial or financial relationships that could be construed as a potential conflict of interest.

## Publisher’s Note

All claims expressed in this article are solely those of the authors and do not necessarily represent those of their affiliated organizations, or those of the publisher, the editors and the reviewers. Any product that may be evaluated in this article, or claim that may be made by its manufacturer, is not guaranteed or endorsed by the publisher.

## Footnotes

1
http://www.phylo.org
