# Supplementary material for: The Phlorizin-Degrading Bacillus licheniformis XNRB-3 Mediates Soil Microorganisms to Alleviate Apple Replant Disease
Source: Front Microbiol. 2022 Mar 3;13:839484. doi: 10.3389/fmicb.2022.839484 (PMC8927668; doi:10.3389/fmicb.2022.839484)
Supplement: Supplementary file 1 [file Data_Sheet_1.docx]

**The Phlorizin-degrading *Bacillus licheniformis* XNRB-3 mediates soil microorganisms to alleviate apple replant disease**

Yanan Duan **^1^**, Lei Zhao**^1^**, Weitao Jiang**^1^**, Ran Chen**^1^**, Rong Zhang**^1^,** Xuesen Chen**^1^**, Chengmiao Yin**^1*^**, and Zhiquan Mao**^1*^**

^1^National Key Laboratory of Crop Biology, College of Horticulture Science and Engineering, Shandong Agricultural University, Shandong 271018, PR China

***Correspondence:**

Zhiquan Mao, Email: [mzhiquan@sdau.edu.cn](mailto:mzhiquan@sdau.edu.cn)

Chengmiao Yin, E-mail: [yinchengmiao@163.com](mailto:yinchengmiao@163.com)

**Supplementary material**

**Table S1:** Details of fungal phytopathogen culture.

| Species | Separate source | Plant disease | Deposited source | GenBank accession nr. |
| --- | --- | --- | --- | --- |
| *Fusarium proliferatum* | It was isolated from the root tissues of apple trees with Apple replant disease (ARD) symptoms in replanted orchards from Guanshui Town, Muping City, Shandong Province, China. | Apple replant disease (highly pathogenic to apple seedlings)  Diseased seedlings specifically manifested as brown from the edge of the leaf, and the spots expanded rapidly, accompanied with chlorosis. Subsequently, whole leaves turned brown to dark brown, followed by rolling and yellowing, resulting in wilting and eventually death). | China General Microbiological Culture Collection Center (CGMCC) under the accession number CGMCC No. 22426. | Accession number: ITS (MW600437.1), β-tubulin (MW670446.1), EF-1α (MW670455.1), IGS (MW670460.1), mtSSU (MW670461.1), RPB2 (MW670462.1), whole genome data ( JAFHKW000000000.1). |
| *Fusarium oxysporum* | It was isolated from the rhizosphere soil of apple trees with ARD symptoms in replanted orchards from Suizhong County, Huludao City, Liaoning Province, China. |  | They were preserved and provided by the National Key Laboratory of Crop Biology, College of Horticultural Science and Engineering, Shandong Agricultural University. | Accession number: ITS (MW600442.1), β-tubulin (MW670451.1), EF-1α (MW670459.1). |
| *Fusarium solani* | It was isolated from the rhizosphere soil of apple trees with ARD symptoms in replanted orchards from Guanli Town, Qixia City, Shandong Province, China. |  |  | Accession number: ITS (MW600441.1), β-tubulin (MW670450.1), EF-1α (MW670458.1). |
| *Alternaria alternata* | It was isolated from the root tissues of apple trees with ARD symptoms in replanted orchards from Yiyuan Town, Zibo City, Shandong Province, China. |  |  | Accession number: ITS (MW600446.1), 18S rDNA (MW600450.1). |
| *Albifimbria verrucaria* | It was isolated from tthe rhizosphere soil of apple trees with ARD symptoms in replanted orchards from Wanrong County, Yuncheng City, Shanxi Province, China. |  |  | Accession number: ITS (MW600445.1), β-tubulin (MW670454.1). |
| *Aspergillus flavus* | It was isolated from tthe rhizosphere soil of apple trees with ARD symptoms in replanted orchards from Wanrong County, Yuncheng City, Shanxi Province, China. |  |  | Accession number: ITS (MW600444.1), 18S rDNA (MW600449.1), β-tubulin (MW670453.1). |
| *Penicillium brasilianum* | It was isolated from tthe rhizosphere soil of apple trees with ARD symptoms in replanted orchards from Guanli Town, Qixia City, Shandong Province, China. |  |  | Accession number: ITS (MW600443.1), β-tubulin (MW670452.1). |
| *Fusarium verticillioides* | It was isolated from apple replant soil around the Bohai Gulf region in China. | Apple replant disease (highly pathogenic to *Malus hupehensis* seedlings). | Shandong Agricultural Microorganism Culture Collection Center, Shandong Agricultural University under the accession number AMCC300086. | / |
| *Phytophthora cactorum* | It was isolated from soil samples in Mancheng County, Baoding City, Hebei Province, China. | Strawberry blight | Agricultural Culture Collection of China under the accession number ACCC 36421. | / |
| *Rhizoctonia solani* | It was isolated from *Cucumis sativus* L. from Jinan, Shandong Province, China. | Blight of cucumber | Agricultural Culture Collection of China under the accession number ACCC 36076. | / |

**Table S2:** Basic information of sampling orchard and the number of antagonistic bacteria in different parts.

| Province | Local site | Location numbera^1^ | Latitude and longitude | Number of bacteria | | Number of antagonistic bacteria | |
| --- | --- | --- | --- | --- | --- | --- | --- |
|  |  |  |  | Root | Soil | Root | Soil |
| Shaanxi | Fu county, Yan'an city | XA | Lon:109.379711, Lat:35.98801 | 19 | 24 | 4 | 9 |
|  | Bin county, Xianyang city | XB | Lon:108.077657, Lat:35.043911 | 21 | 32 | 6 | 8 |
|  | Luochuan county, Yan'an city | XC | Lon:109.432369, Lat:35.761975 | 7 | 19 | 3 | 8 |
|  | Qian county, Xianyang city | XD | Lon:108.239472, Lat:34.527551 | 9 | 20 | 4 | 10 |
|  | Huangling county, Yan'an city | XH | Lon:109.262961, Lat:35.579428 | 9 | 11 | 3 | 9 |
|  | Liquan county, Xianyang city | XL | Lon:108.425018, Lat:34.481764 | 8 | 14 | 2 | 6 |
|  | Baishui county, Weinan city | XS | Lon:109.590670, Lat:35.177452 | 14 | 29 | 5 | 19 |
|  | Xunyi county, Xianyang city | XX | Lon:109.590670, Lat:35.177 | 12 | 21 | 4 | 11 |
| Shanxi | Fuping county, Weinan city | XF | Lon:108.333985, Lat:35.111978 | 14 | 23 | 4 | 13 |
|  | Linyi county, Yuncheng city | XE | Lon:110.774546, Lat:35.144277 | 7 | 17 | 4 | 9 |
|  | Ji county, Linfen city | XG | Lon:110.681763, Lat:36.098188 | 13 | 23 | 7 | 14 |
|  | Ruicheng county, Yuncheng city | XR | Lon:110.694369, Lat:34.69358 | 12 | 22 | 6 | 11 |
|  | Wanrong county,Yuncheng city | XW | Lon:110.838024, Lat:35.415254 | 7 | 23 | 2 | 8 |
|  | Yuncheng salt lake district | XY | Lon:110.998272, Lat:35.015101 | 15 | 19 | 8 | 14 |
|  | Jincheng city | XN | Lon:112.853555, Lat:35.501572 | 19 | 28 | 9 | 17 |
| Gansu | Jingning county, Pingliang city | XJ | Lon:105.732555, Lat:35.521977 | 19 | 24 | 4 | 11 |
|  | Kongtong district, Pingliang city | XK | Lon:106.6748350, Lat:35.542629 | 16 | 27 | 4 | 7 |
|  | Qingshui county, Tianshui city | XQ | Lon:106.137293, Lat:34.749865 | 8 | 11 | 3 | 6 |
|  | Qin'an county, Tianshui city | XT | Lon:105.674983, Lat:34.858916 | 11 | 38 | 7 | 12 |
|  | Zhuanglang county, Pingliang city | XZ | Lon:106.279101, Lat:35.267944 | 9 | 10 | 7 | 5 |

^1^Naming rules: place name abbreviation + habitat (soil, root) + microorganism type + number. For example, the first strain of bacteria screened from the root system in Zhuanglang county, Pingliang city, named XZRB-1.

| Primer name | Oligonucleotide sequence (5′–3′) | Gene | Tm °C | Reference |
| --- | --- | --- | --- | --- |
| 27F/1492R | AGAGTTTGATCCTGGCTCAG  GGTTACCTTGTTACGACTT | 16S rDNA | 55 ℃ | Somerville et al., 2020 |
| 42f/1066r | CAGTCAGGAAATGCGTACGTCCTT  CAAGGTAATGCTCCAGGCATTGCT | *gyrA* | 62 ℃ | Chun and Bae, 2000 |
| up1f/up2r | GAAGTCATCATGACCGTTCTGCAYGCNGGNGGNAARTTYGA  AGCAGGGTACGGATGTGCGAGCCRTCNACRTCNGCRTCNGTCAT | *gyrB* | 60 ℃ | Yamamoto and Harayama, 1995 |
| Prpo1/Prpo2 | A TTTCGTT AGCCGAAGAACGT  A TGTGTCCT A TTGAGACACCA | *rpoB* | 50 ℃ | Zalila-Kolsi et al., 2016 |
| JR/JF | CATACCACTTGTTGTCTCGGC  GAACGCGAATTAACGCGAGTC | *Fusarium oxysporum* | 60 ℃ | The standard curve: y= -2.291x + 36.396, R^2^=0.993 |
| CHR/CHF | GACTCGCGAGTCAAATCGCGT  GGGGTTTAACGGCGTGGCC | *Fusarium moniliforme* | 60 ℃ | The standard curve: y=-3.495x+12.421, R^2^ = 0.998 |
| CR/CF | GATCGGCGAGCCCTTGCGGCAAG  CGCCGCGTACCAGTTGCGAGGGT | *Fusarium proliferatum* | 65 ℃ | The standard curve: y= -3.675x + 9.128, R^2^=0.999 |
| FR/FF | CGAGTTATACAACTCATCAACC  GGCCTGAGGGTTGTAATG | *Fusarium solani* | 65 ℃ | The standard curve: y=-2.352x+26.941, R^2^ = 0.994 |
| 27F-FAM/1492R | AGAGTTTGATCCTGGCTCAG  GGTTACCTTGTTACGACTT | 16S rDNA | 52 ℃ | Quéric and Soltwedel, 2012 |
| ITS1F-FAM/ITS4R | CTTGGTCATTTAGAGGAAGTAA  CAGGAGACTTGTACACGGTCCAG | ITS | 50 ℃ | Gardes and Bruns, 1993 |

**Table S3:** Oligonucleotide primers and probes used used in this experiment.

**Table S4:** Primers for PCR detection of antibiotics biosynthesis genes in *Bacillus licheniformis* XNRB-3.

| Antibiotics | Target gene | Code | Sequences (5’-3’) | Amplicon size (bp) | PCR reaction system | PCR reaction program | References |
| --- | --- | --- | --- | --- | --- | --- | --- |
| Fengycin | *fen* | Af2-F/Tf1-R | GAATAYMTCGGMCGTMTKGA  GCTTTWADKGAATSBCCGCC | 452 | 25 μL reaction volume: 1 μL genomic DNA, 2.5 μL 10× PCR buffer, 20 mM MgCl_2_, 0.2 mM of each dNTP, 0.5 μM of each primer and 1.25 U Taq DNA polymerase (Takara, Dalian, China) | The initial denaturation of 94 °C for 5 min, followed by 35 cycles of 94 °C for 30 s, annealing for 30 s at 52 °C for *ituC*, *yndJ*, *bamC* and *sboA* specific primers, whereas those for *srf*, *srfAB*, *ituD*, *fen*, *fenB* and *qk* were set to 58 °C and 72 °C for 1 min, and extension at 72 °C for 7 min. | Hussein et al., 2017 |
| Surfactin | *srf* | As1-F/Ts2-R | CGCGGMTACCGVATYGAGC  ATBCCTTTBTWDGAATGTCCGCC | 419 |  |  | Hussein et al., 2017 |
| Iturin A | *ituD* | ituD-F/ituD-R | ATG AAC AAT CTT GCC TTT TTA  TTA TTT TAA AAT CCG CAA TT | 1203 |  |  | Hsieh et al., 2004 |
| Subtilosin | *sboA* | Sbo1F/Sbo1R | TCGGTTTGTAAACTTCAACTGC  GTCCACTAGACAAGCGGCTC | 334 |  |  | Cao et al., 2012 |
| Yndj | *yndJ* | 147F/147R | CAGAGCGACAGCAATCACAT  TGA ATT TCG GTC CGC TTA TC | 212 |  |  | Cao et al., 2012 |
| Subtilisin | *qk* | Qk1F/Qk1R | CTTAAACGTCAGAGGCGGAG  ATTGTGCAGCTGCTTGTACG | 704 |  |  | Cao et al., 2012 |
| Surfactin | *srfAB* | 110F/110R | GTTCTCGCAGTCCAGCAGAAG  GCCGAGCGTATCCGTACCGAG | 308 |  |  | Cao et al., 2012 |
| Bacillomycin | *bamC* | bamC2F/bamC2R | CTGGAAGAGATGCCGCTTAC  AAGAGTGCGTTTTCTTCGGA | 850 |  |  | Cao et al., 2012 |
| Iturin | *ituC* | ITUCF1/ITUCR3 | TTCACTTTTGATCTGGCGAT  CGTCCGGTACATTTTCAC | 575 |  |  | Cao et al., 2012 |
| Fengycin | *fenB* | FenB1F/FenB1R | TACCAATCGCAATGTCGTGT  CTTCGATTTCTAACAGCCGC | 767 |  |  | Cao et al., 2012 |

**Table S5:** Single factor test content.

| Culture ingredients | Single factor | Concentration |
| --- | --- | --- |
| Carbon sources | sucrose, maltose, glucose, lactose, soluble starch | 0.5 %, 1.0 %, 1.5 %, 2.0 %, 2.5 % |
| Nitrogen sources | yeast extract, peptone, (NH_4_)_2_SO_4_, beef extract, NH_4_NO_3_ , Urea, NH_4_Cl | 0.1 %, 0.5 %, 1.0 %, 1.5 %, 2.0 % |
| Inorganic salts | KH_2_PO_4_, CaCl_2_, MgSO_4_, NaCl, MnSO_4_, KCl | 0.02 %, 0.05 %, 0.1 %, 0.15 %, 0.20% |
| Culture condition | Gradient | |
| Filling volume | 25, 50, 100, 125, and 150 mL (250-mL Erlenmeyer flask) | |
| Speeds | 100, 150, 180, 200, and 250 rpm | |
| Temperatures | 25, 30, 37, 40, and 50 ℃ | |
| pH | 5.5, 6.5, 7.0, 7.5, 8.0, 8.5, and 9.5 (use HCl (0.1mol L^-1^) and NaOH (0.1 mol L^-1^) to adjust) | |

**Table S6:** Multiple plant growth promoting activities shown by strain XNRB-3.

| Function | Function name | Detection method | Positive result | References | Result |
| --- | --- | --- | --- | --- | --- |
| PGP properties | Phosphate solubilization | Bacteria was grown in Pikovskaya (PVK) medium (Glucose 10 g, Ca_3_(PO_4_)_2_ 5 g, NaCl 0.2 g, (NH_4_)_2_SO_4_ 0.5 g, MgSO_4_·7H2O 0.1 g, KCl 0.2 g, yeast extract 0.5 g, MnSO_4_ 2 mg, FeSO_4_·7H_2_O 2 mg, Bromphenol blue 25 mg, agar 20 g and sterilized distilled water (sdH_2_O) 1L) for 7 days at 30 °C. | Clear halo were observed around bacterial colonies | Cui et al., 2019 | + |
|  | Potassium solubilization | Bacteria was grown in Potassium feldspar (PF) solid medium (Sucrose 10 g, MgSO_4_·7H_2_O 0.5 g, (NH_4_)_2_SO_4_ 0.2 g, NaCl 0.1 g, CaCO_3_ 0.1 g, Potassium feldspar 5.0 g, Bromophenol blue 25 mg, agar 20 g and sdH_2_O1 L, pH 7.2) for 7 days at 30 °C. | Clear halo were observed around bacterial colonies | Cui et al., 2019 | + |
|  | Nitrogen fixation | Bacteria was grown in nitrogen-free Ashby medium (glucose 5 g, mannitol 5 g, CaCl_2_·2H_2_O 0.1 g, MgSO_4_·7H_2_O 0.1 g, Na_2_MoO_4_·2H_2_O 5 mg, K_2_HPO_4_ 0.9 g, KH_2_PO_4_ 0.1 g, FeSO_4_·7H_2_O 0.01 g, CaCO_3_ 5 g ,15 g agar in 1L sdH_2_O, pH 7.3) for 7 days at 28 °C. | Clear halo were observed around bacterial colonies | Cui et al., 2019 | + |
|  | Ferric siderophore production (Sid) | Bacteria was grown in Chrome azurol S agar medium (Shandong, China) and incubated at 30 °C for 48-72 h. | Development of a yellow to orange halo around the bacterial growth. | Schwyn and Neilands,  1987 | + |
|  | Indole-3-acetic-acid (IAA) production | Aliquots of 20 μL of an overnight grown bacterial culture were used to inoculate 5 mL TSB (Tryptic Soy Broth) without and with tryptophan (200 μg mL^−1^) and incubated at 30 °C for 24 h. | Overnight cultures were centrifuged and 1 mL supernatant was mixed with 4 mL Salkowski's reagent, and incubated for 20 min at room temperature before the absorbance was measured at 535 nm. | Rashid et al., 2012 | Without Tryptophan: 12.92  With Tryptophan: 31.01 |
|  | Ammonia production | Bacteria was inoculated into a test tube that contained 10 mL of 4 % peptone broth in a liquid medium, then cultured at 28 °C for 7 days. | The addition of Nesseler’s reagent, development of yellow to brown color. | Dixit et al., 2015 | + |
|  | Amylase production | Bacteria was grown in Difco Nutrient Agar (20.0 g soluble starch, 0.5 g KCl, 2.0 g NaNO_3_, 1.0 g K_3_PO_4_, 0.5 g MgSO_4_·7H_2_O, 5.0 g NaCl, and 18.0 g agar in 1L sdH_2_O, pH 6) for 3-5 days at 28 °C. | The plates were flooded with an iodine solution and a yellow zone around a colony in an otherwise blue medium. | Hankin and Anagnostakis, 1975 | + |
|  | Hydrogen cyanide (HCN) production | Bacteria was streaked on the TSA medium with 4.4 g glycine. Whatman no.1 filter paper soaked in a 2 % sodium carbonate in 0.5 % picric acid solution was placed on top of streaked plates and incubated at 30 °C for 4 days. | Development of orange to red color. | Ahmad et al., 2008 | - |
|  | ACC deaminase enzyme production | The absorbance of the bacteria extracts in the presence of assay reagent, with ACC (the substrate) and without ACC was measured by spectrophotometer (Shimadzu, UV‒2600, Japan) at 540 nm. | The amount of α-ketobutyrate produced was calculated by preparing its standard curve ranging between 0.1 and 1.0 µmoL. | Penrose and Glick, 2003 | 0.633803 |
| Cell wall degrading enzyme activity | Cellulose activity | Bacteria was inoculated on the cellulose-Congo red agar and incubated for 7 days at 30 °C. | Clear halo were observed around bacterial colonies. | Hendricks et al., 1995 | + |
|  | Pectinase activity | Bacteria was spot-inoculated on medium was composed of citrus pectin 10 g, (NH_4_)_2_SO_4_ 1.4 g, K_2_HPO_4_ 6 g, KH_2_PO_4_ 2 g, MgSO_4_ 0.1 g, and 20.0 g agar in 1L sdH_2_O, pH 6) for 24 h at 37 °C. | The plates were observed for clear zones around the colonies after flooding with 1 % iodine solution. | Rehman et al., 2015 | + |
|  | β 1,3-glucanase activity | Bacteria was inoculated on 1/2 R2A agar media (pachyman 0.2 %, peptone 0.1 %, Yeast extract 0.01 %, K_2_HPO_4_ 0.1 %, MgSO_4_ 0.05 %, NH_4_NO_3_ 0.2 %, congo red 0.005 %, and 20.0 g agar in 1L sdH_2_O, pH 7) for 48 h at 30 °C. | The positive bacteria formed a clear halo around the colony. | Hong and Meng, 2003 | + |
|  | Chitosanase activity | Bacteria was inoculated on chitosanase-detection agar plate (CDA plate) ( A 1 litre medium for CDA-plate preparation was prepared by mixing 10 g of chitosan (pre-dissolved in 200 mL of 1% acetic acid), 20 g agar and M9 medium containing the following salts: Na_2_HPO_4_ 1.3 g, KH_2_PO_4_ 3.0 g, NaCl 0.5 g, NH_4_Cl 1.0 g, MgSO_4_ 0.24 g and CaCl_2_ 0.01 g. The final pH was adjusted to 6.5 with NaOH) for 4 d at 30 °C. | The positive bacteria formed a clear halo around the colony. | Cheng and Li, 2000 | + |
|  | Protease activity | Bacteria was inoculated on LB supplemented with 2 % skimmed milk agar medium. | Clear halo were observed around bacterial colonies. | Adinarayana et al., 2003 | + |
|  | Chitinolytic activity | Chitinase medium was prepared by using ½ strength TSA supplemented with 0.6 % w/v colloidal chitin. Bacteria were spot inoculated on medium and incubated at 30 °C for 5 days. | Clear halo were observed around bacterial colonies | Bibi et al., 2012 | - |
| Amino acids | | Amino acids were pre-column derivatized with PITC and were separated and quantified under the optimum condition (running buffer 30 mM phosphate and 3 mM β-CD at pH 7.0; voltage of 20 kV) by Biochrom 30+ amino acid analyzer (Biochrom, U.K) attached to a PA800 high performance capillary electrophoresis (HPCE) system equipped with an on-column ultra violet detector system (Genstech Biotechnology Co., Ltd, Shanghai, China) refer to the method of Ren et al. (2012). The capillaries were Bare Fused-Silica Capillary-50 μm ID, 375 μm OD, 67 cm (pkg of 3) (Shkmsw Biotechnology Co., Ltd, Shanghai, China). | Individual amino acid concentrations were calculated by comparing the specific amino acid peak area to a standard curve for that amino acid. | Ren et al., 2012 | + |
| Phytohormones | | *B. licheniformis* XNRB-3 was grown in optimized liquid fermentation medium for seven days. After seven days, the pure culture filtrate in which *B. licheniformis* XNRB-3 was grown was supplemented with [D5]-IAA, [2H2] GA, [(±)-3,5,5,7,7,7-d6]-ABA] as an internal standard, extracted, and subjected to GC-MS/SIM for determination and quantification. Further, the GC/MS used for quantification was equipped with a HP-5 capillary column HP-5 (30 m length, 0.25 mm ID, 0.25 μm film, 325 ℃ maximum temperature) and used He (99.999 %,) was as the carrier gas with a head pressure of 30 kPa, an injector temperature of 200 °C, and an ionizing voltage of 70 eV. | Phytohormones concentrations were calculated from ratios of peak area of sample to a corresponding internal standard. | Shahzad et al., 2019 | + |
| Microtiter plate biofilm formation assay | | In short, polystyrene microtiter plates (Greiner Bio-one, Germany) were filled with 200 μL LB liquid medium and six wells were inoculated with 1.5 % strain XNRB-3 overnight cultures. The plates were incubated at 37 °C. After incubation for either 24 h, wells were gently washed three times with 200 μL of phosphate-buffered saline, and subsequently biofilm cells were stained with 200 μL of 0.1 % (wt/vol) crystal violet for 30 min. After these 30 min, the wells were washed twice with 200 μL sterile deionized water to remove unbound crystal violet. The remaining crystal violet was dissolved in 200 μL 96 % ethanol, and the absorbance was measured at 595 nm. | Strains with optical density at 595 nm (OD595) values of 0.4, which is about two times the background signal, or higher were considered positive for biofilm formation. | Djordjevic et al., 2002 | + |

Note: −, negative; +, positive. ACC deaminase activity (µM of α-ketobutyrate/mg of protein/h) by using this standard curve according to the following equation: Y = 0.0071X+0.1108, R^2^ = 0.9998. Indole acetic acid production (µg/ml) by using this standard curve according to the following equation: Y = 0.0387X+0.2002, R^2^ = 0.9997.

**Table S7:** Absorbance value of 10 mmol L^-1^ liquid Phlorizin medium at different wavelengths.

| Wavelength (nm) | OD value | Wavelength (nm) | OD value | Wavelength (nm) | OD value |
| --- | --- | --- | --- | --- | --- |
| 260 | 0.398 | 274 | 0.992 | 288 | 1.185 |
| 262 | 0.482 | 276 | 1.085 | 290 | 1.177 |
| 264 | 0.547 | 278 | 1.161 | 292 | 1.174 |
| 266 | 0.627 | 280 | 1.238 | 294 | 0.997 |
| 268 | 0.712 | 282 | 1.218 | 296 | 0.981 |
| 270 | 0.804 | 284 | 1.207 | 298 | 0.964 |
| 272 | 0.897 | 286 | 1.196 | 300 | 0.962 |

**Table S8:** Disease severity scoring standard and calculation formula.

| Criteria | Standard | References |
| --- | --- | --- |
| Scoring of wilting symptoms | 0 = healthy plant or plant without symptoms | Azabou et al., 2020 |
|  | 1 = 1-33 % of plant tissue affected by chlorosis, leaf and shoot necrosis, or defoliation |  |
|  | 2 = 34-66 % affected tissue |  |
|  | 3 = 67-100 % affected tissue |  |
|  | 4 = dead plant |  |
| The area under the disease progress curve (AUDPC) | AUDPC = [(t/2×(S2+2×S3+···+ 2Si−1+ Si))/4×n]×100, where t is the interval between observations in days, Si is the final mean severity (disease index), 4 is the maximum disease rating, and n is the number of observations. | López-Escudero et al., 2004 |
| The final mean severity of symptoms (FMS) | FMS=∑(Ni×Xi)/ni, where Ni is the number of plants with symptoms, Xi is the value of the symptom score, and ni is the number of diseased plants. | Cachinero et al., 2002 |
| Disease intensity (DI) | DI (%) =100×∑(Ni×Xi)/(15×4), where Ni is the number of plants with symptoms, Xi is the value of the symptom score, 15 is the total number of plants, and 4 is the maximum disease rating (based on the wilting symptoms). | Cachinero et al., 2002 |
| The relative control effect (%) | The relative control effect (%) = (DI_CK_ -DI_T_)/DI_CK_×100. | Cachinero et al., 2002 |
| Disease incidence (%) | Disease incidence (%) = Number of diseased plants/Total number of plants×100. | Wu et al., 2019 |

**Table S9:** Pure compounds purchased from reagent companies.

| Number | Compound Name | Chemical structure | Product properties | Purity | Prostitution |
| --- | --- | --- | --- | --- | --- |
| 1 | Harmaline |  | 25 mg | >98.0 % (HPLC) | Shanghai McLin Biochemical Technology Co., Ltd. |
| 2 | CH_3_C(O)CH_2_CH_2_OH |  | 25 g | 95 % | Shanghai McLin Biochemical Technology Co., Ltd. |
| 3 | Rhodanine |  | 25 g | AR | Shanghai Yuanye Biotechnology Co., Ltd. |
| 4 | Triethyl citrate |  | 25 g | 99 % | Shanghai Yuanye Biotechnology Co., Ltd. |
| 5 | Benzeneacetic acid, 4-hydroxy-, methyl ester |  | 25 g | 99 % | Shanghai Yuanye Biotechnology Co., Ltd. |
| 6 | Duroquinone |  | 1 g | 98 % | Shanghai McLin Biochemical Technology Co., Ltd. |
| 7 | 2-Coumaranone |  | 1 g | 98 % | Shanghai McLin Biochemical Technology Co., Ltd. |
| 8 | 2,3-Butanediol |  | 5 mL | 98 % | Shanghai McLin Biochemical Technology Co., Ltd. |
| 9 | 1,2-Benzenedicarboxylic acid, bis(1-methylethyl) ester |  | 5 mL | 98 % | Shanghai McLin Biochemical Technology Co., Ltd. |
| 10 | 2,4-Di-tert-butylphenol |  | 100 mg | > 99 % | Shanghai Yuanye Biotechnology Co., Ltd. |
| 11 | Butanedioic acid, monomethyl ester |  | 5 g | 98 % | Shanghai McLin Biochemical Technology Co., Ltd. |
| 12 | alpha-Bisabolol |  | 5 g | 90 % | Shanghai McLin Biochemical Technology Co., Ltd. |
| 13 | Acetoin |  | 25 g | 97 % | Shanghai McLin Biochemical Technology Co., Ltd. |
| 14 | Dibutyl phthalate |  | 500 mL | > 98.5 % (GC) | Shanghai McLin Biochemical Technology Co., Ltd. |
| 15 | 3-Nonen-2-one |  | 5 mL | ≥ 96 % | Shanghai McLin Biochemical Technology Co., Ltd. |
| 16 | Benzoic acid, 3,4-dimethyl-, methyl ester |  | 5 g | 97 % | Beijing JandK Scientific Technology Co., Ltd. |

**Table S10:**Properties of the carriers used in this study.

| Carrier | | | pH | EC  (ms cm^-1^) | OM (%) | TN (%) | K_2_O (%) | P_2_O_5_ (%) | Source |
| --- | --- | --- | --- | --- | --- | --- | --- | --- | --- |
| Soil materials | Peat1 | | 4.77 | 0.45 | 35.33 | 1.64 | 6.63 | 1.36 | Purchased in Shanghai, China through Taobao |
|  | Peat2 | | 6.36 | 0.55 | 38.72 | 1.03 | 6.84 | 1.58 | Purchased in Hebei, China through Taobao |
|  | Diatomite earths (inorganic soils) | | 7.55 | 1.43 | 0.24 | 0.47 | 4.35 | / | Purchased in Tianjin, China through Taobao |
| Organic materials | Composts | Herb residue compost | 7.72 | 2.13 | 41.6 | 2.28 | 7.2 | 3.14 | Purchased in Nanjing, China through Taobao |
|  |  | Cow dung compost | 7.00 | 3.46 | 36.82 | 1.37 | 7.8 | 4.85 | Purchased in Hebei, China through Taobao |
|  |  | Chicken manure compost | 8.30 | 4.53 | 25.79 | 1.16 | 8.66 | 2.17 | Purchased in Henan, China through Taobao |
|  |  | Earthworm manure compost | 7.81 | 0.88 | 11.66 | 1.35 | 3.79 | 7.09 | Purchased in Hebei, China through Taobao |
|  | Soybean meal | | 3.42 | 0.16 | 34.61 | 2.32 | 15.59 | 6.27 | Purchased in Anhui, China through Taobao |
|  | Pleurotus eryngii residue | | 7.45 | 2.27 | 27.20 | 3.16 | 10.9 | 2.94 | Purchased in Hebei, China through Taobao |
|  | Sawdust | | 6.92 | 1.33 | 45.40 | 0.31 | 1.57 | 0.48 | Purchased in Fujian, China through Taobao |
|  | Rice husk | | 7.31 | 3.26 | 37.09 | 0.77 | 6.22 | 1.76 | Purchased in Hebei, China through Taobao |
|  | Wheat straw | | 7.34 | 3.21 | 35.40 | 2.07 | 20.13 | 2.66 | Purchased in Hebei, China through Taobao |
|  | Corn stalks | | 8.21 | 1.94 | 44.44 | 1.98 | 17.02 | 4.61 | Purchased in Hebei, China through Taobao |
| Inert materials | Vermiculite | | 7.01 | 0.33 | 0.36 | 0.02 | 17.77 | 0.89 | Purchased in Shanghai, China through Taobao |
|  | Perlite | | 8.17 | 0.11 | 0.47 | 0.01 | / | 0.59 | Purchased in Shanghai, China through Taobao |

Note: EC, Electrolytic conductivity; OM, Organic matter; TN, Total nitrogen.

**Table S11:** The physical and chemical properties of the soil in the old apple orchard. Values are mean ± SD (n=3).

| Location | Nitrate nitrogen  (mg kg^-1^) | Ammonium nitrogen  (mg kg^-1^) | Available phosphorus  (mg kg^-1^) | Available potassium (mg kg^-1^) | Organic matter (%) | Soil pH | Soil moisture content (%) | Soil texture |
| --- | --- | --- | --- | --- | --- | --- | --- | --- |
| Manzhuang Town | 6.18±0.04 | 1.68±0.02 | 10.57±0.37 | 53.13±6.33 | 1.35±0.02 | 6.00±0.23 | 15.49±0.42 | Sandy loam |
| Wantou village, Laizhou city | 27.63±0.22 | 14.13±0.20 | 7.15±0.11 | 40.26±8.83 | 2.09±0.05 | 6.49±0.18 | 8.86±0.17 | Clay loam |

**Table S12:** Physiological and biochemical characteristics of strain XNRB-3.

| Text index | Results | Text index | Results |
| --- | --- | --- | --- |
| Hydrogen peroxide reaction | + | Arginine bihydrolysis reaction | ^_^ |
| Contact enzyme | + | Sucrose fermentation reaction | + |
| Starch hydrolysis enzyme | + | Glucose fermentation reaction | ^_^ |
| Nitrate reduction enzyme | + | Methyl red reaction | + |
| Indole enzyme | ^_^ | Voges-Proskauer reaction | + |
| Citrate enzyme | ^_^ | Urea enzyme reaction | + |
| Hydrogen sulfide reaction | + | Gelatin hydrolysis enzyme | + |

Note: +, positive reaction; ^_^, negative reaction. The test was repeated three times.

**Table S13:** The strain was identified by analyzing the similarity of the color development in a 96-well microtiter plate between XNRB-3 and standard strains in the kinetic database by Biolog Retrospect 2.0 Data Management Software. The most likely results were still listed, each of which shows three parameters: Probability (PROB), Similarity (SIM), and Distance (DIST). The results were considered to be more accurate when the SIM value > 0.5 and the DIST value < 5.00 are satisfied.

| Biolog ID DB |  | Biolog GEN Ⅲ DB.15G | |  |  |
| --- | --- | --- | --- | --- | --- |
| Result |  | Species ID: *Bacillus licheniformis* | | | |
| Comment |  |  |  |  |  |
| Notic |  |  |  |  |  |
| Rank | PROB | SIM | DIST | Qrganism Type | Species |
| ==>1 | 0.689 | 0.689 | 4.698 | GP-RodSB | *Bacillus licheniformis* |
| 2 | 0.155 | 0.155 | 4.768 | GP-RodSB | *Bacillus atrophaeus*/*subtilis* |
| 3 | 0.152 | 0.152 | 4.780 | GP-RodSB | *Bacillus subtilis ss subtilis* |
| 4 | 0.083 | 0.083 | 5.149 | GP-RodSB | *Bacillus subtilis ss spizizenii* |

**Table S14:** Utilization ability of strain XNRB-3 on 94 phenotypic tests. The Biolog GEN III MicroPlate analyzes a microorganism in 94 phenotypic tests: 71 carbon source utilization assays (columns 1-9) and 23 chemical sensitivity assays (columns 10-12). All wells visually resembling the negative control well A-1(positive control well: A-10) well should be scored as “negative” (-) and all wells with a noticeable purple color (greater than well A-1 and A-10) should be scored as “positive” (+). Wells with extremely faint color, or with small purple flecks or clumps are best scored as “borderline” (-/+).

| Columns | Nutrient matrix | Reaction type | Columns | Nutrient matrix | Reaction type | Columns | Nutrient matrix | Reaction type |
| --- | --- | --- | --- | --- | --- | --- | --- | --- |
| A1 | Negative Control | - | C9 | Inosine | -/+ | F5 | D-Glucuronic Acid | + |
| A2 | Dextrin | + | C10 | 1 % Sodium Lactate | + | F6 | Glucuronamide | + |
| A3 | D-Maltose | + | C11 | Fusidic Acid | - | F7 | Mucic Acid | -/+ |
| A4 | D-Trehalose | + | C12 | D-Serine | - | F8 | Quinic acid | - |
| A5 | D-Cellobiose | + | D1 | D-Sorbitol | + | F9 | D-Saccharic Acid | -/+ |
| A6 | Gentiobiose | + | D2 | D-Mannitol | + | F10 | Vancomycin | - |
| A7 | Sucrose | + | D3 | D-Arabitol | - | F11 | Tetrazolium Violet | -/+ |
| A8 | Turanose | + | D4 | Myo-Inositol | + | F12 | Tetrazolium Blue | - |
| A9 | Stachyose | -/+ | D5 | Glycerol | + | G1 | p-Hydroxy  -phenylacetic Acid | - |
| A10 | Positive Control | + | D6 | Glycerol | - | G2 | Methyl pyruvate | + |
| A11 | pH6 | + | D7 | D-Fructose-6-Phosphate | -/+ | G3 | D-Lactic Acid Methyl Ester | - |
| A12 | pH5 | - | D8 | D-Aspartic Acid | -/+ | G4 | L-Lactic Acid | + |
| B1 | D-Raffinose | -/+ | D9 | D-Serine | - | G5 | Citric Acid | -/+ |
| B2 | α-D-Lactose | - | D10 | Troleandomycin | - | G6 | α-Keto-glutaric Acid | - |
| B3 | D-Melibiose | -/+ | D11 | Rifamycin SV | - | G7 | D-Malic Acid | - |
| B4 | β-Methyl-D-Glucoside | + | D12 | Minocycline | - | G8 | L-Malic Acid | + |
| B5 | Salicin | + | E1 | Gelatin | + | G9 | Bromosuccinic Acid | -/+ |
| B6 | N-Acetyl-D-Glucosamine | + | E2 | Glycyl-L-Proline | -/+ | G10 | Nalidixic acid | - |
| B7 | N-Acetyl-β-D-Mannosamine | - | E3 | D-Alanine | -/+ | G11 | Lithium Chloride | + |
| B8 | N-Acetyl-D-Galactosamine | - | E4 | L-Arginine | -/+ | G12 | Potassium Tellurite | + |
| B9 | N-AcetylNeuraminic acid | - | E5 | L-Aspartic Acid | -/+ | H1 | Tween 40 | -/+ |
| B10 | 1 % NaCl | + | E6 | L-Glutamic Acid | -/+ | H2 | γ-Amino-Butyric Acid | - |
| B11 | 4 % NaCl | + | E7 | L-Histidine | - | H3 | α-Hydroxy-Butyric Acid | - |
| B12 | 8 % NaCl | + | E8 | L-Pyroglutamic Acid | - | H4 | β-Hydroxy-D,L-butyric Acid | - |
| C1 | α-D-Glucose | + | E9 | L-Serine | - | H5 | α-Keto-Butyric Acid | - |
| C2 | D-Mannose | + | E10 | Lincomycin | - | H6 | Acetoacetic Acid | -/+ |
| C3 | D-Fructose | + | E11 | Guanidine HCl | + | H7 | Propionic Acid | - |
| C4 | D-Galactose | -/+ | E12 | Niaproof 4 | - | H8 | Acetic Acid | -/+ |
| C5 | 3-Methyl-D-Glucose | - | F1 | Pectin | + | H9 | Formic Acid | - |
| C6 | L-Fucose | -/+ | F2 | Galacturonic acid | + | H10 | Aztreonam | + |
| C7 | D-Fucose | -/+ | F3 | D-Galactonic Acid Lactone | - | H11 | Sodium Butyrate | + |
| C8 | L-Rhamnose | -/+ | F4 | D-Gluconic Acid | + | H12 | Sodium Bromate | - |

**Table S15:** IAA, GA and ABA production levels by *B. licheniformis* XNRB-3.

| Hormones | Carrier gas flow rate | Source temperature | Temperature program | Content |
| --- | --- | --- | --- | --- |
| Gibberellin (GA) | 40 mL min^-1^ | 250 °C | 60 °C (1 min) → 15 °C min^-1^ → 200 °C (1 min) → 5 °C min^-1^ → 285 °C (5 min) | GA_4_ 1.89 ng mL^-1^  GA_20_ 1.65 ng mL^-1^ |
| Indol-3-ylacetic acid (IAA) | 60 mL min^-1^ | 230 °C | 70 °C (2 min) → 20 °C min^-1^ → 280 °C (5 min) → 5 °C min^-1^ | 15.275 μg mL^-1^ |
| Abscisic acid (ABA) | 40 mL min^-1^ | 250 °C | 60 °C (1 min) → 15 °C min^-1^ → 200 °C (1 min) → 5 °C min^-1^ → 250 °C → 10 °C min^-1^ → 280 °C | 0.157 ng mL^-1^ |

**Table S16:** Amino acid contents in culture medium using HPCE. Values in columns followed by the same letter are not significantly different according to Duncan test at *p* < 0.05. Values are mean ± SD (n=3).

| Amino acid | Content (mg mL^-1^) |
| --- | --- |
| Aspartic acid (Asp) | 194.76±3.03a |
| Threonine (Thr) | 19.17±0.99j |
| Serine (Ser) | 19.41±0.17ij |
| Glutamate (Glu) | 106.43±0.68b |
| Proline (Pro) | 37.83±0.75f |
| Glycine (Gly) | 22.49±1.18i |
| Alanine (Ala) | 41.74±0.84e |
| Cystine (Cys) | 19.57±0.49ij |
| Valine (Val) | 29.22±0.92h |
| Methionine (Met) | 32.90±0.42g |
| Isoleucine (Ile) | 20.54±0.30ij |
| Leucine (Leu) | 76.91±0.79c |
| Tyrosine (Tyr) | 60.16±0.78d |
| Phenylalanine (Phe) | 39.19±0.41ef |
| Histidine (His) | 20.14±1.13ij |
| Lysine (Lys) | 38.06±0.10f |
| Arginine (Arg) | 30.52±0.06gh |

**Table S17:** Inorganic salt combined fermentation condition L_25_ (5^3^) Orthogonal test design and results. The three factors were MgSO_4_, KH_2_PO_4_, KCl, and the 5 level was 0.1, 0.5, 1.0, 1.5, and 2.0 g L^-1^.

| Number | Influence factors | | | OD600 |
| --- | --- | --- | --- | --- |
|  | K2HPO4 | MgSO4 | KCl |  |
| 1 | 1 | 1 | 1 | 0.799 |
| 2 | 1 | 2 | 2 | 0.808 |
| 3 | 1 | 3 | 3 | 0.842 |
| 4 | 1 | 4 | 4 | 0.711 |
| 5 | 1 | 5 | 5 | 0.706 |
| 6 | 2 | 1 | 2 | 0.753 |
| 7 | 2 | 2 | 3 | 0.784 |
| 8 | 2 | 3 | 4 | 0.859 |
| 9 | 2 | 4 | 5 | 0.794 |
| 10 | 2 | 5 | 1 | 0.724 |
| 11 | 3 | 1 | 3 | 0.813 |
| 12 | 3 | 2 | 4 | 0.806 |
| 13 | 3 | 3 | 5 | 0.886 |
| 14 | 3 | 4 | 1 | 0.824 |
| 15 | 3 | 5 | 2 | 0.795 |
| 16 | 4 | 1 | 4 | 0.809 |
| 17 | 4 | 2 | 5 | 0.814 |
| 18 | 4 | 3 | 1 | 0.884 |
| 19 | 4 | 4 | 2 | 0.867 |
| 20 | 4 | 5 | 3 | 0.824 |
| 21 | 5 | 1 | 5 | 0.827 |
| 22 | 5 | 2 | 1 | 0.831 |
| 23 | 5 | 3 | 2 | 0.884 |
| 24 | 5 | 4 | 3 | 0.854 |
| 25 | 5 | 5 | 4 | 0.795 |
| K1 | 0.773 | 0.8 | 0.812 |  |
| K2 | 0.783 | 0.809 | 0.821 |  |
| K3 | 0.825 | 0.871 | 0.823 |  |
| K4 | 0.84 | 0.81 | 0.796 |  |
| K5 | 0.838 | 0.769 | 0.805 |  |
| Range value | 0.067 | 0.102 | 0.027 |  |
| Best level | I4 | F3 | E3 |  |

**Table S18:** Factors and levels of Plackett-Burman experimental design.

| Code | Factors | Low level | High level |
| --- | --- | --- | --- |
|  |  | -1 | 1 |
| A | Temperature (℃) | 25 | 37 |
| B | Rotating speed (rpm) | 150 | 250 |
| C | Liquid volume (mL) | 50 | 150 |
| D | pH | 7 | 9.5 |
| E | KCl (g) | 0.2 | 2 |
| F | MgSO_4_ (g) | 0.2 | 2 |
| G | Sucrose (g) | 0.2 | 2 |
| H | Beef extract (g) | 5 | 25 |
| I | KH_2_PO_4_(g) | 1 | 20 |

**Table S19:** Experimental design and response values of Plackett-Burman.

| Number | A | B | C | D | E | F | G | H | I | OD_600_ |
| --- | --- | --- | --- | --- | --- | --- | --- | --- | --- | --- |
| 1 | 1 | 1 | -1 | 1 | -1 | -1 | -1 | 1 | -1 | 0.768 |
| 2 | 1 | -1 | -1 | -1 | 1 | -1 | 1 | 1 | -1 | 0.954 |
| 3 | 1 | -1 | 1 | 1 | -1 | -1 | 1 | -1 | -1 | 0.829 |
| 4 | 1 | 1 | -1 | -1 | -1 | 1 | 1 | -1 | 1 | 0.789 |
| 5 | 1 | 1 | 1 | -1 | 1 | -1 | -1 | -1 | -1 | 0.728 |
| 6 | -1 | 1 | -1 | 1 | 1 | -1 | 1 | -1 | 1 | 0.759 |
| 7 | 1 | -1 | -1 | 1 | 1 | 1 | -1 | -1 | 1 | 0.764 |
| 8 | 1 | 1 | 1 | 1 | 1 | 1 | 1 | 1 | 1 | 0.815 |
| 9 | 1 | -1 | 1 | -1 | -1 | 1 | -1 | 1 | 1 | 0.867 |
| 10 | -1 | 1 | 1 | 1 | -1 | 1 | -1 | -1 | -1 | 0.598 |
| 11 | -1 | -1 | -1 | -1 | -1 | -1 | -1 | -1 | 1 | 0.773 |
| 12 | -1 | 1 | 1 | -1 | -1 | -1 | 1 | 1 | 1 | 0.742 |
| 13 | -1 | -1 | -1 | 1 | -1 | 1 | 1 | 1 | -1 | 0.781 |
| 14 | -1 | 1 | -1 | -1 | 1 | 1 | -1 | 1 | -1 | 0.679 |
| 15 | -1 | -1 | 1 | -1 | 1 | 1 | 1 | -1 | -1 | 0.748 |
| 16 | -1 | -1 | 1 | 1 | 1 | -1 | -1 | 1 | 1 | 0.793 |

**Table S20:** Effect evaluations of each factor under Plackett-Burman test design.

| Factors | Effect | Coefficient | Standard error | T value | P value | Significance |
| --- | --- | --- | --- | --- | --- | --- |
|  |  |  |  |  |  |  |
| constant |  | 0.77416 | 0.0068 | 113.79 | 0 |  |
| A | 0.08017 | 0.04009 | 0.0068 | 5.89 | 0.001 | ** |
| B | -0.07882 | -0.03941 | 0.0068 | -5.79 | 0.001 | ** |
| C | -0.01833 | -0.00916 | 0.0068 | -1.35 | 0.227 |  |
| D | -0.02168 | -0.01084 | 0.0068 | -1.59 | 0.162 |  |
| E | 0.01168 | 0.00584 | 0.0068 | 0.86 | 0.424 |  |
| F | -0.03818 | -0.01909 | 0.0068 | -2.81 | 0.031 | * |
| G | 0.05582 | 0.02791 | 0.0068 | 4.1 | 0.006 | ** |
| H | 0.05132 | 0.02566 | 0.0068 | 3.77 | 0.009 | ** |
| I | 0.02718 | 0.01359 | 0.0068 | 2 | 0.093 |  |

Note: (1) R^2^=95.09 %, R^2^_Adj_= 87.73 %. (2) * and** represented significant difference at *p* < 0.05 and *p* < 0.01, respectively.

**Table S21:** Factors and levels of Box-Behnken experiment.

| Code | Factors | Low level factorial (-1) | Centre point (0) | High level factorial (+1) |
| --- | --- | --- | --- | --- |
| G | Sucrose (g) | 15 | 20 | 25 |
| H | Beef extract (g) | 5 | 10 | 15 |
| A | Temperature (℃) | 25 | 31 | 37 |
| B | Rotating speed (rpm) | 150 | 200 | 250 |

**Table S22:** Factors and levels of Box-Behnken experiment.

| Number | A | B | G | H | Inhibition zone diameter (mm) |
| --- | --- | --- | --- | --- | --- |
| 1 | 0 | 0 | 1 | 1 | 17.58 |
| 2 | 0 | 0 | 0 | 0 | 21.71 |
| 3 | 0 | -1 | 1 | 0 | 20.98 |
| 4 | -1 | 1 | 0 | 0 | 20.92 |
| 5 | 0 | 0 | 0 | 0 | 22.30 |
| 6 | -1 | 0 | -1 | 0 | 20.72 |
| 7 | 0 | 0 | 0 | 0 | 22.16 |
| 8 | 0 | 1 | 1 | 0 | 19.60 |
| 9 | 0 | 0 | 1 | -1 | 19.80 |
| 10 | 0 | 0 | 0 | 0 | 22.11 |
| 11 | -1 | 0 | 0 | -1 | 19.92 |
| 12 | 1 | -1 | 0 | 0 | 20.92 |
| 13 | 1 | 0 | 1 | 0 | 21.28 |
| 14 | 0 | -1 | 0 | -1 | 19.86 |
| 15 | 0 | -1 | 0 | 1 | 18.92 |
| 16 | 1 | 1 | 0 | 0 | 20.35 |
| 17 | 0 | 0 | -1 | -1 | 17.53 |
| 18 | 0 | 0 | -1 | 1 | 17.88 |
| 19 | 0 | 1 | -1 | 0 | 19.70 |
| 20 | -1 | 0 | 1 | 0 | 18.25 |
| 21 | 0 | 1 | 0 | 1 | 18.95 |
| 22 | 1 | 0 | -1 | 0 | 17.28 |
| 23 | 0 | 1 | 0 | -1 | 20.28 |
| 24 | -1 | 0 | 0 | 1 | 18.21 |
| 25 | -1 | -1 | 0 | 0 | 21.02 |
| 26 | 0 | -1 | -1 | 0 | 19.15 |
| 27 | 0 | 0 | 0 | 0 | 21.99 |
| 28 | 1 | 0 | 0 | 1 | 18.28 |
| 29 | 1 | 0 | 0 | -1 | 19.56 |

**Table S23:** Factors and levels of Box-Behnken experiment.

| Code | Factors | Low level factorial (-1) | Centre point (0) | High level factorial (+1) |
| --- | --- | --- | --- | --- |
| A | Inoculation amount (%) | 20 | 30 | 40 |
| B | pH | 6.5 | 7.5 | 8.5 |
| C | Temperature (℃) | 20 | 30 | 40 |
| D | Rotating speed (rpm) | 100 | 150 | 200 |

**Table S24:** Factors and levels of Box-Behnken experiment.

| Number | A | B | C | D | Population (E+08) |
| --- | --- | --- | --- | --- | --- |
| 1 | 0 | 0 | 0 | 0 | 8.71 |
| 2 | -1 | 0 | 1 | 0 | 8.26 |
| 3 | -1 | 1 | 0 | 0 | 8.2 |
| 4 | -1 | 0 | -1 | 0 | 6.64 |
| 5 | 1 | 0 | 0 | -1 | 6.36 |
| 6 | 1 | 0 | 0 | 1 | 7.31 |
| 7 | 1 | 1 | 0 | 0 | 6.28 |
| 8 | 0 | 0 | 0 | 0 | 8.87 |
| 9 | -1 | -1 | 0 | 0 | 4.36 |
| 10 | 0 | 1 | 0 | 1 | 8.09 |
| 11 | 0 | 0 | 0 | 0 | 8.45 |
| 12 | 0 | -1 | 1 | 0 | 4.61 |
| 13 | 1 | 0 | 1 | 0 | 7.12 |
| 14 | -1 | 0 | 0 | -1 | 7.52 |
| 15 | 0 | -1 | 0 | -1 | 5.37 |
| 16 | 0 | 0 | 0 | 0 | 8.78 |
| 17 | 0 | 0 | -1 | 1 | 7.26 |
| 18 | 0 | 1 | 1 | 0 | 8.52 |
| 19 | -1 | 0 | 0 | 1 | 7.89 |
| 20 | 1 | 0 | -1 | 0 | 6.12 |
| 21 | 0 | -1 | -1 | 0 | 4.46 |
| 22 | 0 | -1 | 0 | 1 | 5.08 |
| 23 | 0 | 0 | -1 | -1 | 6.65 |
| 24 | 1 | -1 | 0 | 0 | 4.52 |
| 25 | 0 | 1 | -1 | 0 | 5.92 |
| 26 | 0 | 0 | 1 | 1 | 8.34 |
| 27 | 0 | 0 | 0 | 0 | 8.77 |
| 28 | 0 | 1 | 0 | -1 | 6.52 |
| 29 | 0 | 0 | 1 | -1 | 8.32 |

**Table S25:** GC-MS identification result of extracellular metabolites.

| Number | Retention time (min) | Ingredient name | Area % | Molecular formula | Molecular weight | Retention index | CAS number |
| --- | --- | --- | --- | --- | --- | --- | --- |
| 1 | 11.867 | 2,3-Butanediol | 12.16 | C_4_H_10_O_2_ | 90 | 700 | 513 - 85 - 9 |
| 2 | 8.05 | CH_3_C(O)CH_2_CH_2_OH | 5.39 | C_4_H_8_O_2_ | 88 | 798 | 590 - 90 - 9 |
| 3 | 21.671 | Benzoic acid, 3,4-dimethyl-, methyl ester | 4.89 | C_10_H_12_O_2_ | 164 | 1287 | 38404 - 42 - 1 |
| 4 | 19.886 | 3-Nonen-2-one | 4.44 | C_9_H_16_O | 140 | 1060 | 14309 - 57 - 0 |
| 5 | 26.613 | alpha-Bisabolol | 3.06 | C_15_H_26_O | 222 | 1625 | 515 - 69 - 5 |
| 6 | 30.091 | Sesquicineole | 2.97 | C_15_H_26_O | 222 | 1541 | 90131 - 02 - 5 |
| 7 | 20.451 | 2-Coumaranone | 2.69 | C_8_H_6_O_2_ | 134 | 1272 | 553 - 86 - 6 |
| 8 | 34.219 | Dibutyl phthalate | 2.68 | C_16_H_22_O_4_ | 278 | 2037 | 84 - 74 - 2 |
| 9 | 24.934 | Benzeneacetic acid, 4-hydroxy-, methyl ester | 2.36 | C_9_H_10_O_3_ | 166 | 1380 | 14199 - 15 - 6 |
| 10 | 10.015 | Acetoin | 2.26 | C_4_H_8_O_2_ | 88 | 717 | 513 - 86 - 0 |
| 11 | 20.215 | Thiophene, 2,3-dihydro- | 2.17 | C_4_H_6_S | 86 | 723 | 1120 - 59 - 8 |
| 12 | 29.007 | Ledol | 1.78 | C_15_H_26_O | 222 | 1530 | 577 - 27 - 5 |
| 13 | 17.705 | Butanedioic acid, monomethyl ester | 1.77 | C_5_H_8_O_4_ | 132 | 1042 | 3878 - 55 - 5 |
| 14 | 18.66 | Hexestrol dimethyl ether | 1.37 | C_20_H_26_O_2_ | 298 | 2214 | 130 - 78 - 9 |
| 15 | 26.839 | (6Z,9Z,12Z,15Z)-Methyl octadeca-6,9,12,15-tetraenoate | 1.18 | C_19_H_30_O_2_ | 290 | 2109 | 73097 - 00 - 4 |
| 16 | 29.463 | (2E,4S,7E)-4-Isopropyl-1,7-dimethylcyclodeca-2,7-dienol | 1.15 | C_15_H_26_O | 222 | 1660 | 198991 - 79 - 6 |
| 17 | 19.777 | 4-Nonanol, 4-methyl- | 1.14 | C_10_H_22_O | 158 | 1107 | 23418 - 38 - 4 |
| 18 | 22.101 | 1,2-Benzenedicarboxylic acid | 1.14 | C_8_H_6_O_4_ | 166 | 1620 | 88 - 99 - 3 |
| 19 | 25.676 | Duroquinone | 1.14 | C_10_H_12_O_2_ | 164 | 1384 | 527 - 17 - 3 |
| 20 | 25.263 | 2,4-Di-tert-butylphenol | 1.12 | C_14_H_22_O | 206 | 1555 | 96 - 76 - 4 |
| 21 | 33.886 | Harmaline | 1.09 | C_13_H_14_N_2_O | 214 | 1912 | 304 - 21 - 2 |
| 22 | 28.626 | 1,4,5,8,9,10-Hexahydroanthracene | 1.07 | C_14_H_16_ | 184 | 1525 | 5910 - 28 - 1 |
| 23 | 15.996 | 1,2-Bis(trimethylsilyl)benzene | 1.06 | C_12_H_22_Si_2_ | 222 | 1124 | 17151 - 09 - 6 |
| 24 | 20.596 | Benzeneacetic acid | 1.02 | C_8_H_8_O_2_ | 136 | 1249 | 103 - 82 - 2 |
| 25 | 29.273 | Docosahexaenoic acid, 1,2,3-propanetriyl ester | 1.02 | C_69_H_98_O_6_ | 1022 | 7462 | 11094 - 59 - 0 |
| 26 | 16.554 | Decane, 1-chloro- | 0.99 | C_10_H_21_Cl | 176 | 1240 | 1002 - 69 - 3 |
| 27 | 21.417 | 2-Nonynoic acid, methyl ester | 0.98 | C_10_H_16_O_2_ | 168 | 1200 | 111 - 80 - 8 |
| 28 | 24.786 | 1-Phenyl-1-heptyne | 0.97 | C_13_H_16_ | 172 | 1407 | 14374 - 45 - 9 |
| 29 | 27.953 | Triethyl citrate | 0.97 | C_12_H_20_O_7_ | 276 | 1808 | 77 - 93 - 0 |
| 30 | 17.079 | p-Cresol | 0.91 | C_7_H_8_O | 108 | 1014 | 106 - 44 - 5 |
| 31 | 20.93 | Octadecane, 1-chloro- | 0.91 | C_18_H_37_Cl | 288 | 2036 | 3386 - 33 - 2 |
| 32 | 36.514 | 9-Octadecynoic acid, methyl ester | 0.91 | C_19_H_34_O_2_ | 294 | 2095 | 1120 - 32 - 7 |
| 33 | 26.023 | 4,8,12,16-Tetramethylheptadecan-4-olide | 0.79 | C_21_H_40_O_2_ | 324 | 2258 | 96168 - 15 - 9 |
| 34 | 16.253 | Pentanoic acid, 2-hydroxy-4-methyl-, methyl ester | 0.77 | C_7_H_14_O_3_ | 146 | 983 | 40348 - 72 - 9 |
| 35 | 28.321 | .beta.-Acorenol | 0.77 | C_15_H_26_O | 222 | 1598 | 28400 - 11 - 5 |
| 36 | 28.205 | 1,2-Benzenedicarboxylic acid, bis(1-methylethyl) ester | 0.75 | C_14_H_18_O_4_ | 250 | 1710 | 605 - 45 - 8 |
| 37 | 28.493 | Heptadecane | 0.73 | C_17_H_36_ | 240 | 1711 | 629 - 78 - 7 |
| 38 | 24.454 | 2,3,4,5-Tetramethylcyclopent-2-en-1-ol | 0.71 | C_9_H_16_O | 140 | 1071 | 82061 - 20 - 9 |
| 39 | 18.237 | 2(3H)-Furanone, 5-acetyldihydro- | 0.68 | C_6_H_8_O_3_ | 128 | 1122 | 29393 - 32 - 6 |
| 40 | 16.863 | Ethanone, 1-(2-methyl-1-cyclopenten-1-yl)- | 0.67 | C_8_H_12_O | 124 | 996 | 3168 - 90 - 9 |
| 41 | 13.327 | Rhodanine | 0.66 | C_3_H_3_NOS_2_ | 133 | 1212 | 141 - 84 - 4 |
| 42 | 18.012 | Phenylethyl Alcohol | 0.62 | C_8_H_10_O | 122 | 1136 | 1960-12-8 |

**Table S26:** The inhibition of plant fungal pathogens mycelia growth by pure compounds identified from *B. licheniformis* XNRB3. Values in columns followed by the same letter are not significantly different according to Duncan test at *p* < 0.05. Values are mean ± SD (n=3). -, No inhibitory effect on fungi. *, the diameter of the fungus cannot be measured.

| Number | Ingredient name | Concentration (μg L^-1^) | *F. proliferatum* | *F. verticillioides* | *F. oxysporum* | *F. solani* | *Rhizoctonia solani* | *Alternaria alternata* | *Albifimbria verrucaria* | *Aspergillus flavus* | *Penicillium brasilianum* | *Phytophthora* |
| --- | --- | --- | --- | --- | --- | --- | --- | --- | --- | --- | --- | --- |
| 1 | Harmaline | 10 | - | - | - | - | - | - | - | - | - | - |
|  |  | 100 | - | - | - | - | - | - | - | - | - | - |
|  |  | 500 | - | - | - | - | - | - | - | - | - | - |
|  |  | 1000 | - | - | - | - | - | - | - | - | - | - |
| 2 | CH_3_C(O)CH_2_CH_2_OH | 10 | - | - | - | - | - | - | - | - | - | - |
|  |  | 100 | - | - | - | - | - | - | - | - | - | - |
|  |  | 500 | - | - | - | - | - | - | - | - | - | - |
|  |  | 1000 | - | - | - | - | - | - | - | - | - | - |
| 3 | Rhodanine | 10 | - | - | - | - | - | - | - | - | - | - |
|  |  | 100 | - | - | - | - | - | - | - | - | - | - |
|  |  | 500 | - | - | - | - | - | - | - | - | - | - |
|  |  | 1000 | - | - | - | - | - | - | - | - | - | - |
| 4 | Triethyl citrate | 10 | - | - | - | - | - | - | - | - | - | - |
|  |  | 100 | - | - | - | - | - | - | - | - | - | - |
|  |  | 500 | - | - | - | - | - | - | - | - | - | - |
|  |  | 1000 | - | - | - | - | - | - | - | - | - | - |
| 5 | Benzeneacetic acid, 4-hydroxy-, methyl ester | 10 | - | - | - | - | - | - | - | - | - | - |
|  |  | 100 | - | - | - | - | - | - | - | - | - | - |
|  |  | 500 | - | - | - | - | - | - | - | - | - | - |
|  |  | 1000 | - | - | - | - | - | - | - | - | - | - |
| 6 | Duroquinone | 10 | - | - | - | - | - | - | - | - | - | - |
|  |  | 100 | - | - | - | - | - | - | - | - | - | - |
|  |  | 500 | - | - | - | - | - | - | - | - | - | - |
|  |  | 1000 | - | - | - | - | - | - | - | - | - | - |
| 7 | 2-Coumaranone | 10 | - | - | - | - | - | - | - | - | - | - |
|  |  | 100 | - | - | - | - | - | - | - | - | - | - |
|  |  | 500 | - | - | - | - | - | - | - | - | - | - |
|  |  | 1000 | - | - | - | - | - | - | - | - | - | - |
| 8 | 2,3-Butanediol | 10 | 7.03±0.01a | 7.02±0.00a | - | 7.01±0.00a | - | 7.01±0.01a | 3.70±0.00a | - | 2.60±0.00a | 5.31±0.00a |
|  |  | 100 | 7.02±0.00a | 7.01±0.00b | - | 6.81±0.00b | - | 7.01±0.00a | 3.60±0.00b | - | 1.31±0.00b | 5.30±0.00a |
|  |  | 500 | 7.01±0.01a | 6.9±0.01c | - | 6.80±0.00bc | - | 7.00±0.00a | 3.41±0.00c | - | 1.30±0.00b | 5.00±0.00b |
|  |  | 1000 | 6.80±0.00b | 6.70±0.00d | - | 6.80±0.00c | - | 7.00±0.00a | 2.31±0.00d | - | 1.20±0.00c | 4.40±0.00c |
| 9 | 2,4-Di-tert-butylphenol | 10 | 4.50±0.00a | 4.01±0.00a | 5.10±0.00a | 4.80±0.00a | 4.21±0.00a | 3.80±0.00a | 2.10±0.00a | 7.00±0.00a | * | 4.40±0.00a |
|  |  | 100 | 4.01±0.00b | 3.50±0.00b | 4.50±0.00b | 4.61±0.00b | 2.01±0.01b | 3.01±0.00b | 2.00±0.00b | 6.00±0.00a | * | 3.90±0.00b |
|  |  | 500 | 3.60±0.00c | 3.40±0.00c | 4.30±0.00c | 4.40±0.00c | 1.70±0.00c | 2.90±0.00c | 1.81±0.00c | 5.90±0.00a | * | 3.80±0.00c |
|  |  | 1000 | 2.30±0.00d | 3.10±0.00d | 3.50±0.00d | 4.10±0.00d | 1.50±0.00d | 2.60±0.00d | 1.10±0.00d | 5.80±0.00b | * | 2.10±0.00d |
| 10 | 1,2-Benzenedicarboxylic acid, bis(1-methylethyl) ester | 10 | 6.50±0.00a | 6.53±0.03a | 7.50±0.00a | 7.51±0.00a | 5.50±0.00a | 4.21±0.00a | 3.11±0.00a | 7.10±0.00a | 9.00±0.00a | 6.30±0.00a |
|  |  | 100 | 5.20±0.00b | 5.51±0.01b | 7.01±0.00b | 6.50±0.00b | 4.80±0.00b | 4.00±0.00b | 3.10±0.00a | 6.61±0.00b | 9.00±0.00a | 4.03±0.03b |
|  |  | 500 | 4.30±0.00c | 4.50±0.00c | 4.00±0.00c | 5.61±0.00c | 4.00±0.00c | 3.41±0.00c | 2.60±0.00b | 5.10±0.00c | 4.50±0.00b | 2.30±0.00c |
|  |  | 1000 | 2.00±0.00d | 3.10±0.00d | 2.80±0.00d | 5.20±0.00d | 2.30±0.00d | 3.20±0.00d | 1.70±0.00c | 2.00±0.00d | 3.00±0.00c | 1.10±0.00d |
| 11 | Butanedioic acid, monomethyl ester | 10 | 7.00±0.00a | 7.61±0.00a | 7.80±0.00a | 7.50±0.00a | - | 8.01±0.00a | 3.50±0.01a | 7.81±0.00a | - | 6.60±0.00a |
|  |  | 100 | 6.20±0.00b | 7.50±0.00b | 7.80±0.00b | 7.20±0.00a | - | 7.20±0.00b | 3.50±0.00a | 7.30±0.00b | - | 6.30±0.00b |
|  |  | 500 | 6.00±0.00c | 7.30±0.00c | 7.51±0.00c | 6.53±0.00b | - | 6.80±0.00c | 3.40±0.00a | 7.30±0.00b | - | 6.03±0.03c |
|  |  | 1000 | 5.83±0.03d | 6.50±0.00d | 7.00±0.00d | 7.00±0.00c | - | 6.61±0.03d | 3.00±0.00b | 7.30±0.00b | - | 5.00±0.00d |
| 12 | alpha-Bisabolol | 10 | 6.00±0.00a | 7.31±0.00a | 7.01±0.00a | 7.00±0.00a | 4.57±0.07a | 5.10±0.00a | 3.50±0.00a | 5.40±0.00a | * | 5.93±0.03a |
|  |  | 100 | 4.70±0.00b | 4.20±0.00b | 5.00±0.00b | 5.70±0.00b | 4.00±0.00b | 4.20±0.00b | 2.50±0.00b | 4.50±0.00b | * | 3.60±0.00b |
|  |  | 500 | 4.27±0.03c | 3.50±0.00c | 3.40±0.00c | 4.90±0.00c | 3.10±0.00c | 3.30±0.00c | 1.80±0.00c | 2.80±0.00c | * | 2.70±0.00c |
|  |  | 1000 | 2.00±0.00d | 3.20±0.00d | 2.83±0.03d | 4.20±0.00d | 2.93±0.03d | 2.93±0.03d | 1.50±0.00d | 2.5±0.00d | * | 2.10±0.00d |
| 13 | Acetoin | 10 | 7.20±0.00a | 7.10±0.00a | 9.00±0.00a | 7.40±0.00a | - | 7.80±0.00a | 3.80±0.00a | 7.50±0.00a | * | 6.20±0.00a |
|  |  | 100 | 6.80±0.00b | 6.80±0.00b | 7.50±0.00b | 7.00±0.00b | - | 6.70±0.00b | 3.70±0.10a | 7.10±0.00b | * | 5.80±0.00b |
|  |  | 500 | 6.70±0.00c | 6.80±0.00b | 6.61±0.00c | 6.60±0.00c | - | 6.30±0.00c | 3.70±0.00a | 6.90±0.03c | * | 5.50±0.00c |
|  |  | 1000 | 6.40±0.00d | 6.70±0.00c | 6.30±0.00d | 6.3±0.00d | - | 6.00±0.00d | 3.50±0.00b | 5.93±0.00d | * | 5.00±0.00d |
| 14 | Dibutyl phthalate | 10 | 6.31±0.01a | 6.41±0.00a | 7.01±0.00a | 4.71±0.00a | 7.07±0.07a | 4.50±0.00a | 3.27±0.07a | * | * | 7.00±0.00a |
|  |  | 100 | 6.31±0.00a | 6.20±0.00b | 6.90±0.00b | 4.70±0.00a | 6.50±0.00b | 4.40±0.00b | 3.20±0.00b | * | * | 6.70±0.00b |
|  |  | 500 | 6.30±0.00a | 5.90±0.00c | 6.80±0.00c | 4.70±0.00a | 5.00±0.15c | 4.30±0.00c | 3.20±0.00c | * | * | 6.30±0.00c |
|  |  | 1000 | 6.30±0.00a | 5.60±0.00d | 6.40±0.00d | 4.10±0.00b | 4.10±0.26d | 4.20±0.00d | 3.00±0.00d | * | * | 5.00±0.00d |
| 15 | 3-Nonen-2-one | 10 | 6.80±0.00a | 7.30±0.00a | 7.50±0.00a | 6.50±0.00a | - | 7.10±0.00a | 3.77±0.07a | - | - | 5.80±0.00a |
|  |  | 100 | 6.40±0.00b | 7.30±0.00a | 7.50±0.00a | 6.50±0.00a | - | 6.80±0.00b | 3.60±0.10ab | - | - | 5.50±0.00a |
|  |  | 500 | 4.60±0.00c | 6.80±0.00b | 7.00±0.00b | 6.50±0.00a | - | 6.50±0.00c | 3.50±0.00b | - | - | 3.55±1.67a |
|  |  | 1000 | 2.70±0.00d | 6.80±0.00b | 7.00±0.00b | 6.00±0.00b | - | 6.50±0.00c | 3.40±0.00b | - | - | 4.50±0.00a |
| 16 | Benzoic acid, 3,4-dimethyl-, methyl ester | 10 | 7.00±0.00a | 7.80±0.00a | 6.00±0.00a | 7.20±0.00a | - | 7.30±0.00a | 3.00±0.00a | - | - | 5.80±0.00a |
|  |  | 100 | 6.80±0.00b | 7.30±0.00b | 6.01±0.01a | 6.80±0.00bc | - | 6.70±0.00b | 2.90±0.00b | - | - | 4.60±0.00b |
|  |  | 500 | 6.70±0.00c | 7.00±0.00c | 6.00±0.00a | 6.30±0.00c | - | 6.00±0.00c | 2.80±0.00c | - | - | 4.40±0.00c |
|  |  | 1000 | 6.50±0.00d | 6.50±0.00d | 6.00±0.00a | 6.00±0.00d | - | 5.80±0.00d | 2.60±0.00d | - | - | 4.00±0.00d |

**Table S27.**Degradation of Cinnamic acid (CA), Ferulic acid (FA), Benzoic acid (BA), and P-hydroxybenzoic acid (PHBA) by strains after 60 h of culture. Values in columns followed by the same letter are not significantly different according to Duncan test at *p* < 0.05. Values are mean ± SD (n=3).

| Treatment | Time | CA | | PHBA | | BA | | FA | |
| --- | --- | --- | --- | --- | --- | --- | --- | --- | --- |
|  |  | OD_600_ | Degradation rate (%) | OD_600_ | Degradation rate (%) | OD_600_ | Degradation rate (%) | OD_600_ | Degradation rate (%) |
| CK | | 0·000±0.000f | 0.00±0.00f | 0·000±0.000f | 0.00±0.00f | 0·000±0.000f | 0.00±0.00f | 0·000±0.000f | 0.00±0.00f |
| Strain XNRB-3 | 12 h | 0.299±0.001e | 12.96±0.41e | 0.355±0.002e | 21.31±0.64e | 0.203±0.001e | 11.72±1.10e | 0.387±0.002e | 15.73±0.37e |
|  | 24 h | 0.365±0.002d | 16.17±0.68d | 0.410±0.003d | 30.60±0.67d | 0.286±0.001d | 15.49±0.24d | 0.474±0.002d | 22.79±0.94d |
|  | 36 h | 0.440±0.001c | 30.93±0.61c | 0.538±0.002c | 39.93±0.48c | 0.370±0.001c | 25.69±0.39c | 0.555±0.002c | 34.69±0.29c |
|  | 48 h | 0.572±0.001b | 43.28±0.64b | 0.650±0.001b | 56.16±0.81b | 0.448±0.001b | 36.46±0.84b | 0.632±0.001b | 52.29±0.29b |
|  | 60 h | 0.613±0.001a | 56.25±0.67a | 0.725±0.002a | 69.20±0.13a | 0.512±0.001a | 45.65±0.55a | 0.702±0.002a | 62.59±0.61a |

**Table S28:** The functional diversity of soil microbial communities by rhizosphere soil samples under different treatments using Biolog Eco plates, as reflected by the Shannon richness, Shannon evenness, Simpson index, and Mc Intosh index. Values are mean ± SD (n=3).

| Treatment | Sampling time | Shannon richness | Shannon evenness | Simpson index | Mc Intosh index |
| --- | --- | --- | --- | --- | --- |
| CK1 | October, 2020 | 3.22±0.04 | 0.97±0.02 | 0.96±0.00 | 9.86±1.17 |
|  | October, 2021 | 3.14±0.03 | 0.97±0.01 | 0.95±0.00 | 9.67±0.48 |
| CK2 | October, 2020 | 3.10±0.04 | 1.00±0.01 | 0.95±0.00 | 6.68±0.10 |
|  | October, 2021 | 3.21±0.04 | 0.99±0.00 | 0.95±0.00 | 6.39±0.35 |
| T1 | October, 2020 | 3.27±0.03 | 0.99±0.01 | 0.96±0.00 | 10.18±0.35 |
|  | October, 2021 | 3.29±0.01 | 0.98±0.00 | 0.96±0.00 | 10.43±0.50 |
| T2 | October, 2020 | 3.27±0.02 | 0.98±0.00 | 0.96±0.00 | 10.78±0.79 |
|  | October, 2021 | 3.30±0.02 | 0.99±0.01 | 0.96±0.00 | 12.49±1.11 |

**Table S29:** Effect of different treatments on Margalef Index, Mc Intosh Index, Brillouin Index, Simpson Index and Shannon Index based on the T-RFLP data in October 2021. Values in columns followed by the same letter are not significantly different according to Duncan test at *p* < 0.05. Values are mean ± SD (n=3). The Margalef index reflects the abundance of soil microbial communities; the Mc intosh index reflects the number of different types of carbon sources utilized, which can be used to distinguish different carbon source utilization levels; and the Brillouin, Simpson, and Shannon Index reflects the diversity of soil microbial communities.

| Microorganism | Treatment | Margalef Index | Shannon Index | Simpson Index | Mc Intosh Index | Brillouin Index |
| --- | --- | --- | --- | --- | --- | --- |
| Fungi | CK1 | 6.91±0.24b | 2.94±0.03c | 0.076±0.002a | 31.27±1.14a | 2.55±0.04a |
|  | CK2 | 5.76±0.21c | 2.87±0.03c | 0.054±0.002b | 13.08±0.05c | 2.34±0.02b |
|  | T1 | 7.68±0.29b | 3.12±0.05b | 0.049±0.004b | 17.51±0.49b | 2.61±0.06a |
|  | T2 | 9.07±0.23a | 3.31±0.04a | 0.027±0.001c | 11.28±0.13c | 2.59±0.05a |
| Bacterial | CK1 | 4.19±0.34b | 2.69±0.08b | 0.092±0.009b | 40.37±4.36b | 2.44±0.08b |
|  | CK2 | 6.33±0.55a | 3.23±0.10a | 0.042±0.006c | 24.25±1.26c | 2.84±0.07a |
|  | T1 | 5.01±0.22ab | 2.69±0.05b | 0.101±0.004b | 37.37±1.75b | 2.39±0.05b |
|  | T2 | 5.12±0.60ab | 2.30±0.11c | 0.172±0.016a | 54.60±1.99a | 2.07±0.09c |

# References

Adinarayana, K., Ellaiah, P., and Prasad, D. S. (2003). Purification and partial characterization of thermostable serine alkaline protease from a newly isolated *Bacillus subtilis* PE-11. *Aaps Pharmscitech*. *4*(4), 440-448.

Ahmad, F., Ahmad, I., and Khan, M. S. (2008). Screening of free-living rhizospheric bacteria for their multiple plant growth promoting activities. *Microbiological research*. *163*(2), 173-181.

Bibi, F., Yasir, M., Song, G. C., Lee, S. Y., and Chung, Y. R. (2012). Diversity and characterization of endophytic bacteria associated with tidal flat plants and their antagonistic effects on oomycetous plant pathogens. *The Plant Pathology Journal*. *28*(1), 20-31.

Cachinero, J. M., Hervas, A., Jiménez‐Díaz, R. M., and Tena, M. (2002). Plant defence reactions against Fusarium wilt in chickpea induced by incompatible race of *Fusarium oxysporum* f. sp. *ciceris* and nonhost isolates of *F. oxysporum*. *Plant pathology*. *51*(6), 765-776.

Cao, Y., Xu, Z., Ling, N., Yuan, Y., Yang, X., Chen, L., et al. (2012). Isolation and identification of lipopeptides produced by *B. subtilis* SQR 9 for suppressing Fusarium wilt of cucumber. *Scientia horticulturae*. *135*, 32-39.

Cheng, C. Y., and Li, Y. K. (2000). An *Aspergillus* chitosanase with potential for large‐scale preparation of chitosan oligosaccharides. *Biotechnology and Applied Biochemistry*. *32*(3), 197-203.

Chun, J., and Bae, K. S. (2000). Phylogenetic analysis of *Bacillus subtilis* and related taxa based on partial *gyrA* gene sequences. *Antonie van Leeuwenhoek*. *78*(2), 123-127.

Cui, W., He, P., Munir, S., He, P., Li, X., Li, Y., et al. (2019). Efficacy of plant growth promoting bacteria *Bacillus amyloliquefaciens* B9601-Y2 for biocontrol of southern corn leaf blight. *Biological Control*. *139*, 104080.

Dixit, R., Singh, R. B., and Singh, H. B. (2015). Screening of antagonistic potential and plant growth promotion activities of *Trichoderma* spp. and *fluorescent Pseudomonas* spp. isolates against *Sclerotinia sclerotiorum* causing stem rot of French bean. *Legume Research-An International Journal*. *38*(3), 375-381.

Djordjevic, D., Wiedmann, M., and McLandsborough, L. A. (2002). Microtiter plate assay for assessment of *Listeria monocytogenes* biofilm formation. *Applied and environmental microbiology.* 68(6), 2950-2958.

Gardes, M., and Bruns, T. D. (1993). ITS primers with enhanced specificity for basidiomycetes‐application to the identification of mycorrhizae and rusts. *Molecular ecology*. *2*(2), 113-118.

Hankin, L., and Anagnostakis, S. L. (1975). The use of solid media for detection of enzyme production by fungi. *Mycologia*. *67*(3), 597-607.

Hendricks, C. W., Doyle, J. D., and Hugley, B. (1995). A new solid medium for enumerating cellulose-utilizing bacteria in soil. *Applied and environmental microbiology*. *61*(5), 2016-2019.

Hong, T. Y., and Meng, M. (2003). Biochemical characterization and antifungal activity of an endo-1, 3-β-glucanase of *Paenibacillus* sp. isolated from garden soil. *Applied microbiology and biotechnology*. *61*(5), 472-478.

Hsieh, F. C., Li, M. C., Lin, T. C., and Kao, S. S. (2004). Rapid detection and characterization of surfactin-producing *Bacillus subtilis* and closely related species based on PCR. *Current microbiology*. *49*(3), 186-191.

Hussein, W., and Fahim, S. A. M. E. H. (2017). Detection of synthetases genes involved in non ribosomal lipopeptides (NRLPs) biosynthesis from *Bacillus* species by bioinformatics and PCR degenerated primers and estimation of their production. *Int. J. Pharma. Bio. Sci*. *8*(2), 116-125.

López-Escudero, F. J., Del Río, C., Caballero, J. M., and Blanco-López, M. A. (2004). Evaluation of olive cultivars for resistance to *Verticillium dahliae*. *European Journal of Plant Pathology*. 110(1), 79-85.

Rashid, S., Charles, T. C., and Glick, B. R. (2012). Isolation and characterization of new plant growth-promoting bacterial endophytes. *Applied soil ecology*. *61*, 217-224.

Rehman, H. U., Siddique, N. N., Aman, A., Nawaz, M. A., Baloch, A. H., and Qader, S. A. U. (2015). Morphological and molecular based identification of pectinase producing *Bacillus licheniformis* from rotten vegetable. *Journal of Genetic Engineering and Biotechnology*. *13*(2), 139-144.

Schwyn, B., and Neilands, J. B. (1987). Universal chemical assay for the detection and determination of siderophores. *Analytical biochemistry*. *160*(1), 47-56.

Shahzad, R., Khan, A. L., Waqas, M., Ullah, I., Bilal, S., Kim, Y. H., et al. (2019). Metabolic and proteomic alteration in phytohormone-producing endophytic *Bacillus amyloliquefaciens* RWL-1 during methanol utilization. *Metabolomics*. *15*(2), 16.

Somerville, T. F., Corless, C. E., Sueke, H., Neal, T., and Kaye, S. B. (2020). 16S ribosomal RNA PCR versus conventional diagnostic culture in the investigation of suspected bacterial keratitis. *Translational vision science and technology*. *9*(13), 2-2.

Yamamoto, S., and Harayama, S. (1995). PCR amplification and direct sequencing of *gyrB* genes with universal primers and their application to the detection and taxonomic analysis of *Pseudomonas putida* strains. *Applied and environmental microbiology*. *61*(3), 1104-1109.

Zalila-Kolsi, I., Mahmoud, A. B., Ali, H., Sellami, S., Nasfi, Z., Tounsi, S., and Jamoussi, K. (2016). Antagonist effects of *Bacillus* spp. strains against *Fusarium graminearum* for protection of durum wheat (*Triticum turgidum* L. subsp. *durum*). *Microbiological Research*. *192*, 148-158.
