# Supplementary material for: The Phlorizin-Degrading Bacillus licheniformis XNRB-3 Mediates Soil Microorganisms to Alleviate Apple Replant Disease
Source: Front Microbiol. 2022 Mar 3;13:839484. doi: 10.3389/fmicb.2022.839484 (PMC8927668; doi:10.3389/fmicb.2022.839484)

**The Phlorizin-degrading *Bacillus licheniformis* XNRB-3 mediates soil microorganisms to alleviate apple replant disease**

Yanan Duan **^1^**, Lei Zhao**^1^**, Weitao Jiang**^1^**, Ran Chen**^1^**, Rong Zhang**^1^,** Xuesen Chen**^1^**, Chengmiao Yin**^1*^**, and Zhiquan Mao**^1*^**

^1^National Key Laboratory of Crop Biology, College of Horticulture Science and Engineering, Shandong Agricultural University, Shandong 271018, PR China

***Correspondence:**

Zhiquan Mao, Email: [mzhiquan@sdau.edu.cn](mailto:mzhiquan@sdau.edu.cn)

Chengmiao Yin, E-mail: [yinchengmiao@163.com](mailto:yinchengmiao@163.com)

**Supplementary information**

**Figure S1:** Optimal results of single-factor fermentation conditions. a: Effect of inoculation amount (3%-10%) on the growth of strain XNRB-3 under different time conditions. The abscissa is the fermentation time, the left ordinate is the OD_600_ value of the fermentation broth, and the right ordinate is the number of bacteria. b: Growth curve of strain XNRB-3 after optimization of fermentation conditions. The abscissa was the fermentation time, the ordinate was the OD_600_ value of the fermentation broth. c-i:The growth and antibacterial effect of strain XNRB-3 under single-factor fermentation. Using *Fusarium oxysporum* as the indicator bacteria to determine the inhibition rate. The abscissa is the fermentation condition, the left ordinate is the antibacterial rate, and the right ordinate is the OD_600_ value of the fermentation broth. Values in columns followed by the same letter are not significantly different according to Duncan test at *p* < 0.05. Values are mean ± SD (n=3).


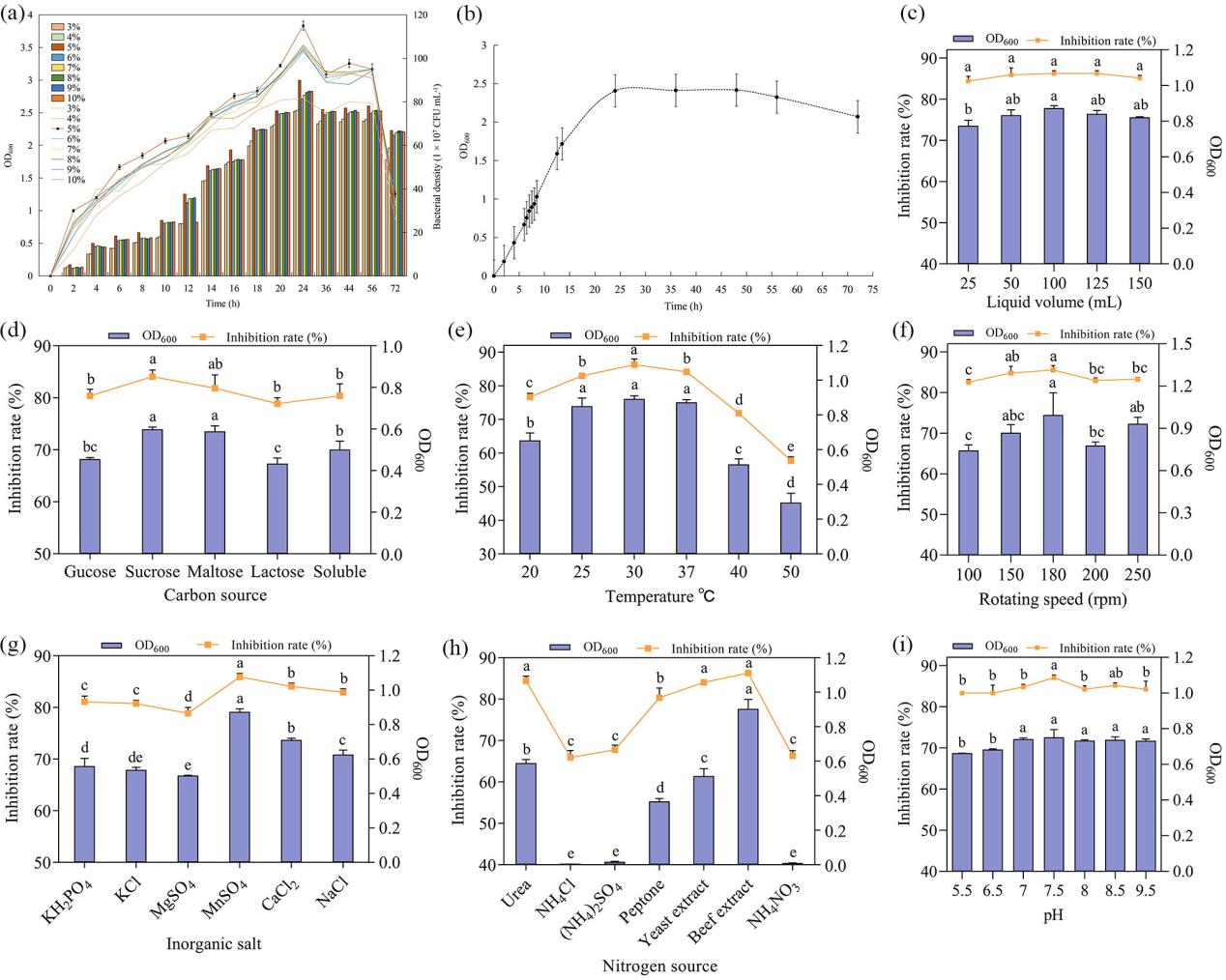


**Figure S2:** The isolated strain XNRB-3 was cultured on LB agar at 37 ℃ for 24 h (a) and the cellular morphological character were observed by Nikon fluorescence microscope BX-51 (b-c) and SU-8010 scanning electron microscope (d-i).


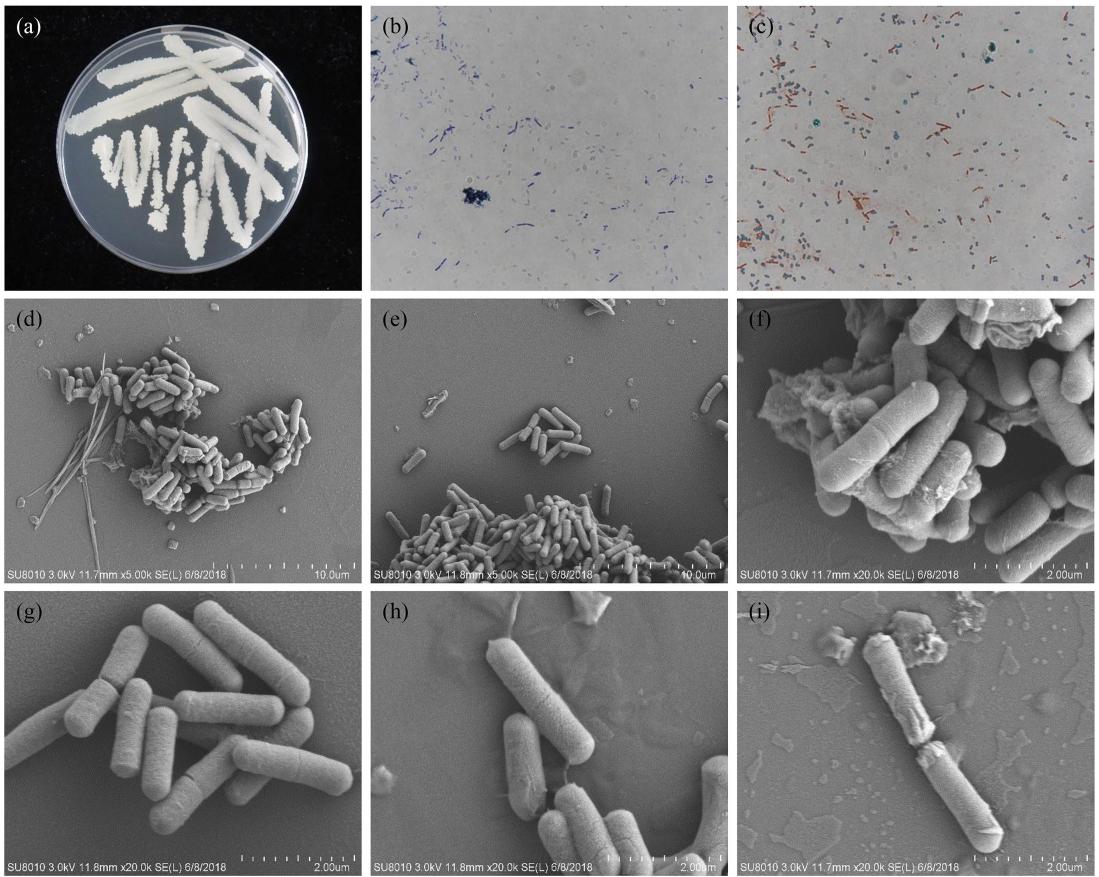


Note: a: Single colony morphology: the surface of the colony was white with irregular edges, and the surface was flat to convex, smooth, and opaque. b-c: The shape and size of the bacteria and spores: long and rod-shaped, ovoid spores (0.5–0.7) μm× (1.0–1.4) μm, mesophytic or subterminal, gram stain was positive, bar=100×/1.30 oil; d-i: The shape and size of the bacteria: rod-shaped, with blunt ends at both ends and without flagella, bacteria grew solitary and were paired or chain-like; (0.1–0.8) μm × (1.5–3.5) μm, d: bar=11.7 mm×5.00 SE (L), 10 μm. e: bar=11.8 mm×5.00 k SE (L), 10 μm. g, h: bar=11.8 mm×20.0 k SE (L), 2 μm. f,i: bar=11.7 mm×20.0 k SE (L), 2 μm.

**Figure S3:** The ML consensus tree inferred from the combined 16S rDNA, *gyrA*, *gyrB*, and *rpoB* sequence alignment. Support for each branch in the inferred tree was evaluated using 1000 bootstrap replications. Support values (ML bootstrap and posterior probability values) are indicated at the branches. The scale bar indicates 0.4 expected changes per site. Clade numbers and Latin name are provided on the right of the tree and these are used for reference in the treatment of the species. The combined sequence dataset included 35 ingroup taxa, with *Paenibacillus polymyxa* (BLB267) as the outgroup taxon. Strain XNRB-3 was indicated in bold and red.

**
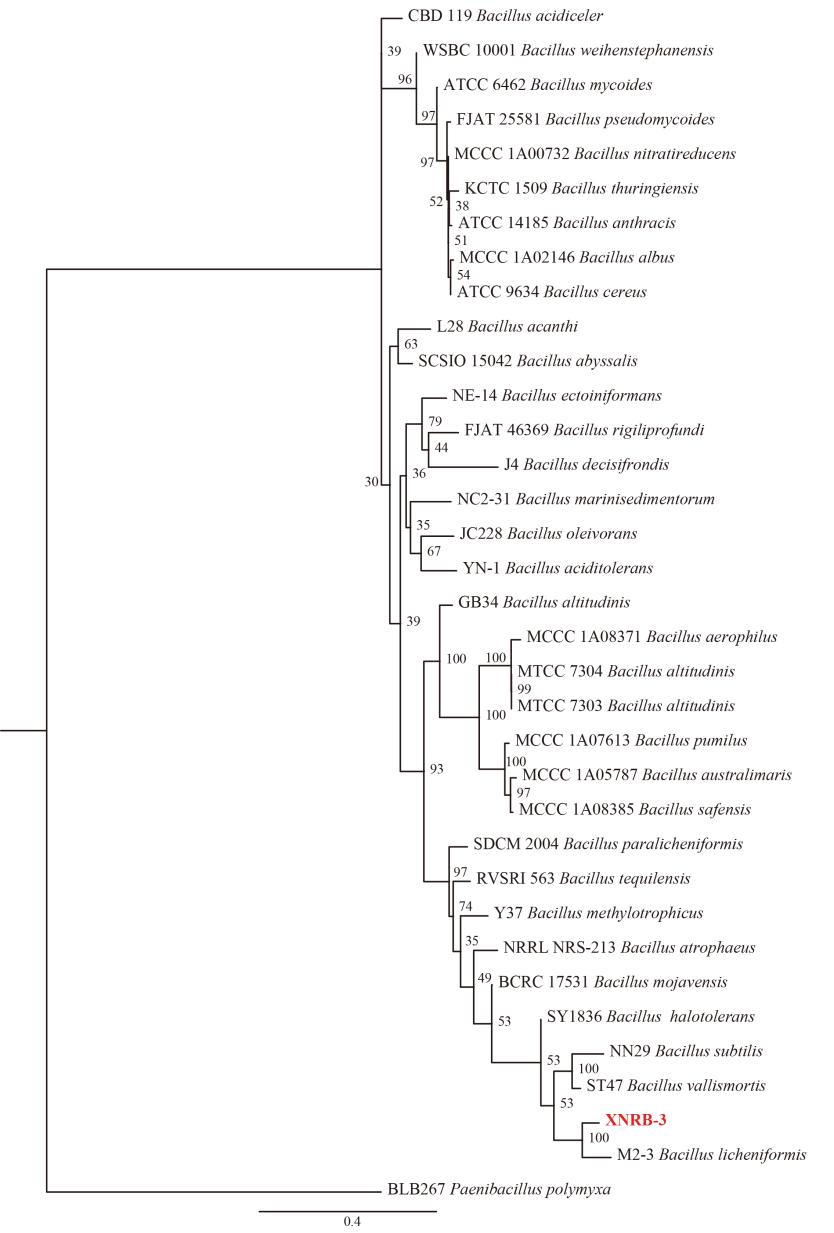
**

**Figure S4:** Strain XNRB-3 has a variety of plant growth promoting activities. a: Phosphate solubilization, b: Potassium solubilization, c: Nitrogenfixation, d: Chitinolytic activity, e: Siderophore production, f: Ammonia production, g: Pectinase activity, h: Amylase production, i: Cellulose activity, j: β1,3-glucanase activity, k: Protease activity, l: Chitosanase activity, m: Indole-3-acetic acid (IAA) production, n: The growth of strain XNRB-3 on different concentrations of phloridin inorganic salt medium. A: Control, B-D: 1 mmol L^-1^, E-G: 2 mmol L^-1^, H-J: 3 mmol L^-1^, K: 4 mmol L^-1^, L: 5 mmol L^-1^. o: Sensitivity of strain XNRB-3 to antibiotics. The ordinate is the concentration of antibiotics, and the abscissa is the number of strains. p: Standard curve of phlorizin. The ordinate is the absorbance value, and the abscissa is the concentration of Phlorizin. q: Degradation of phlorizin under different treatments. The ordinate is the concentration of phlorizin, and the abscissa is the days of inoculation.

**
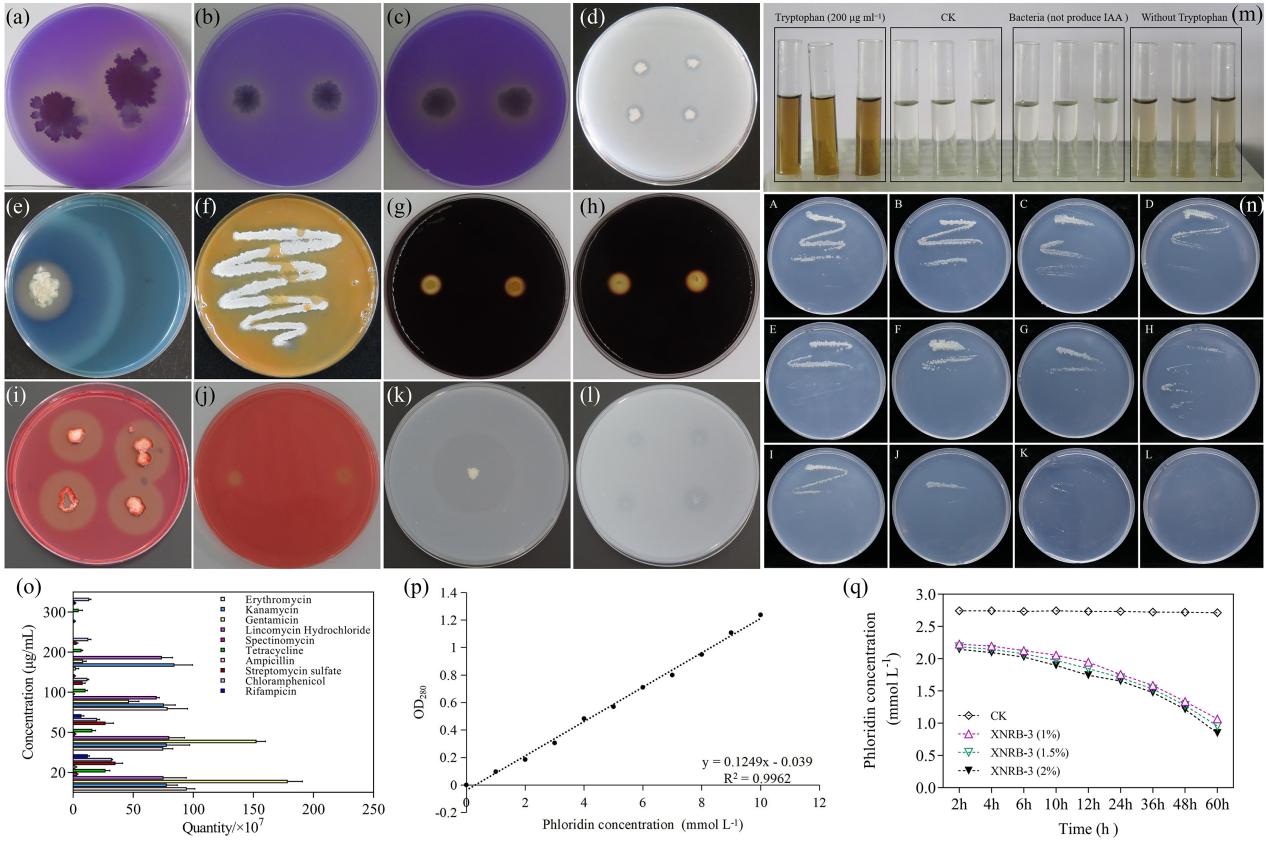
**

**Figure S5:** Amplification products of the lipopeptide genes. M: DL2000 Marker.

**
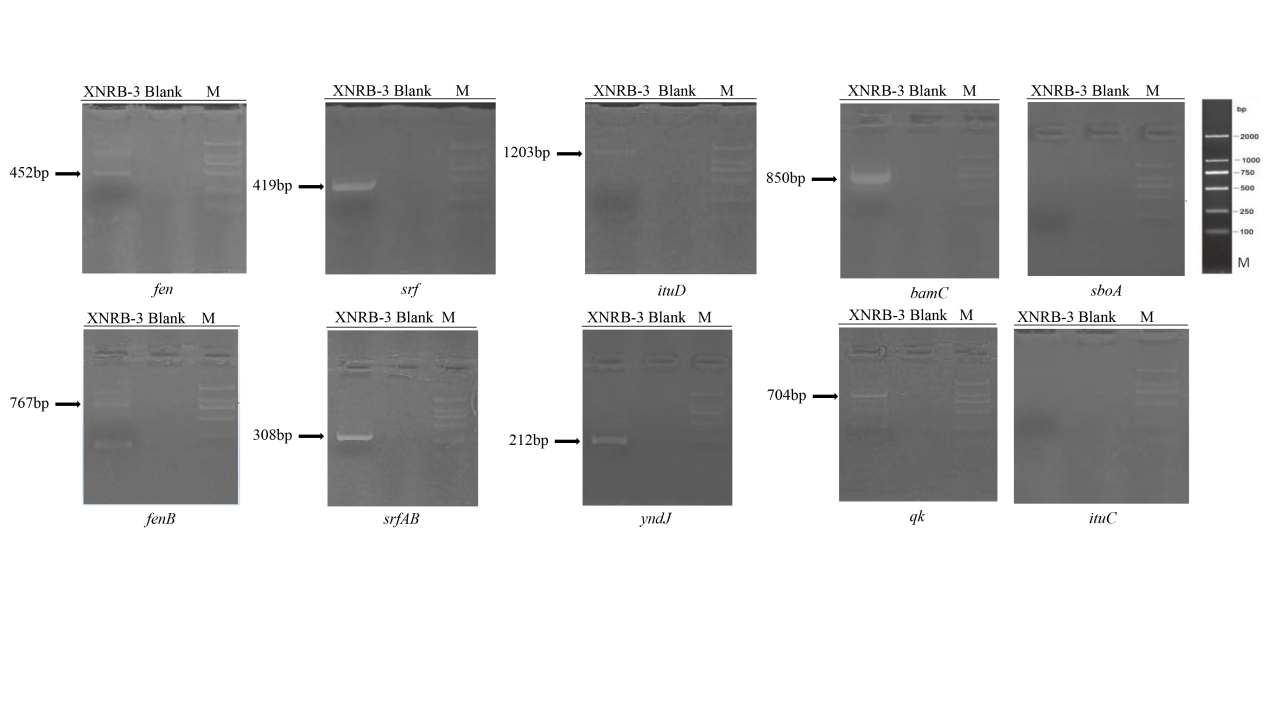
**

**Figure S6:** Response surface analysis three-dimensional and contour plot for inhibition zone diameter interaction. The shape of the response surface and the contour line can reflect the strength of interaction effects. Steeper response surfaces and greater proximity of the contour line to the ellipse are positively correlated with the significance of the effect.

**
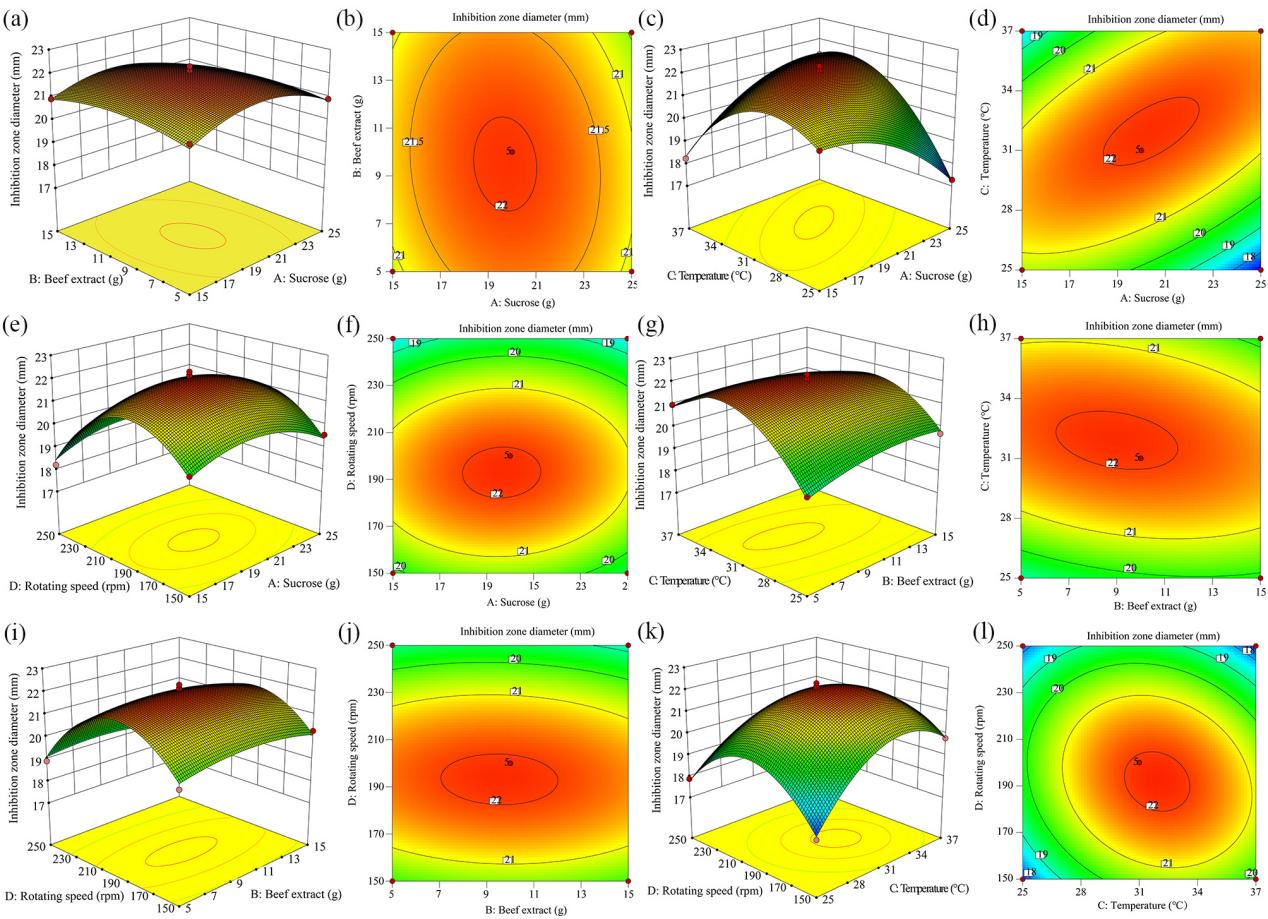
**

**Figure S7:** Total ion chromatogram of extracellular metabolites. The abscissa represents the peak time, and the ordinate represents the peak height. The figure is marked with retention time. After detection, select the Area % >0.62 and Retention index RI > 700 to mark.

**Figure S8:** Mass spectrum of the representative compounds. Mass spectrum of the compound obtained by GC-MS analysis was compared with the NIST17 spectral database. If the ion fragment size and height similarity of the mass spectrum are above 95, the molecular formula, molecular weight and molecular structure of the substance can be determined. The abscissa represents the mass of the ion; the ordinate represents the intensity of the ion current. The left side was the mass spectrum of the compound, and the right side was the result after comparison with NIST17 spectral database. a: 2,3-Butanediol (GC RT 11.867 min); b: 1,2-Benzenedicarboxylic acid, bis(1-methylethyl) ester (RT 28.205 min); c: 2,4-Di-tert-butylphenol (RT 25.263 min); d: Butanedioic acid, monomethyl ester (RT 17.705 min); e: alpha-Bisabolol (RT 26.613 min); f: Acetoin (RT 10.015 min); g: Dibutyl phthalate (RT 34.219 min); h: 3-Nonen-2-one (RT 19.886 min); i: Benzoic acid, 3,4-dimethyl-, methyl ester (RT 21.671 min).


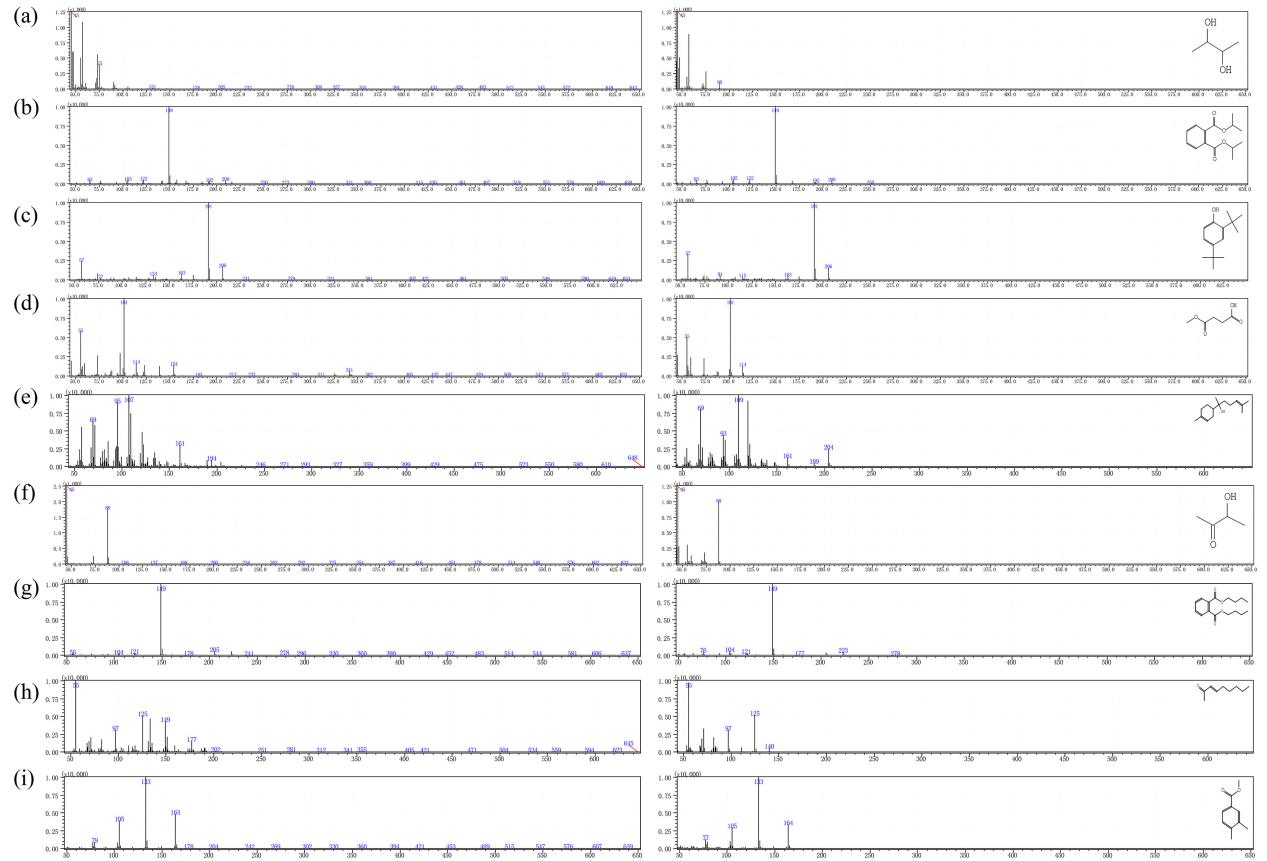


**Figure S9:** a: Microscopic observation of the longitudinal section of the plant root. *Fusarium verticillioides* and *Fusarium solani*: The roots of the plants were soaked with the conidia suspension for 12h. C-E, H, L, N, P Dense mycelium appeared in the epidermis of the root system, and the epidermal and cortical cells appeared ruptured, shedding and deformed (arrows), and the cells were arranged irregularly. M Cauliflower-like structure appeared in the infected root areas (arrows). A, F, K, O The conidia and hyphae of *Fusarium* appeared in the cortex and vascular column. B, I, J Viscous substances and starch granules appear in the cortex and vascular columns, which are densely accumulated on the cell wall. b: Population sizes of strain XNRB-3 recovered from root tissues of *Malus hupeheusis* Rehd. seedlings after root-dip inoculation with bacterial suspensions. Values in columns followed by the same letter are not significantly different according to Duncan test at *p* < 0.05. Values are mean ± SD (n=3). c: Effect of strain XNRB-3 on the number of *Fusarium* in the rhizosphere soil of plants at 7, 21, and 35 days after transplanting. CK: control, inoculated with *Fusarium*, XNRB-3: inoculated with *Fusarium* and the strain XNRB-3. ‘*’ above the columns indicate a significant difference at *p* < 0.05.

**
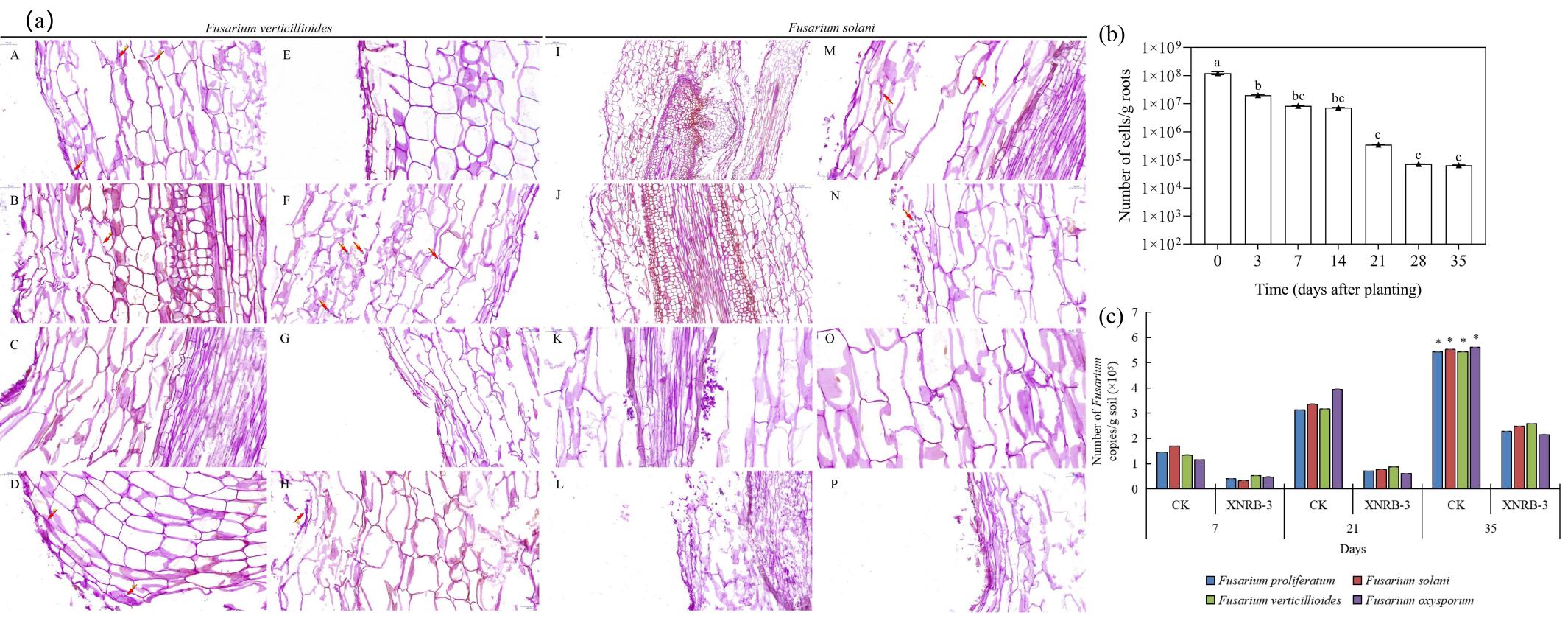
**

**Figure S10:** Microscopic observation of the plant root stained by PAS. Mock Sterile distilled water, FB fermentation broth. *F. proliferatum* and *F. oxysporum*: conidia suspension. PAS staining turns the polysaccharides on the fungal wall purple-red. Observation of plant root sections revealed that the root system was mainly composed of three parts from outside to inside: the epidermis, the cortex (the outer cortex, the cortical parenchyma, and the Kjeldahl belt), and the vascular column (the central sheath, phloem, and xylem). Mock The root tissue was intact, the cell boundaries are clear and neatly arranged. FB The conidia and hyphae of *Fusarium* were attached to the epidermis, the epidermis and cortex cells were slightly broken, deformed, and the internal tissue structure is intact (A-D). E, G, I, L Dense mycelium appeared in the epidermis of the root system, and the epidermal and cortical cells appeared ruptured, and deformed (arrows), and the cells were arranged irregularly. F Cauliflower-like structure appeared in the infected root areas (arrows). H, J-L The conidia and hyphae of *Fusarium* appeared in the cortex and vascular column. G Viscous substances and starch granules appear in the cortex and vascular columns, which are densely accumulated on the cell wall.

**
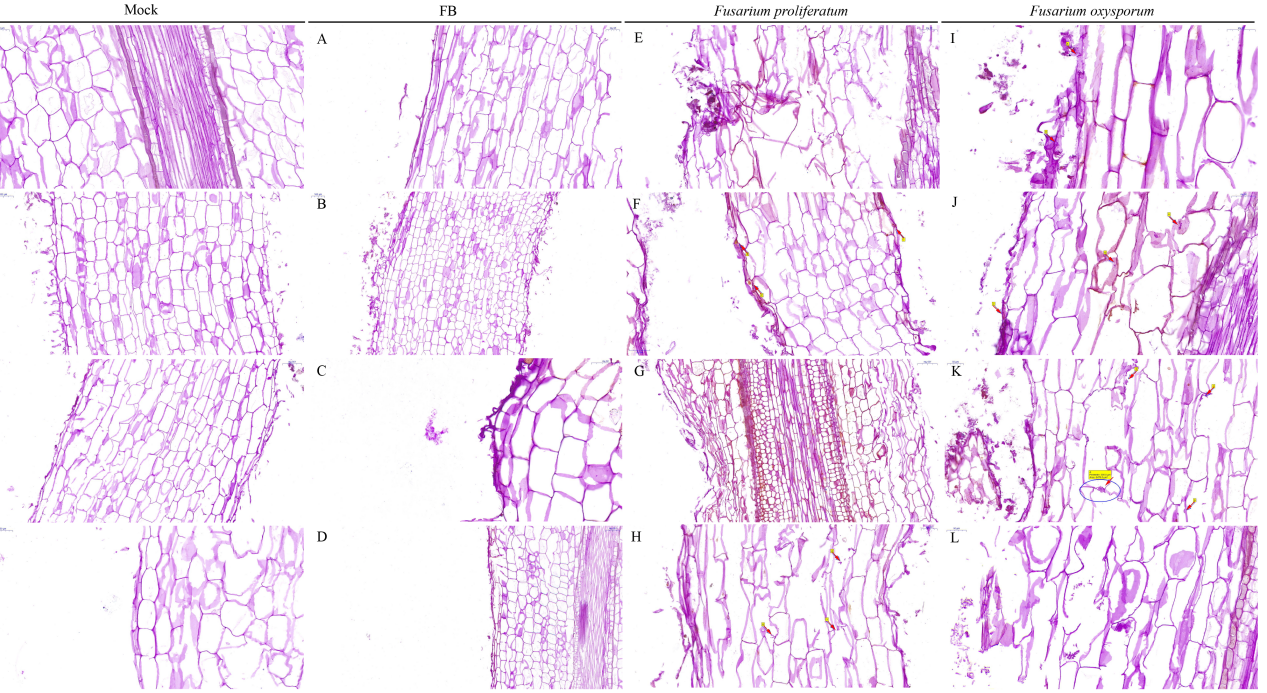
**

**Figure S11:** Effect of different treatments on seedling biomass of *Malus hupehensis* Rehd. seedlings, including plant height, ground diameter, fresh weight, and dry weight. Values in columns followed by the same letter are not significantly different according to Duncan test at *p* < 0.05. Values are mean ± SD (n=3). CK1: 31-year-old orchard soil, CK2: Methyl bromide fumigation, T1: Fertilizer carrier, T2: XNRB-3 bacterial fertilizer.

**
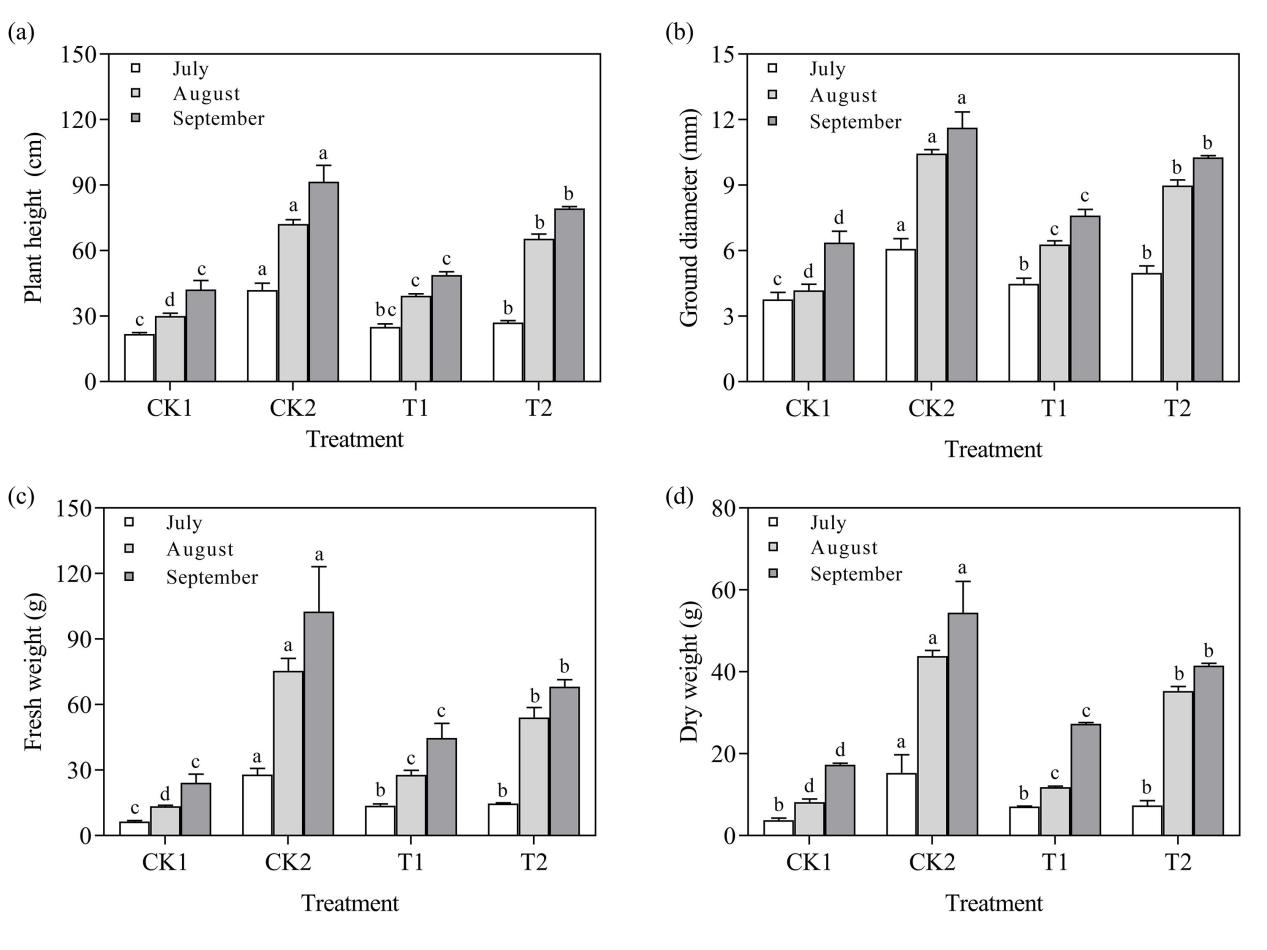
**

**Figure S12:** Effects of different treatments on the physical and chemical properties of rhizosphere soil in October 2021, including organic matter (%), total nitrogen (%), total phosphorus (g kg^-1^), total potassium (g kg^-1^), available potassium (mg kg^-1^), available phosphorus (mg kg^-1^), NH_4_^+^-N (mg kg^-1^), nitrate nitrogen (mg kg^-1^), and soil pH. Values in columns followed by the same letter are not significantly different according to Duncan test at *p* < 0.05. Values are mean ± SD (n=3). CK1: 28-year-old orchard soil, CK2: Methyl bromide fumigation, T1: Fertilizer carrier, T2: XNRB-3 bacterial fertilizer.


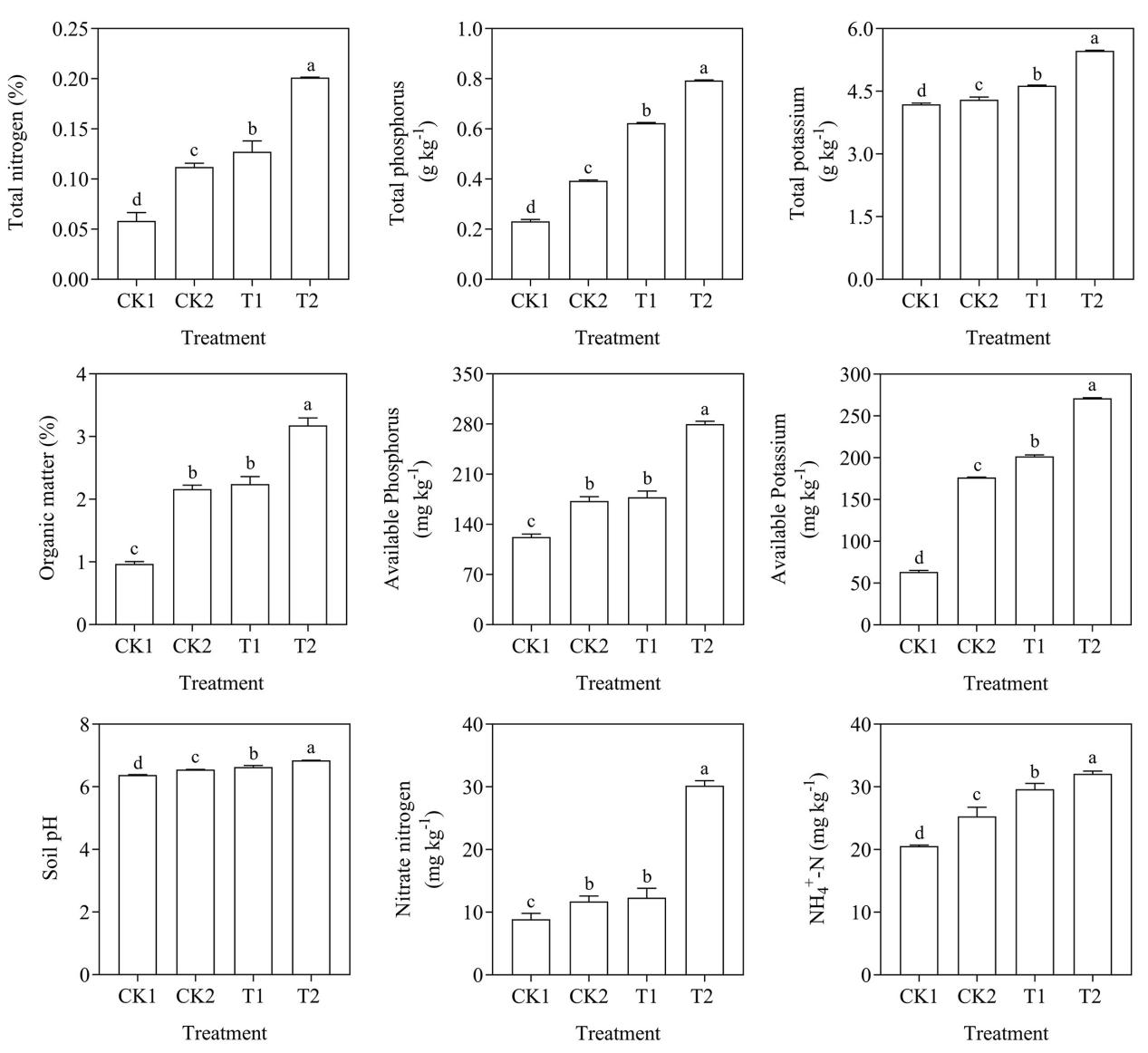

Supplement: Supplementary file 2 [file Data_Sheet_2.docx]
